# Build-up of double carbohelicenes using nitroarenes: dual role of the nitro functionality as an activating and leaving group†

**DOI:** 10.1039/d0sc02058c

**Published:** 2020-06-25

**Authors:** Fulin Zhou, Fujian Zhou, Rongchuan Su, Yudong Yang, Jingsong You

**Affiliations:** Key Laboratory of Green Chemistry and Technology of Ministry of Education, College of Chemistry, Sichuan University 29 Wangjiang Road Chengdu 610064 P. R. China jsyou@scu.edu.cn yangyudong@scu.edu.cn

## Abstract

The construction of double carbohelicenes is highly fascinating yet challenging work. Disclosed herein is a streamlined and simplified synthetic route to double carbohelicenes starting from nitroarenes through sequential nitro-activated *ortho*-C–H arylation, denitrative alkenylation and intramolecular cyclodehydrogenation. In this synthetic strategy, the nitro group plays a dual role namely as a leaving group for the denitrative alkenylation and as an activating group for *ortho*-C–H arylation, which is distinct from those of aryl halides in a conventional coupling reaction. In this work, the palladium-catalyzed Heck-type alkenylation of nitroarenes has been presented, in which the conventionally inert Ar–NO_2_ bond is cleaved. This work provides a novel synthetic strategy for polycyclic aromatic hydrocarbons (PAHs).

## Abstract 2

Disclosed herein is a concise route to double carbohelicenes starting from nitroarenes, in which the nitro group serves as an activating group for *ortho*-C–H arylation and a leaving group for the denitrative alkenylation.

## Graphical abstract

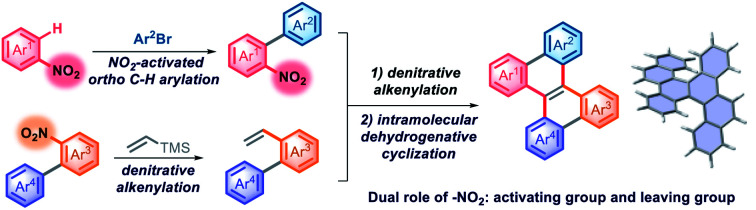

## Introduction

**Scheme 1:**
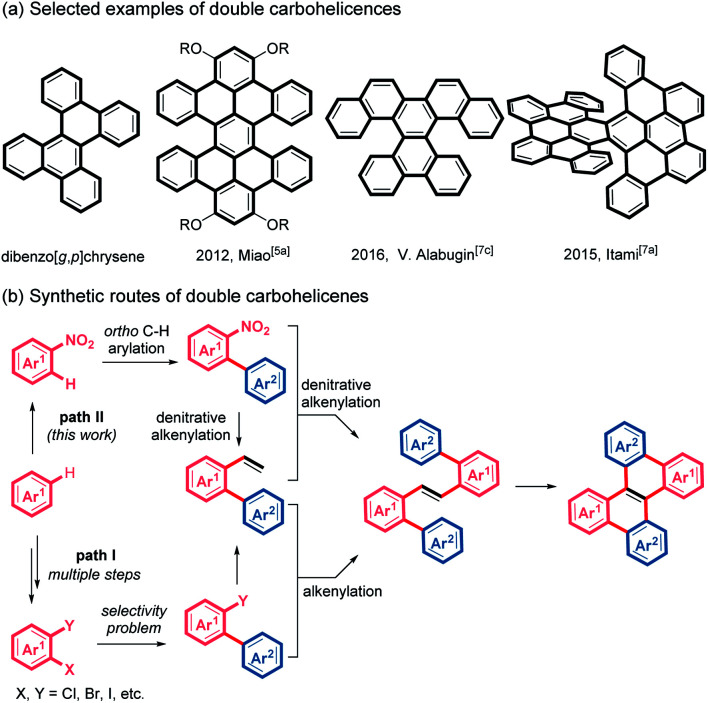
(a) Selected examples of double carbohelicenes. (b) Synthetic routes of double carbohelicenes.

Nonplanar polycyclic aromatic hydrocarbons (PAHs) have attracted increasing attention in the past decade because of their unique optoelectronic properties, fascinating supramolecular characteristics and conformational dynamics, distinct from those of planar PAHs.^1^ Helicenes, helical-shaped structures with *ortho*-fused benzene rings, are one of the most investigated nonplanar PAHs.^2,3^ In particular, considerable effort has been devoted to the helicenes with multihelicity, which can provide more diverse conformations, increased nonplanarity and multidimensional intermolecular interactions.^4^ Among these multi-helicenes, double helicenes, which usually exhibit effective intermolecular π–π stacking, are regarded as promising candidates for application in organic semiconductors and chiroptical devices.^5^ Although a variety of double helicenes have been reported (Scheme 1a),^5–7^ the synthesis of double carbohelicenes typically suffers from inaccessible precursors and tedious synthetic routes. Therefore, the development of facile preparation of double carbohelicenes starting from easily available substrates remains challenging yet highly demanded.

A retrosynthetic analysis of dibenzo[*g*,*p*]chrysene, the simplest double helicene, indicates that double carbohelicenes could be constructed by the Heck reaction between 2-(pseudo)halo-1,1′-biaryls and 2-vinyl-1,1′-biaryls, followed by intramolecular cyclization of the resultant 1,2-di([1,1′-biaryl]-2-yl)ethenes. However, the synthesis of both 2-vinyl-1,1′-biaryls and 2-(pseudo)halo-1,1′-biaryls typically requires multiple steps and the employment of di- or multi- halogenated aromatic substrates (Scheme 1b, path I).

Recently, the development of transition metal-catalyzed reactions using unconventional coupling partners has emerged as an attractive and challenging topic.^8^ Reactions involving the cleavage of inert chemical bonds can not only replenish the carbon–carbon bond formation toolbox but also improve synthetic approaches to complex structures. Nitroarenes, which could be easily prepared by nitration of aromatics, are common and cheap chemical feedstocks.^9^ Because of the strong electron-withdrawing nature of the nitro group, the *ortho*-position of nitroarenes easily undergoes C–H bond arylation with aryl halides *via* palladium catalysis.^10^ We envisioned that if a denitrative Heck-type reaction could be developed, double helicenes would be easily accessed from simple nitroarenes by *ortho*-C–H arylation, two consecutive denitrative alkenylation and intramolecular cyclodehydrogenation, which avoids the preparation of di- or multi- halogenated arenes (Scheme 1b, path II). However, because of the difficulty of oxidative addition of Ar–NO_2_ to a metal center, nitroarenes are typically inert in conventional transition metal-catalyzed coupling reactions.^11*c*^ Recently, pioneered by Nakao, the palladium-catalyzed denitrative amination, arylation, alkylation, alkynylation and hydrogenation of nitroarenes have been reported.^10*c*,11^ Herein we wish to describe a streamlined and simplified synthetic route to double carbohelicenes starting from nitroarenes based on our investigations on the palladium-catalyzed denitrative alkenylation of nitroarenes. During the preparation of this work, Yamaguchi and co-workers reported a palladium-catalyzed Mizoroki–Heck reaction of nitroarenes and styrene derivatives.^12^

## Results and discussion

Initially, 1-nitronaphthalene (**1a**) and styrene (**2a**) were selected as the model substrates for the condition optimization of catalytic denitrative alkenylation (Table S1†). After screening various reaction parameters, an optimal catalytic system composed of Pd(acac)_2_ (10 mol%), X-Phos (20 mol%), K_3_PO_4_ (3.0 equiv.) and heptane as the solvent was determined to give (*E*)-1-styrylnaphthalene (**3a**) in 93% yield (Table S1,† entry 1). No obvious amount of the (*Z*)-isomeric product was detected by GC-MS (*E*/*Z* > 99 : 1, Fig. S1†). Next, we investigated the substrate scope of the denitrative Heck-type reaction (Table 1). All the tested substrates gave the *E*-products with high stereoselectivity (*E*/*Z* ratios range from 93 : 7 to 99 : 1). No obvious electronic or steric effect of the substituents on the *E*/*Z*-selectivity was observed. Nitroarenes bearing the substituents with different electronic nature at the *ortho*-, *meta*-, or *para*-position could react with styrene to give the desired products in moderate to excellent yields (**3b–3k**). Nitro-substituted condensed aromatics such as 9-nitrophenanthrene, 3-nitroperylene and 1-nitroperylene also worked well (**3l–3n**). The scope of olefins was also examined. A variety of substituted phenyl ethylenes could participate in the denitrative Heck-type reaction to deliver the diaryl ethylenes (**4a–4e**). Vinyl naphthalenes and heteroaromatics such as 2-vinylpyridine and 2-vinylthiophene were accommodable (**4f–4i**). Furthermore, terminal conjugated dienes, aliphatic olefins and trimethyl(vinyl)silane were also applicable in this reaction (**4j–4l**).

**Table 1:** The scope of nitroarenes and olefins^a^

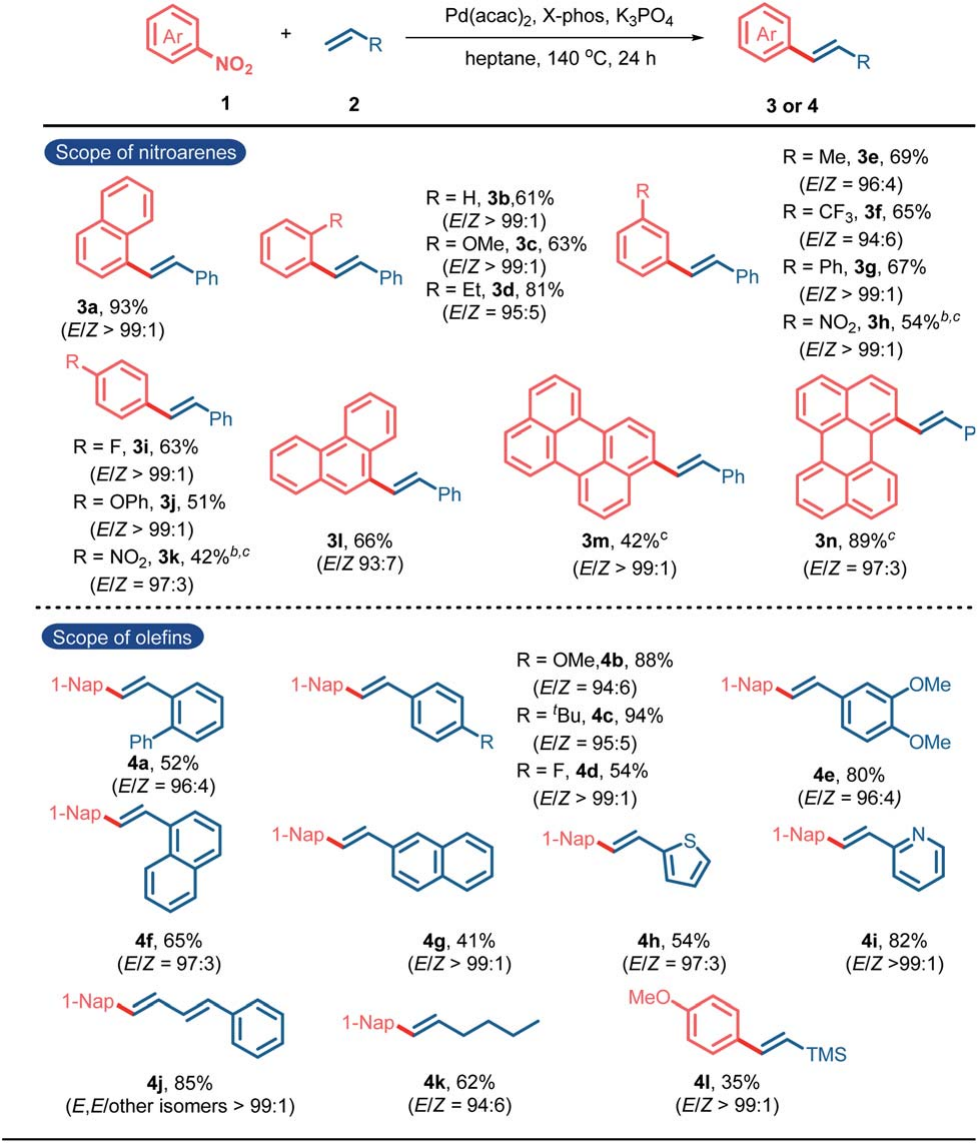

a Reaction conditions: **1** (0.6 mmol), **2** (0.2 mmol), Pd(acac)_2_ (10 mol%), X-Phos (20 mol%), and K_3_PO_4_ (3.0 equiv.) in heptane (1 mL) at 140 °C under N_2_ for 24 h. Yield of the isolated products. *E*/*Z* ratios were determined by ^1^H NMR spectroscopy.

b BrettPhos (20 mol%) instead of X-Phos.

c Nitroarene (0.2 mmol) and olefin (0.6 mmol). X-Phos = 2-(dicyclohexylphosphino)-2′,4′,6′-tri-*i*-propyl-1,1′-biphenyl; and Brettphos = 2-(dicyclohexylphosphino)-3,6-dimethoxy-2′-4′-6′-tri-*i*-propyl-1,1′-biphenyl sitylene.

**Scheme 2:**
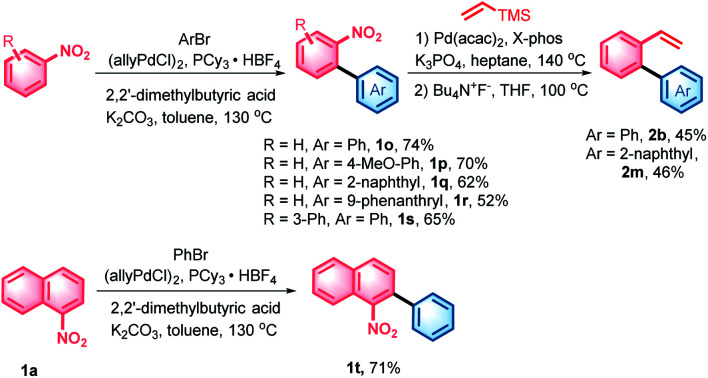
Synthesis of the key building blocks of double carbohelicenes. For detailed reaction conditions, see the ESI.†

**Scheme 3:**
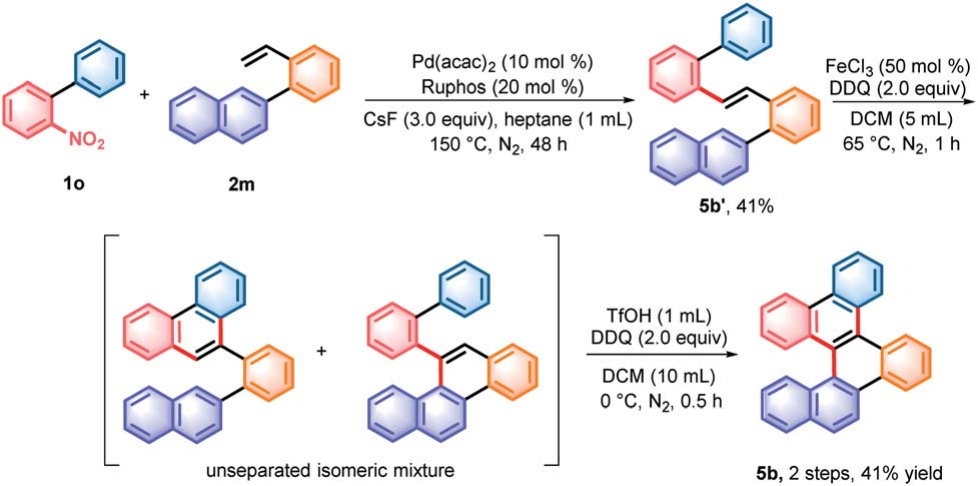
Synthesis of [4,5]helicene **5b**. TfOH = trifluoromethanesulfonic acid; DDQ = 2,3-dichloro-5,6-dicyano-1,4-benzoquinone; and Ruphos = 2-dicyclohexylphosphino-2′,6′-di-*i*-propoxy-1,1′-biphenyl.

With the denitrative Heck-type reaction in hand, the construction of double carbohelicences was next investigated. With the combination of nitro-activated *ortho*-C–H arylation and denitrative alkenylation, a library of 2-nitro-1,1′-biaryls and 2-vinyl-1,1′-biaryls, which are the key building blocks of double carbohelicenes, were easily prepared starting from simple nitroarenes (Scheme 2). Further denitrative alkenylation between the two structural units could provide the bis(biaryl) ethylene precursors. As a representative example, the reaction of **1o** and **2m** afforded the bis(biaryl) ethylene **5b′** in 41% yield (Scheme 3). The intramolecular oxidative cyclization in the presence of FeCl_3_ and DDQ resulted in an isomeric mixture of 5-([1,1′-biphenyl]-2-yl)chrysene and 9-(2-(naphthalen-2-yl)phenyl)phenanthrene, which were subsequently converted into [4,5]helicene **5b** in the presence of TfOH/DDQ (two steps, 41% yield).

**Scheme 4:**
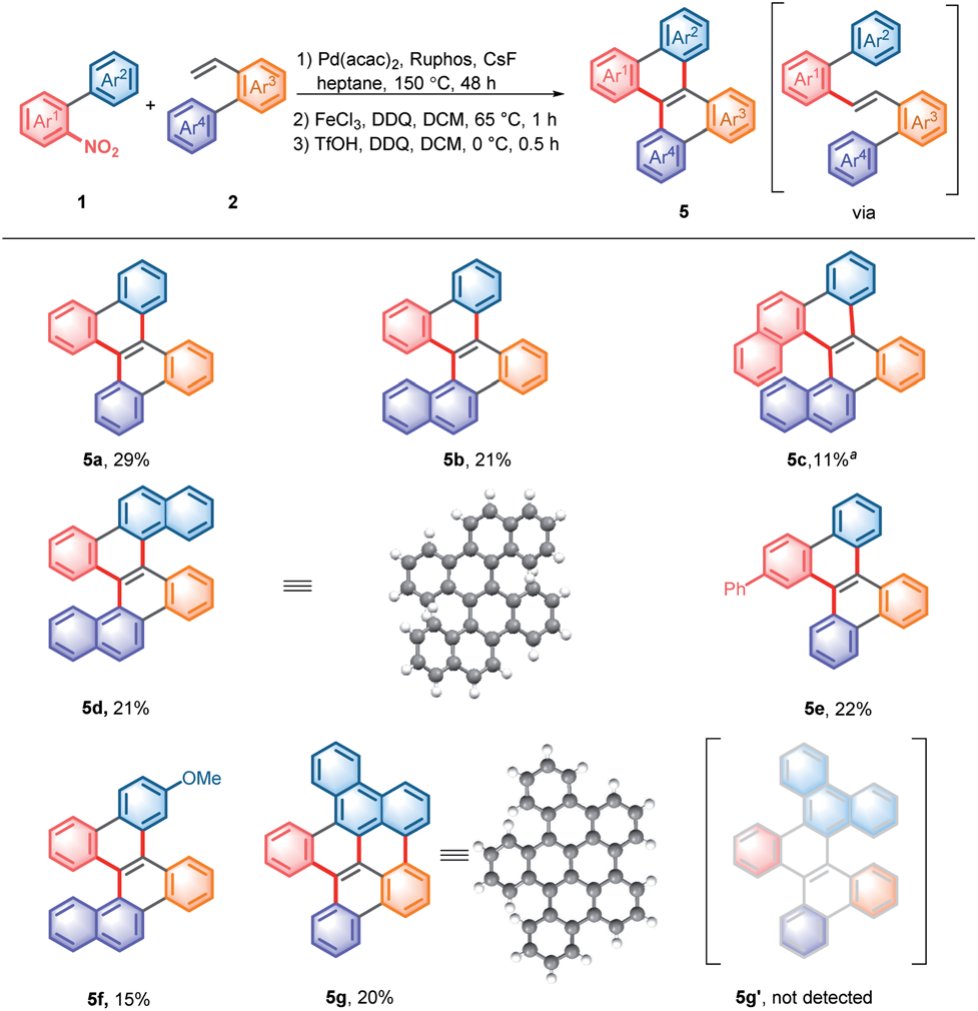
One pot synthesis of double carbohelicenes. Reaction conditions: (1) **1** (0.2 mmol), **2** (0.3 mmol), Pd(acac)_2_ (10 mol%), Ruphos (20 mol%), and CsF (3.0 equiv.) in heptane (1 mL) at 140 °C under N_2_ for 48 h; (2) FeCl_3_ (50 mol%), and DDQ (0.4 mmol) in DCM (5.0 mL) at 65 °C under N_2_ for 1 h; and (3) DDQ (0.4 mmol), TfOH (1.0 mL) and DCM (5.0 mL) at 0 °C under N_2_ for 0.5 h. ^*a*^ The third step was conducted at −10 °C.

In order to avoid troublesome isolation, a three-step, one pot procedure was developed. This process allowed the rapid construction of structurally diverse double carbohelicenes, including [4,4]helicene (**5a**), [4,5]helicene (**5b**), [4,6]helicene (**5c**), and [5,5]helicene (**5d**) (Scheme 4).^13^ Notably, the double carbohelicene **5d** was previously synthesized *via* sequential ICl-mediated aromatization and Pd-catalyzed intramolecular coupling starting from 1,2-bis(2-(naphthalen-2-yl)phenyl)ethyne, which was typically prepared using 1,2-dihalobenzene.^14^ Our protocol enables the synthesis of double carbohelicenes from simple nitroarenes rather than di- or multi- halogenated arenes. In the reaction of 9-(2-nitrophenyl)phenanthrene and 2-vinyl-1,1′-biphenyl, further coupling at the 1-position of phenanthrene was observed, leading to **5g** rather than **5g′**.^13^ Decreasing the amount of the oxidant or reaction temperature could not inhibit the undesired coupling reaction. In addition, substituted double helicenes could be easily prepared by using functionalized substrates, but a simple filtration through a silica gel pad before iron-induced carbocyclization was required (**5e** and **5f**).

**Fig. 1:**
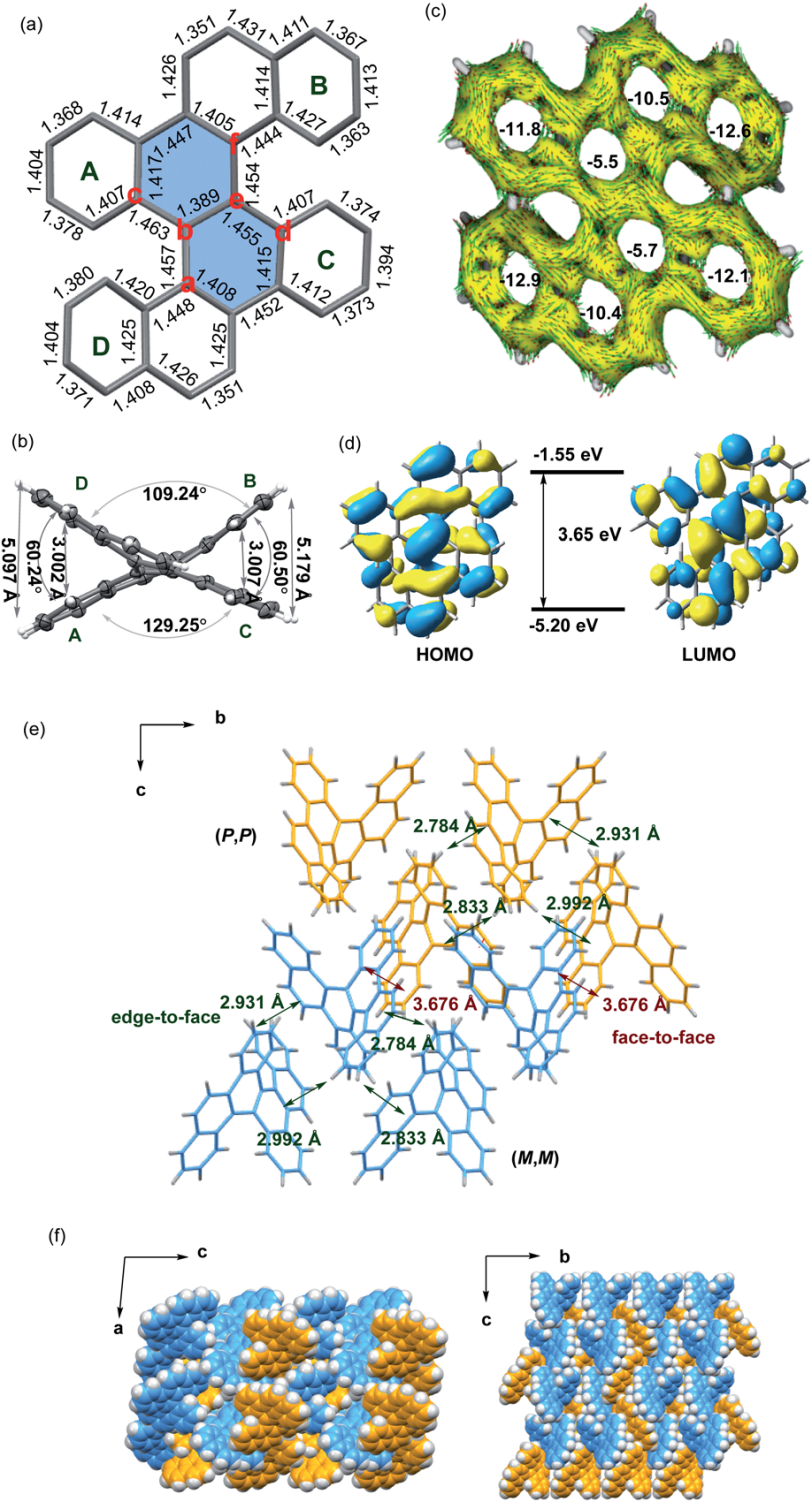
(a and e) Crystal structure of (*M*,*M*)-**5d**. (b) ORTEP drawing of (*M*,*M*)-**5d** with 50% probability. (c) Calculated ACID (only contributions from π-electrons of **5d** are considered) plots and NICS (0) values of **5d** at the B3LYP/6-31G(d) level. (d) Molecular orbitals of **5d**. (f) Crystal packings of **5d**. (*P*,*P*)-isomer, yellow; and (*M*,*M*)-isomer, blue.

The crystals of **5d** were grown by slowly volatilizing a saturated dichloromethane solution and the helical structure was clearly confirmed by X-ray crystallographic analysis (Fig. 1a).^13^ The geometry of helicene **5d** slightly deviates from the *C*_2_ symmetry, which is reflected by the unequal torsion angles of the two [5]helicene substructures. The dihedral angles between the terminal rings A and D and rings B and C are 60.2° and 60.5°, respectively (Fig. 1b), which are larger than those of recently presented double carbo[5]helicene (47.8°)^7*b*^ and OBO-fused double [5]helicene (48°).^5*d*^ Additionally, because of the repulsion of terminal benzene rings, the central naphthalene ring is distorted with dihedral angles of 32.0° for a–b–e–d and 31.8° for c–b–e–f, which are among the largest twisting deformations of the naphthalene ring in a nonplanar PAH.^15^ The embedded naphthalene unit (C–C bond lengths: 1.39–1.46 Å) possesses less aromaticity than pristine naphthalene (1.36–1.42 Å) presumably owing to the twisted structure. This observation is consistent with the computational results of the nucleus-independent chemical shift (NICS) and anisotropy of the induced current density (ACID) (Fig. 1c). As shown in Fig. 1d, the HOMO and LUMO energies of **5d** were estimated by molecular orbital (MO) calculation to be −5.20 and −1.55 eV, respectively, corresponding to a HOMO–LUMO gap of 3.65 eV, which is larger than that of the carbo[5]helicene (3.41 eV) and double carbo[5]helicenes reported by Miao (2.73 eV)^5*a*^ and Kamikawa (2.92 eV).^7*b*^ This result might be attributed to the decreased π-conjugation induced by the significant geometric distortion of the central naphthalene ring. In the crystal packing of **5d**, one unit cell contains four pairs of (*P*,*P*)- and (*M*,*M*)-enantiomers (Fig. 1e). The homochiral isomer layers lying on the *bc* plane stack alternatively along the *a*-axis (Fig. 1f), with a slipped face-to-face π–π distance of 3.67 Å to the adjacent heterochiral layer (Fig. 1e). In addition, every molecule interacts with the neighboring homochiral molecules through edge-to-face contact within the range of 2.78 to 2.99 Å (Fig. 1e).

The UV-vis absorption and fluorescence emission in dichloromethane of **5a–5g** were investigated (Fig. S3†). All the double carbohelicenes exhibit multiple absorption and blue fluorescence emission with the peaks in the range of 324–374 nm and 394–455 nm, respectively. A clear redshift of emission was observed with the extension of the π-system (Table S2,†**5a–5c**). The incorporation of functional groups such as phenyl and methoxy groups on the terminal rings of the helicene could improve the fluorescence quantum yield (Table S2,†**5a**, **5b**, **5e** and **5f**), which would be valuable for the future application of double carbohelicenes in optoelectronics and photonics.

**Fig. 2:**
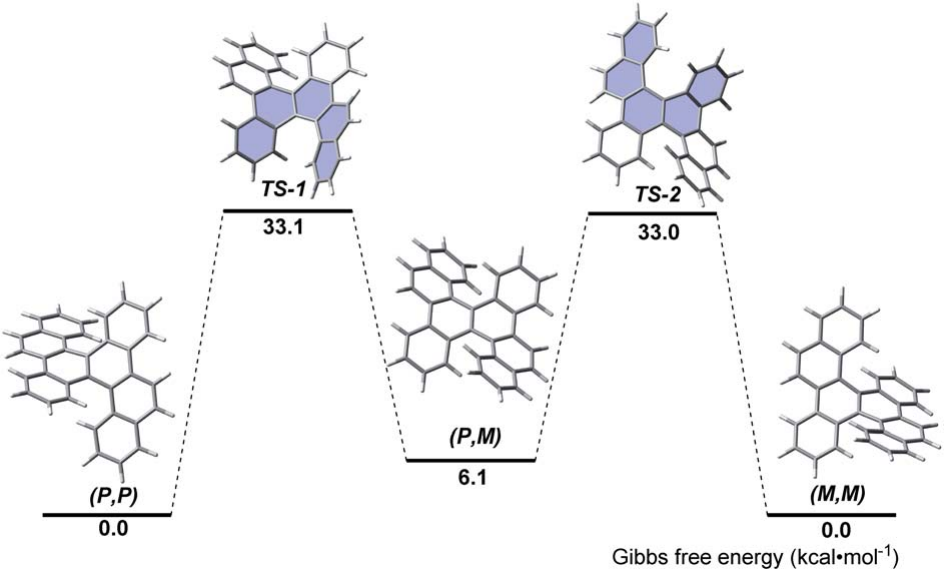
Isomerization process from (*P*,*P*)-**5d** to (*M*,*M*)-**5d**.

The thermal stability and isomerization process of **5d** were evaluated by DFT calculations at the B3LYP/6-31G(d) level (Fig. 2). The (*P*,*P*)- and (*M*,*M*)-enantiomers possess the same thermodynamic stability, but are more stable than the (*P*,*M*)-enantiomer. This result coincides with the fact that only (*P*,*P*)- and (*M*,*M*)-enantiomers were observed in the single crystals of **5d**, whereas no (*P*,*M*)-enantiomer was observed. The (*P*,*P*)-to-(*M*,*M*) interconversion proceeds through the transition states TS-1 and TS-2, in which the terminal benzene rings stretch outward and bend inward. The calculated activation free energy (approximately 33.0 kcal mol^−1^) was much higher than that of carbo[5]helicene (22.9 kcal mol^−1^),^16^ and slightly higher than that of the [5,5]helicenes reported by Miao (approximately 28.6 kcal mol^−1^)^5*a*^ and Kamikawa (31.8 kcal mol^−1^).^7*b*^ Because the calculated activation free energy approximates to that of carbo[6]helicene (35.0 kcal mol^−1^),^16^ the racemization process of **5d** might be slow.

## Conclusions

In conclusion, we have presented the promising potential of nitroarenes in the construction of polycyclic aromatic hydrocarbons (PAHs) based on our investigations on the palladium-catalyzed denitrative alkenylation reaction. The nitro group can act as a leaving group for the denitrative alkenylation and an activating group for *ortho*-C–H arylation, which provides a rapid access to double carbohelicenes through sequential nitro-activated *ortho*-C–H arylation, two consecutive denitrative alkenylation and intramolecular cyclodehydrogenation. Further applications of this strategy in the construction of other twisted and π-extended aromatic systems are underway in our laboratory.

## Conflicts of interest

There are no conflicts to declare.

## Supplementary Material

SC-011-D0SC02058C-s001

SC-011-D0SC02058C-s002
