# Supplementary material for: Build-up of double carbohelicenes using nitroarenes: dual role of the nitro functionality as an activating and leaving group
Source: Chem Sci. 2020 Jun 25;11(28):7424–8. doi: 10.1039/d0sc02058c (PMC8159353; doi:10.1039/d0sc02058c)

Electronic Supplementary Material (ESI) for Chemical Science.
This journal is © The Royal Society of Chemistry 2020

Supplementary Information

Build-Up of Double Carbohelicenes Using Nitroarenes: Dual Role of the Nitro Functionality as Activation and Leaving Group

Fulin Zhou, Fujian Zhou, Rongchuan Su, Yudong Yang and Jingsong You**

Key Laboratory of Green Chemistry and Technology of Ministry of Education, College of Chemistry, Sichuan University, 29 Wangjiang Road, Chengdu 610064, P. R. China

E-mail: *jsyou@scu.edu.cn*; *yangyudong@scu.edu.cn*

Content

I. General remarks..S1

II. Optimization of the denitrative Heck-type coupling reaction..S1

III. General procedure for the denitrative Heck-type reaction and synthesis of double carbohelicenes..S3

IV. Preparation and characterization of the described substances..S5

V. Single crystal X-ray structures of **5d** and **5g**..S21

VI. Photophysical properties of double carbohelicene **5**..S21

VII. Computational study..S23

VIII. References..S29

IX. Copies of NMR spectra..S30

I. General remarks

NMR spectra were recorded on an Agilent 400-MR DD2 spectrometer. The ^{1}H NMR (400 MHz) chemical shifts were recorded relative to $CDCl_3$, dimethyl sulfoxide-d_6 as the internal reference ($CDCl_3$: δ = 7.26 ppm; DMSO-d_6: δ = 2.50 ppm). The ^{13}C NMR (100 MHz) chemical shifts were given using $CDCl_3$ and dimethyl sulfoxide-d_6 as the internal standard ($CDCl_3$: δ = 77.16 ppm; DMSO-d_6: δ = 39.52 ppm). X-Ray single-crystal diffraction data were obtained on an Agilent Technologies Gemini plus single crystal diffraction. High-resolution mass spectra (HRMS) were obtained with a Shimadzu LCMS-IT-TOF (ESI). Gas chromatography-mass spectra (GC-MS) were obtained with a Shimadzu GCMS-QP2010 SE. UV/Vis spectra experiments were collected on a HITACHI U-2910. Fluorescence spectra were collected on a Horiba Jobin Yvon-Edison Fluoromax-4 fluorescence spectrometer. Unless otherwise noted, all reagents were obtained from commercial suppliers and used without further purification. $Pd(acac)_2$ was prepared according to the literature procedures.[1] The solvents were dried and purified using an Innovative Technology PS-MD-5 Solvent Purification System.

II. Optimization of the denitrative Heck-type coupling reaction

A 25 mL Schlenk tube with a magnetic stir bar was charged with $Pd(acac)_2$ (20 μmol, 10.0 mol%), ligand, base, styrene (23.0 μL, 0.2 mmol), 1-nitronaphthalene (103.8 mg, 0.6 mmol), and solvent (1.0 mL) under N_2. The resulting solution was stirred at room temperature for 10 min and then 140 ^{o}C for 24 h. Subsequently, it was diluted with 5 mL of dichloromethane. The solution was filtered through a celite pad and washed with 30 mL of dichloromethane. The filtrate was concentrated under reduced pressure and the residue was purified by column chromatography on silica gel to provide the desired product.

Table S1 Optimization for the synthesis of the (*E*)-1-styrylnaphthalene[a]

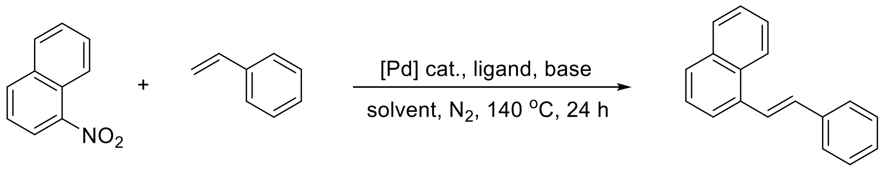

Entry	Catalyst (10 mol%)	Ligand (20 mol%)	Base (3.0 equiv)	Solvent	Isolated yield (%)
1	Pd(acac)$_2$	X-phos	K_3PO_4	heptane	93
2	$PdCl_2$	X-phos	K_3PO_4	heptane	10
3	Pd(OAc)$_2$	X-phos	K_3PO_4	heptane	trace
4	Pd(cod)Cl$_2$	X-phos	K_3PO_4	heptane	trace
5	Pd(TFA)$_2$	X-phos	K_3PO_4	heptane	n.r.
6	Pd(acac)$_2$	S-phos	K_3PO_4	heptane	32
7	Pd(acac)$_2$	Ruphos	K_3PO_4	heptane	56
8	Pd(acac)$_2$	Davephos	K_3PO_4	heptane	38
9	Pd(acac)$_2$	PCy_3	K_3PO_4	heptane	n.r.
10	Pd(acac)$_2$	dppb	K_3PO_4	heptane	n.r.
11	Pd(acac)$_2$	Brettphos	K_3PO_4	heptane	95
12	Pd(acac)$_2$	X-phos	K_3PO_4	toluene	78
13	Pd(acac)$_2$	X-phos	K_3PO_4	THF	69
14	Pd(acac)$_2$	X-phos	K_3PO_4	DMSO	n.r.
15	Pd(acac)$_2$	X-phos	K_2HPO_4	toluene	n.r.
16	Pd(acac)$_2$	X-phos	KH_2PO_4	toluene	n.r.
17	Pd(acac)$_2$	X-phos	Cs_2CO_3	heptane	62
18	Pd(acac)$_2$	X-phos	CsF	heptane	80
19[b]	Pd(acac)$_2$	X-phos	K_3PO_4	heptane	64
20[c]	Pd(acac)$_2$	X-phos	K_3PO_4	heptane	65
21[d]	Pd(acac)$_2$	X-phos	K_3PO_4	heptane	74

[a] Reaction conditions: **1a** (0.6 mmol), **2a** (0.2 mmol), [Pd] catalyst (10 mol%), ligand (20 mol%), base (3 equiv) and solvent (1 mL) at 140 °C under N_2 for 24 h. [b] Reaction at 120 °C. [c] Reaction at 130 °C. [d] Reaction for 12 h. X-phos = 2-(dicyclohexylphosphino)-2',4',6'-tri-*i*-propyl-1,1'-biphenyl; S-phos = 2-dicyclohexylphosphino-2',6'-dimethoxy-1,1'-biphenyl; Ruphos = 2-dicyclohexylphosphino-2',6'-di-*i*-propoxy-1,1'-biphenyl; Davephos = 2-(dicyclohexylphosphino)-2'-(*N*,*N*-dimethylamino))-1,1'-biphenyl; PCy_3 = tricyclohexyl

phosphine; dppb = 1,4-bis(diphenylphosphino)butane, Brettphos = 2-(dicyclohexylphosphino)-3,6-dimethoxy-2'-4'-6'-tri-*i*-propyl-1,1'-biphenyl; THF = tetrahydrofuran, DMSO = dimethyl sulfoxide.

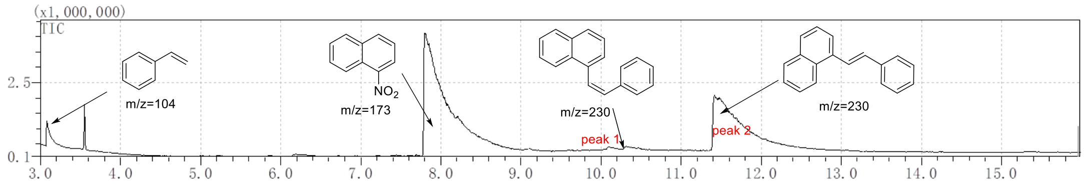

peak	retention time	peak area	peak area(%)
1	10.31	511289	0.04
2	11.42	1301609291	99.96

Fig. S1 The *E*/*Z* isomer ratio detected by gas chromatography-mass spectrometer.

III. General procedure for the denitrative Heck-type reaction and synthesis of double carbohelicenes

General procedure I: the denitrative Heck-type reaction of nitroarenes

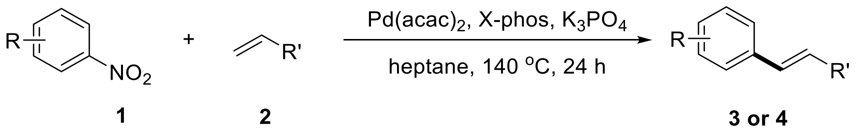

A 25 mL Schlenk tube with a magnetic stir bar was charged with $Pd(acac)_2$ (10.0 mol%), alkene **1** (0.2 mmol, 1.0 equiv), nitroarene **2** (3.0 equiv), X-phos (20.0 mol%), K_3PO_4 (3.0 equiv) and heptane (1.0 mL) under a N_2 atmosphere. The resulting solution was stirred at room temperature for 10 min and then 140 °C for 24 h. Subsequently, it was diluted with 5 mL of dichloromethane. The solution was filtered through a celite pad and washed with 30 mL of dichloromethane. The filtrate was concentrated under reduced pressure and the residue was purified by column chromatography on silica gel to provide the desired product.

General procedure II: the synthesis of 2-nitro-1,1'-biaryls

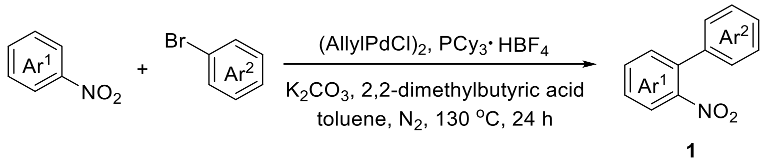

2-Nitro-1,1'-biaryls were prepared according to the literature procedures.[2] A 25 mL Schlenk tube with a magnetic stir bar was charged with nitroarenes, aryl bromide, $(allylPdCl)_2$ (5 mol%), $PCy_3{\cdot}HBF_4$ (15 mol%), K_2CO_3 (2.0 equiv), 2,2-dimethylbutyric acid (0.3 equiv), and toluene (2.0 mL) under N_2. The resulting solution was stirred at room temperature for 10 min and then 140 °C for 24 h. Subsequently, it was diluted with 5 mL of dichloromethane. The solution was filtered

through a celite pad and washed with 80 mL of dichloromethane. The filtrate was concentrated under reduced pressure and the residue was purified by column chromatography on silica gel to provide the desired product.

General procedure III: the synthesis of 2-vinyl-1,1'-biaryls

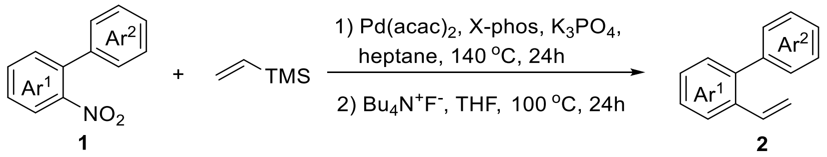

A 25 mL Schlenk tube with a magnetic stir bar was charged with 2-nitro-1,1'-biaryls (0.2 mmol, 1.0 equiv), vinyltrimethylsilane (3.0 equiv), $Pd(acac)_2$ (10 mol%), X-phos (20 mol%), K_3PO_4 (3.0 equiv), and heptane (1.0 mL) under N_2. The resulting solution was stirred at room temperature for 10 min and then 140 °C for 24 h. Subsequently, the solvent was removed under reduced pressure. Then, THF (2.0 mL) and tetrabutylammonium fluoride (10.0 equiv) were added under N_2. The resulting solution was stirred at 100 °C for 24 h. After that, the reaction was diluted with 5 mL of dichloromethane. The solution was filtered through a celite pad and washed with 30 mL of dichloromethane. The filtrate was concentrated under reduced pressure and the residue was purified by column chromatography on silica gel to provide the desired product.

General procedure IV: the synthesis of double carbohelicenes

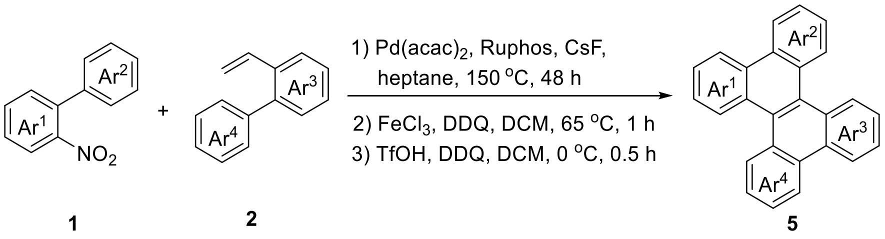

A 25 mL Schlenk tube with a magnetic stir bar was charged with 2-nitro-1,1'-biaryl (0.2 mmol, 1.0 equiv), 2-vinyl-1,1'-biaryl (1.5 equiv), $Pd(acac)_2$ (10 mol%), Ruphos (20 mol%), CsF (3.0 equiv), and heptane (1.0 mL) under a N_2 atmosphere. The resulting solution was stirred at room temperature for 10 min and then 150 °C for 48 h. Subsequently, it was diluted with 5 mL of dichloromethane. The solution was filtered through a celite pad and washed with 50 mL of dichloromethane. The filtrate was evaporated under reduced pressure. Then the residue was dissolved in dry dichloromethane (5.0 mL) and transferred to a dry Schlenk tube charged with $FeCl_3$ (50 mol%) and 2,3-dichloro-5,6-dicyano-1,4-benzoquinone (DDQ) (2.0 equiv) under N_2. The resulting solution was stirred 65 °C for 1 h. After that, the reaction was cooled to 0 °C and added DDQ (2.0 equiv), trifluoromethanesulfonic acid (TfOH) (1.0 mL) and dry dichloromethane (5.0 mL) under N_2. The mixture was stirred at 0 °C for another 30 min. Then the reaction was quenched with 5 mL of water. The mixture was extracted with dichloromethane (3 × 15 mL). The combined organic phase was dried over anhydrous sodium sulfate and filtered. The filtrate was concentrated under reduced pressure and the residue was purified by column chromatography on silica gel to provide the desired

product.

IV. Preparation and characterization of the described substances

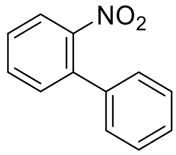

2-Nitro-1,1'-biphenyl (1o)

Following the general procedure II. Nitrobenzene (204.3 μL, 2 mmol) and bromobenzene (105.3 μL, 1 mmol) were used. Purification via flash column chromatography on silica gel (petroleum ether/ethyl acetate = 5:1) afforded **1o** as a light yellow solid (147.3 mg, 74% yield). ^{1}H NMR ($CDCl_3$, 400 MHz): δ (ppm) 7.86 (dd, J = 8.0, 1.2 Hz, 1H), 7.62 (td, J = 7.6, 1.2 Hz, 1H), 7.51 – 7.40 (m, 5H), 7.34 – 7.31 (m, 2H). ^{13}C NMR ($CDCl_3$, 100 MHz): δ (ppm) 147.6, 137.5, 136.5, 132.4, 132.1, 128.8, 128.4, 128.3, 128.0, 124.2. HRMS (ESI$^+$): calcd for $C_{12}H_9NO_2Na^+$ $[M+Na]^+$ 222.0525; found 222.0524

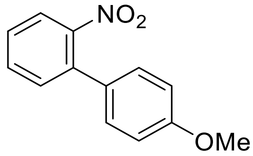

4'-Methoxy-2-nitro-1,1'-biphenyl (1p)

Following the general procedure II. Nitrobenzene (204.3 μL, 2 mmol) and 1-bromo-4-methoxybenzene (125.2 μL, 1 mmol) were used. Purification via flash column chromatography on silica gel (petroleum ether/ethyl acetate = 4:1) afforded **1p** as a yellow solid (160.4 mg, 70% yield). ^{1}H NMR ($CDCl_3$, 400 MHz): δ (ppm) 7.80 (d, J = 8.4 Hz, 1H), 7.61 – 7.57(m, 1H), 7.50 – 7.42 (m, 2H), 7.28 – 7.24 (m, 2H), 6.98 – 6.94 (m, 2H), 3.85 (s, 3H). ^{13}C NMR ($CDCl_3$, 100 MHz): δ (ppm) 159.8, 136.0, 132.3, 132.0, 129.6, 129.2, 127.9, 124.2, 114.33, 114.26, 55.4. HRMS (ESI$^+$): calcd for $C_{13}H_{11}NO_3Na^+$ $[M+Na]^+$ 252.0631; found 252.0626.

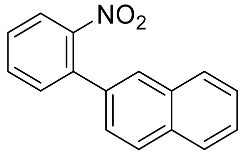

2-(2-Nitrophenyl)naphthalene (1q)

Following the general procedure II. Nitrobenzene (204.4 μL, 2 mmol) and 2-bromonaphthalene (206.0 mg, 1 mmol) were used. Purification via flash column chromatography on silica gel (petroleum ether/ethyl acetate = 5:1) afforded **1q** as a yellow solid (154.4 mg, 62% yield). ^{1}H NMR

($CDCl_3$, 400 MHz): δ (ppm) 7.93 (dd, J = 8.0, 1.2 Hz, 1H), 7.90 – 7.86 (m, 3H), 7.82 (d, J = 1.2 Hz, 1H), 7.66 (td, J = 7.6, 1.2 Hz, 1H), 7.56 – 7.50 (m, 4H), 7.41 (dd, J = 8.4, 1.6 Hz, 1H). ^{13}C NMR ($CDCl_3$, 100 MHz): δ (ppm) 149.5, 136.6, 135.1, 133.4, 133.0, 132.6, 132.4, 128.5, 128.4, 128.3, 127.9, 127.1, 126.71, 126.70, 125.9, 124.4. HRMS (ESI$^+$): calcd for $C_{16}H_{11}NO_2Na^+$ $[M+Na]^+$ 272.0682; found 272.0681.

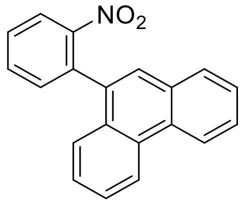

9-(2-Nitrophenyl)phenanthrene (1r)

Following the general procedure II. Nitrobenzene (102.2 μL, 1 mmol) and 9-bromophenanthrene (512.0 mg, 2 mmol) were used. Purification via flash column chromatography on silica gel (petroleum ether/ethyl acetate = 5:1) afforded **1r** as a yellow solid (155.5 mg, 52% yield). ^1H NMR ($CDCl_3$, 400 MHz): δ (ppm) 8.78 (d, J = 8.4 Hz, 1H), 8.74 (d, J = 8.4 Hz, 1H), 8.14 (dd, J = 8.4, 1.2 Hz, 1H), 7.87 (dd, J = 8.0, 1.2 Hz, 1H), 7.75 – 7.61 (m, 6H), 7.56 – 7.47 (m, 3H). ^{13}C NMR ($CDCl_3$, 100 MHz): δ (ppm) 149.8, 135.6, 134.7, 133.3, 133.0, 131.3, 130.8, 130.5, 130.4, 128.93, 128.90, 127.2, 127.10, 127.05, 126.9, 125.9, 124.5, 123.2, 122.8. HRMS (ESI$^+$): calcd for $C_{20}H_{13}NO_2Na^+$ $[M+Na]^+$ 322.0838; found 322.0840.

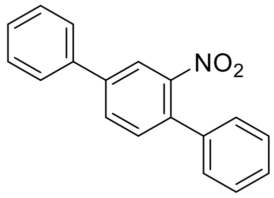

2'-Nitro-1,1':4',1''-terphenyl (1s)

Following the general procedure II. 3-Nitro-1,1'-biphenyl (199.1mg, 1 mmol) and bromobenzene (210.6 μL, 2 mmol) were used. Purification via flash column chromatography on silica gel (petroleum ether/ethyl acetate = 5:1) afforded **1s** as a brownness solid (178.8 mg, 65% yield). ^1H NMR ($CDCl_3$, 400 MHz): δ (ppm) 8.07 (d, J = 2.0 Hz, 1H), 7.84 (dd, J = 8.0, 2.0 Hz, 1H), 7.66 – 7.64 (m, 2H), 7.51 (t, J = 8.0 Hz, 3H), 7.47 – 7.42 (m, 4H), 7.38 – 7.36 (m, 2H). ^{13}C NMR ($CDCl_3$, 100 MHz): δ (ppm) 141.7, 138.4, 137.2, 135.0, 132.5, 130.8, 129.3, 128.9, 128.7, 128.4, 128.1, 127.2, 122.7. HRMS (ESI$^+$): calcd for $C_{18}H_{13}NO_2Na^+$ $[M+Na]^+$ 298.0838; found 298.0836.

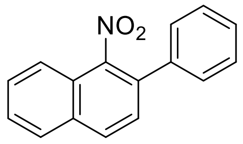

1-Nitro-2-phenylnaphthalene (1t)

Following the general procedure II. 1-Nitronaphthalene (173.0 mg, 1 mmol) and bromobenzene (210.6 μL, 2 mmol) were used. Purification via flash column chromatography on silica gel (petroleum ether/ethyl acetate = 5:1) afforded **1t** as a brown solid (176.9 mg, 71% yield). ^{1}H NMR ($CDCl_3$, 400 MHz): δ (ppm) 8.03 (d, J = 8.4 Hz, 1H), 7.96 (d, J = 7.6 Hz, 1H), 7.81 – 7.79 (m, 1H), 7.69 – 7.60 (m, 2H), 7.53 (d, J = 8.4 Hz, 1H), 7.49 – 7.45 (m, 5H). ^{13}C NMR ($CDCl_3$, 100 MHz): δ (ppm) 136.7, 134.8, 133.0, 131.6, 130.6, 129.04, 129.02, 128.8, 128.4, 128.2, 127.62, 127.6, 124.59, 121.9. HRMS (ESI^{+}): calcd for $C_{16}H_{11}NO_2Na^{+}$ $[M+Na]^{+}$ 272.0682; found 272.0674.

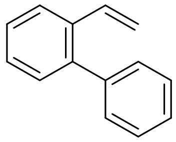

2-Vinyl-1,1'-biphenyl (2b)

Following the general procedure III. 2-Nitro-1,1'-biphenyl (39.8 mg, 0.2 mmol) and trimethyl(vinyl)silane (87.9 μL, 0.6 mmol) were used. Purification via flash column chromatography on silica gel (petroleum ether) afforded **2b** as colorless oil (16.2 mg, 45% yield). ^{1}H NMR ($CDCl_3$, 400 MHz): δ (ppm) 7.66 (dd, J = 6.4, 2.8 Hz, 1H), 7.44 – 7.40 (m, 2H), 7.38 – 7.33 (m, 5H), 7.32 – 7.29 (m, 1H), 6.72 (dd, J = 17.6, 11.2 Hz, 1H), 5.71 (dd, J = 17.6, 1.2 Hz, 1H), 5.19 (dd, J = 11.2, 1.2 Hz, 1H). ^{13}C NMR ($CDCl_3$, 100 MHz): δ (ppm) 141.0, 136.0, 135.9, 130.2, 129.9, 128.2, 127.8, 127.6, 127.1, 125.8, 114.8. HRMS (ESI^{+}): calcd for $C_{14}H_{12}Na^{+}$ $[M+Na]^{+}$, 203.0831; found 203.0832.

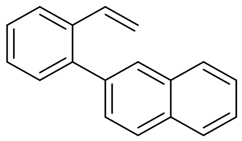

2-(2-Vinylphenyl)naphthalene (2m)

Following the general procedure III. 2-(2-Nitrophenyl)naphthalene (49.8 mg, 0.2 mmol) and trimethyl(vinyl)silane (87.9 μL, 0.6 mmol) were used. Purification via flash column chromatography on silica gel (petroleum ether) afforded **2m** as white solid (21.2 mg, 46% yield). ^{1}HNMR ($CDCl_3$, 400 MHz): δ (ppm) 7.91 – 7.87 (m, 3H), 7.82 (d, J = 1.2 Hz, 1H), 7.71 – 7.69 (m, 1H), 7.55 – 7.49 (m, 3H), 7.42 – 7.35 (m, 3H), 6.75 (dd, J = 17.6, 11.2 Hz, 1H), 5.75 (dd, J = 17.6, 1.2 Hz, 1H), 5.20 (dd, J = 11.2, 1.2 Hz, 1H). ^{13}C NMR ($CDCl_3$, 100 MHz): δ (ppm) 140.9, 138.5, 136.1, 136.0, 133.3, 132.5, 130.5, 128.7, 128.4, 128.2, 127.9, 127.83, 127.76, 127.7, 126.4, 126.2, 125.9, 115.0. HRMS (ESI^{+}): calcd for $C_{18}H_{14}Na^{+}$ $[M+Na]^{+}$, 253.0988; found 253.0985.

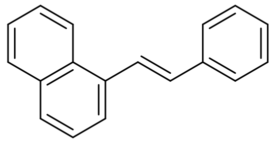

(*E*)-1-Styrylnaphthalene (3a)

Following the general procedure I. 1-Nitronaphthalene (103.8 mg, 0.6 mmol) and styrene (23.0 μL, 0.2 mmol) were used. Purification via flash column chromatography on silica gel (petroleum ether) afforded **3a** as a white solid (42.8 mg, 93% yield, *E*/*Z* > 99:1). ^{1}H NMR (DMSO-d_6, 400 MHz): δ (ppm) 8.41 (d, J = 8.0 Hz, 1H), 8.08 (d, J = 16.0 Hz, 1H), 7.97 – 7.94 (m, 1H), 7.88 (d, J = 7.6 Hz, 2H), 7.78 – 7.76 (m, 2H), 7.61 – 7.53 (m, 3H), 7.42 (t, J = 7.6 Hz, 2H), 7.33 – 7.28 (m, 2H). ^{13}C NMR (DMSO-d_6, 100 MHz): δ (ppm) 137.2, 134.3, 133.4, 131.4, 130.8, 128.7, 128.5, 127.9, 127.8, 126.9, 126.3, 126.0, 125.8, 125.0, 123.9, 123.2. HRMS (ESI$^+$): calcd for $C_{18}H_{15}^+$ [M+H]$^+$, 231.1168; found 231.1161.

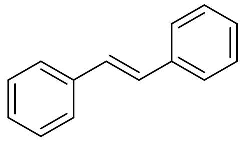

(*E*)-1,2-Diphenylethene (3b)

Following the general procedure I. Nitrobenzene (61.2 μL, 0.6 mmol) and styrene (23.0 μL, 0.2 mmol) were used. Purification via flash column chromatography on silica gel (petroleum ether) afforded **3b** as a white solid (22.0 mg, 61% yield, *E*/*Z* > 99:1). ^{1}H NMR ($CDCl_3$, 400 MHz): δ (ppm) 7.54 – 7.51 (m, 4H), 7.39 – 7.34 (m, 4H), 7.29 – 7.24 (m, 2H),7.12 (s, 2H). ^{13}C NMR ($CDCl_3$, 100 MHz): δ (ppm) 137.4, 128.82, 128.80, 127.76, 126.6. HRMS (ESI$^+$): calcd for $C_{14}H_{12}Na^+$ [M+Na]$^+$, 203.0831; found 203.0833.

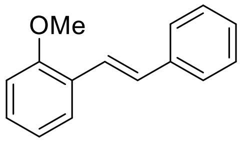

(*E*)-1-Methoxy-2-styrylbenzene (3c)

Following the general procedure I. 1-Methoxy-2-nitrobenzene (72.9 μL, 0.6 mmol) and styrene (23.0 μL, 0.2 mmol) were used. Purification via flash column chromatography on silica gel (petroleum ether/ethyl acetate = 20:1, v/v) afforded **3c** as a white solid (26.5 mg, 63% yield, *E*/*Z* > 99:1). ^{1}H NMR (DMSO-d_6, 400 MHz): δ (ppm) 7.66 (dd, J = 8.0 Hz, 1H), 7.56 (d, J = 8.0 Hz, 2H), 7.42 (d, J = 16.0 Hz, 1H), 7.37 (t, J = 7.6 Hz, 2H), 7.30 – 7.20 (m, 3H), 7.04 (d, J = 8.0 Hz, 1H), 6.97 (t, J = 7.6 Hz, 1H). ^{13}C NMR (DMSO-d_6, 100 MHz): δ (ppm) 156.5, 137.5, 129.0, 128.8, 128.7, 127.5, 126.34, 126.32, 125.4, 122.9, 120.6, 111.4, 55.5. HRMS (ESI$^+$): calcd for $C_{15}H_{15}O^+$ [M+H]$^+$, 211.1117; found 211.1115.

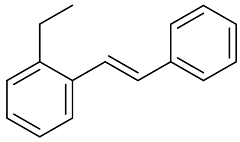

(*E*)-1-Ethyl-2-styrylbenzene (3d)

Following the general procedure I. 1-Ethyl-2-nitrobenzene (80.5 μL, 0.6 mmol) and styrene (23.0 μL, 0.2 mmol) were used. Purification via flash column chromatography on silica gel (petroleum ether) afforded **3d** as a white solid (33.8 mg, 81% yield, *E*/*Z* = 95:5). ^{1}H NMR (DMSO-d_6, 400 MHz): δ (ppm) 7.67 – 7.65 (m, 1H), 7.63 (d, J = 8.0 Hz, 2H), 7.45 (d, J = 16.0 Hz, 1H), 7.38 (t, J = 7.6 Hz, 2H), 7.28 (t, J = 7.2 Hz, 1H), 7.23 – 7.21 (m, 3H), 7.12 (d, J = 16.4 Hz, 1H), 2.78 (q, J = 7.6 Hz, 2H), 1.16 (t, J = 7.6 Hz, 3H). ^{13}C NMR (DMSO-d_6, 100 MHz): δ (ppm) 141.6, 137.3, 135.1, 129.8, 128.9, 128.7, 127.8, 127.6, 126.6, 126.2, 125.6, 125.4, 25.8, 15.7. HRMS (ESI$^+$): calcd for $C_{16}H_{17}^+$ [M+H]$^+$, 209.1325; found 209.1324.

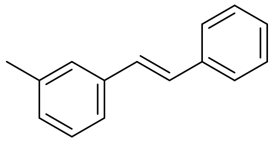

(*E*)-1-Methyl-3-styrylbenzene (3e)

Following the general procedure I. 1-Methyl-3-nitrobenzene (71.1 μL, 0.6 mmol) and styrene (23.0 μL, 0.2 mmol) were used. Purification via flash column chromatography on silica gel (petroleum ether) afforded **3e** as a white solid (26.8 mg, 69% yield, *E*/*Z* = 96:4). ^{1}H NMR (DMSO-d_6, 400 MHz): δ (ppm) 7.61 – 7.58 (m, 2H), 7.43 (s, 1H), 7.40 – 7.35 (m, 3H), 7.29 – 7.24 (m, 2H), 7.23 (d, J = 2.4 Hz, 2H), 7.09 (d, J = 8.0 Hz, 1H), 2.33 (s, 3H). ^{13}C NMR (DMSO-d_6, 100 MHz): δ (ppm) 137.8, 137.1, 136.9, 128.7, 128.6, 128.5, 128.4, 128.2, 127.6, 127.0, 126.4, 123.8, 21.0. HRMS (ESI$^+$): calcd for $C_{15}H_{15}^+$ [M+H]$^+$, 195.1168; found 195.1164.

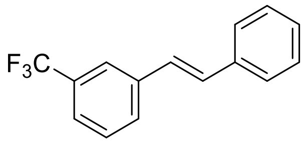

(*E*)-1-Styryl-3-(trifluoromethyl)benzene (3f)

Following the general procedure I. 1-Nitro-3-(trifluoromethyl)benzene (79.9 μL, 0.6 mmol) and styrene (23.0 μL, 0.2 mmol) were used. Purification via flash column chromatography on silica gel (petroleum ether) afforded **3f** as a white solid (16.0 mg, 65% yield, *E*/*Z* = 94:6). ^{1}H NMR (DMSO-d_6, 400 MHz): δ (ppm) 7.96 (s, 1H), 7.94 – 7.91 (m, 1H), 7.65 – 7.61 (m, 4H), 7.45 (d, J = 16.4 Hz, 1H), 7.42 – 7.39 (m, 2H), 7.37 (d, J = 13.6 Hz, 1H), 7.33 – 7.28 (m, 1H). ^{13}C NMR (DMSO-d_6, 100 MHz): δ (ppm) 138.3, 136.6, 130.5, 130.2 (q, $J_{C\text{-}F}$ = 1.1 Hz), 129.7, 129.6 (q, $J_{C\text{-}F}$ = 31.0 Hz), 128.8,

128.1, 126.8, 126.7, 124.3 (q, J_{C-F} = 264.0 Hz), 123.9 (q, J_{C-F} = 3.9 Hz), 122.9 (q, J_{C-F} = 3.7 Hz). HRMS (ESI$^+$): calcd for $C_{15}H_{12}F_3^+$ [M+H]$^+$, 249.0886; found 249.0882.

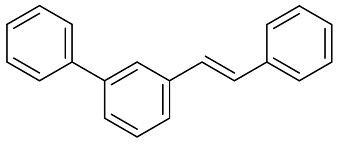

(*E*)-3-Styryl-1,1'-biphenyl (3g)

Following the general procedure I. 3-Nitro-1,1'-biphenyl (119.4 mg, 0.6 mmol) and styrene (23.0 μL, 0.2 mmol) were used. Purification via flash column chromatography on silica gel (petroleum ether) afforded **3g** as a white solid (34.4 mg, 67% yield, *E*/*Z* > 99:1). ^{1}H NMR (DMSO-d_6, 400 MHz): δ (ppm) 7.90 (t, J = 1.6 Hz, 1H), 7.74 – 7.71 (m, 2H), 7.65– 7.60 (m, 3H), 7.59 – 7.56 (m, 1H), 7.52 – 7.46 (m, 3H), 7.42 – 7.38 (m, 4H), 7.34 (d, J = 16.4 Hz, 1H), 7.31 – 7.26 (m, 1H). ^{13}C NMR (DMSO-d_6, 100 MHz): δ (ppm) 140.6, 140.0, 137.7, 137.0, 129.3, 128.91, 128.86, 128.7, 128.3, 127.7, 127.6, 126.8, 126.5, 126.0, 125.6, 124.8. HRMS (ESI$^+$): calcd for $C_{20}H_{17}^+$ [M+H]$^+$, 257.1325; found 257.1330.

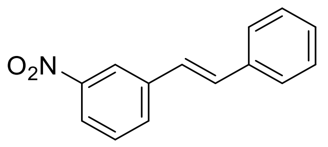

(*E*)-1-Nitro-3-styrylbenzene (3h)

Following the general procedure I. 1,3-Dinitrobenzene (100.8 mg, 0.6 mmol) and styrene (23.0 μL, 0.2 mmol) were used. Purification via flash column chromatography on silica gel (petroleum ether/ethyl acetate = 10:1, v/v) afforded **3h** as a light yellow solid (24.3 mg, 54% yield, *E*/*Z* > 99:1). ^{1}H NMR (DMSO-d_6, 400 MHz): δ (ppm) 8.44 (s, 1H), 8.10 (t, J = 9.6 Hz, 2H), 7.67 (t, J = 8.0 Hz, 3H), 7.51 (d, J = 16.4 Hz, 1H), 7.45 – 7.40 (m, 3H), 7.32 (t, J = 7.6 Hz, 1H). ^{13}C NMR (DMSO-d_6, 100 MHz): δ (ppm) 148.4, 139.1, 136.5, 132.6, 131.4, 130.2, 128.8, 128.4, 126.9, 126.3, 122.0, 120.8. HRMS (ESI$^+$): calcd for $C_{14}H_{11}NO_2Na^+$ [M+Na]$^+$, 248.0682; found 248.0689.

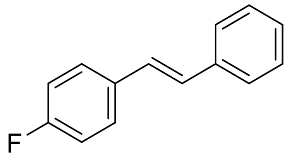

(*E*)-1-Fluoro-4-styrylbenzene (3i)

Following the general procedure I. 1-Fluoro-4-nitrobenzene (84.6 mg, 0.6 mmol) and styrene (23.0 μL, 0.2 mmol) were used. Purification via flash column chromatography on silica gel (petroleum ether/ethyl acetate = 20:1, v/v) afforded **3i** as a white solid (25.1 mg, 63% yield, *E*/*Z* > 99:1). ^{1}H NMR (DMSO-d_6, 400 MHz): δ (ppm) 7.67 – 7.64 (m, 2H), 7.59 (dd, J = 8.0, 1.2 Hz, 2H), 7.38 (t, J

= 7.6 Hz, 2H), 7.28 (d, J = 7.6 Hz, 1H), 7.25 – 7.18 (m, 4H). ^{13}C NMR (DMSO-d_6, 100 MHz): δ (ppm) 161.7 (d, $J_{C\text{-}F}$ = 245 Hz), 137.0, 133.6 (d, $J_{C\text{-}F}$ = 3.1 Hz), 128.7, 128.37 (d, $J_{C\text{-}F}$ = 8.2 Hz), 128.34, 127.7, 127.2, 126.5, 115.6 (d, $J_{C\text{-}F}$ = 21.5 Hz). HRMS (ESI$^+$): calcd for $C_{14}H_{12}F^+$ [M+H]$^+$, 1999.0918; found 199.0919.

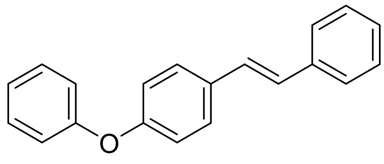

(*E*)-1-Phenoxy-4-styrylbenzene (3j)

Following the general procedure I. 1-Nitro-4-phenoxybenzene (129.0 mg, 0.6 mmol) and styrene (23.0 μL, 0.2 mmol) were used. Purification via flash column chromatography on silica gel (petroleum ether/ethyl acetate = 10:1, v/v) afforded **3j** as a white solid (27.5 mg, 51% yield, *E*/*Z* > 99:1). ^{1}HNMR (DMSO-d_6, 400 MHz): δ (ppm) 7.65 – 7.61 (m, 2H), 7.60 – 7.57 (m, 2H), 7.44 – 7.35 (m, 4H), 7.28 – 7.25 (m, 1H), 7.21 (d, J = 17.6 Hz, 2H), 7.18 – 7.14 (m, 1H), 7.06 – 6.99 (m, 4H). ^{13}C NMR (DMSO-d_6, 100 MHz): δ (ppm) 156.5, 156.3, 137.1, 132.4, 130.1, 128.7, 128.1, 127.63, 127.57, 127.5, 126.4, 123.6, 118.8, 118.7. HRMS (ESI$^+$): calcd for $C_{20}H_{17}O^+$ [M+H]$^+$ 273.1274; found 273.1269.

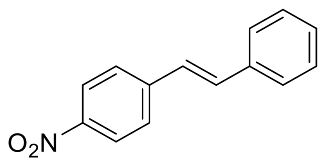

(*E*)-1-Nitro-4-styrylbenzene (3k)

Following the general procedure III. 1,4-Dinitrobenzene (100.8 mg, 0.6 mmol) and styrene (23.0 μL, 0.2 mmol) were used. Purification via flash column chromatography on silica gel (petroleum ether/ethyl acetate = 10:1, v/v) afforded **3k** as a white solid (18.9 mg, 42% yield, *E*/*Z* = 97:3). ^{1}H NMR (DMSO-d_6, 400 MHz): δ (ppm) 8.23 (d, J = 8.8 Hz, 2H), 7.87 (d, J = 8.8 Hz, 2H), 7.68 (d, J = 7.6 Hz, 2H), 7.54 (d, J = 16.4 Hz, 1H), 7.45 – 7.40 (m, 3H), 7.34 (t, J = 7.2 Hz, 1H). ^{13}C NMR (DMSO-d_6, 100 MHz): δ (ppm) 146.2, 144.0, 136.3, 133.3, 128.9, 128.7, 127.3, 127.2, 126.4, 124.1. HRMS (ESI$^+$): calcd for $C_{14}H_{11}NO_2Na^+$ [M+Na]$^+$, 248.0682; found 248.0690.

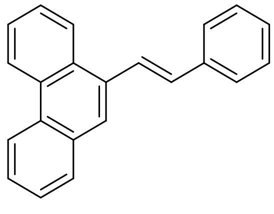

(*E*)-9-Styrylphenanthrene (3l)

Following the general procedure I. 9-Nitrophenanthrene (133.8 mg, 0.6 mmol) and styrene (23.0 μL, 0.2 mmol) were used. Purification via flash column chromatography on silica gel (petroleum ether/ethyl acetate = 20:1, v/v) afforded **3l** as a white solid (37.0 mg, 66% yield, *E*/*Z* = 93:7). ^{1}H NMR (DMSO-d_6, 400 MHz): δ (ppm) 8.91 (dd, *J* = 7.2, 2.1 Hz, 1H), 8.84 – 8.82 (m, 1H), 8.46 – 8.44 (m, 1H), 8.19 (s, 1H), 8.10 (d, *J* = 16.0 Hz, 1H), 8.06 – 8.04 (m, 1H), 7.81 (d, *J* = 7.2 Hz, 2H), 7.77 – 7.64 (m, 4H), 7.44 (d, *J* = 7.6 Hz, 2H), 7.38 (d, *J* = 16.0 Hz, 1H), 7.35 – 7.31 (m, 1H). ^{13}C NMR (DMSO-d_6, 100 MHz): δ (ppm) 137.2, 133.2, 131.9, 131.4, 130.1, 130.0, 129.6, 128.74, 128.66, 127.9, 127.1, 127.0, 126.94, 126.86, 126.84, 125.5, 124.7, 123.8, 123.4, 122.8. HRMS (ESI$^+$): calcd for $C_{22}H_{17}^+$ [M+H]$^+$, 281.1325; found 281.1330.

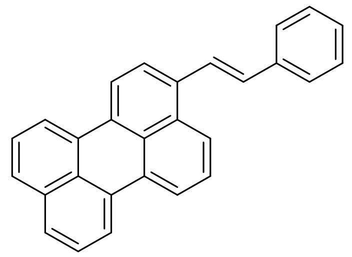

(*E*)-3-Styrylperylene (3m)

Following the general procedure I. 3-Nitroperylene (59.4 mg, 0.2 mmol) and styrene (69.0 μL, 0.6 mmol) were used. Purification via flash column chromatography on silica gel (petroleum ether/dichloromethane = 10:1, v/v) afforded **3m** as a yellow solid (29.8 mg, 42% yield, *E*/*Z* > 99:1). ^{1}H NMR (DMSO-d_6, 400 MHz): δ (ppm) 8.43 (d, *J* = 7.6 Hz, 1H), 8.40 – 8.37 (m, 3H), 8.32 (d, *J* = 8.4 Hz, 1H), 8.03 (d, *J* = 16.0 Hz, 1H), 7.93 (d, *J* = 8.0 Hz, 1H), 7.80 (d, *J* = 7.6 Hz, 4H), 7.62 (t, *J* = 8.0 Hz, 1H), 7.56 (t, *J* = 8.0 Hz, 2H), 7.43 (t, *J* = 7.6 Hz, 2H), 7.37 (d, *J* = 16.0 Hz, 1H), 7.32 (t, *J* = 7.6 Hz, 1H). ^{13}C NMR (DMSO-d_6, 100 MHz): δ (ppm) 137.3, 134.3, 134.1, 132.1, 131.4, 130.8, 130.6, 130.5, 130.1, 128.7, 128.3, 128.0, 127.90, 127.89, 127.0, 124.6, 124.6, 124.12, 124.06, 121.0, 120.91, 120.90, 120.85, 120.7, 120.0. HRMS (ESI$^+$): calcd for $C_{28}H_{18}Na^+$ [M+Na]$^+$, 377.1301; found 377.1302.

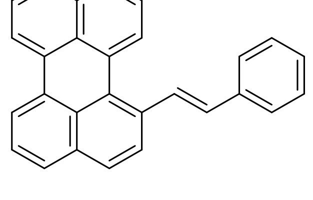

(*E*)-1-Styrylperylene (3n)

Following the general procedure I. 1-Nitroperylene (59.4 mg, 0.2 mmol) and styrene (69.0 μL, 0.6 mmol) were used. Purification via flash column chromatography on silica gel (petroleum ether/dichloromethane = 10:1, v/v) afforded **3n** as a yellow solid (63.0 mg, 89% yield, *E*/*Z* = 97:3). ^{1}H NMR (DMSO-d_6, 400 MHz): δ (ppm) 8.44 – 8.33 (m, 2H), 7.93 – 7.81 (m, 4H), 7.73 (t, *J* = 7.2 Hz, 1H), 7.68 – 7.60 (m, 4H), 7.58 – 7.42 (m, 4H), 7.32 (t, *J* = 7.2 Hz, 1H), 7.22 – 7.14 (m, 2H). ^{13}C NMR (DMSO-d_6, 100 MHz): δ (ppm) 137.4, 133.8, 133.3, 133.1, 130.9, 130.4, 130.3, 130.1, 128.9,

128.82, 128.80, 128.5, 128.4, 128.0, 127.8, 127.74, 127.72, 127.60, 127.56, 127.5, 126.9, 126.7, 126.6, 126.4, 121.5, 120.8. HRMS (ESI$^+$): calcd for $C_{28}H_{18}Na^+$ [M+Na]$^+$, 377.1301; found 377.1307.

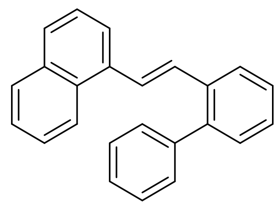

(*E*)-1-(2-([1,1'-Biphenyl]-2-yl)vinyl)naphthalene (4a)

Following the general procedure I. 1-Nitronaphthalene (103.8 mg, 0.6 mmol) and 2-vinyl-1,1'-biphenyl (69.0 μL, 0.2 mmol) were used. Purification via flash column chromatography on silica gel (petroleum ether/ dichloromethane = 20:1, v/v) afforded **4a** as a white solid (31.8 mg, 52% yield, *E*/*Z* = 96:4). ^{1}H NMR (DMSO-d_6, 400 MHz): δ (ppm) 8.32 (d, J = 9.2 Hz, 1H), 8.07 (d, J = 7.6 Hz, 1H), 7.96 (d, J = 16.0 Hz, 1H), 7.92 (dd, J = 6.8, 2.4 Hz, 1H), 7.83 (d, J = 8.0 Hz, 1H), 7.58 – 7.53 (m, 2H), 7.51 – 7.37 (m, 9H), 7.34 (dd, J = 7.6, 1.6 Hz, 1H), 7.08 (d, J = 16.0 Hz, 1H). ^{13}C NMR (DMSO-d_6, 100 MHz): δ (ppm) 140.7, 140.4, 134.8, 134.7, 133.4, 130.8, 130.2, 130.0, 129.6, 128.5, 128.4, 128.0, 127.9, 127.8, 127.3, 126.5, 126.33, 126.31, 126.1, 125.8, 123.9, 123.1. HRMS (ESI$^+$): calcd for $C_{24}H_{18}Na^+$ [M+Na]$^+$, 329.1301; found 329.1300.

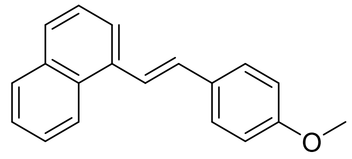

(*E*)-1-(4-Methoxystyryl)naphthalene (4b)

Following the general procedure I. 1-Nitronaphthalene (103.8 mg, 0.6 mmol) and 1-methoxy-4-vinylbenzene (26.6 μL, 0.2 mmol) were used. Purification via flash column chromatography on silica gel (petroleum ether/ dichloromethane = 8:1, v/v) afforded **4b** as a white solid (46.0 mg, 88% yield, *E*/*Z* = 94:6). ^{1}H NMR (DMSO-d_6, 400 MHz): δ (ppm) 8.40 (d, J = 7.6 Hz, 1H), 7.95 – 7.90 (m, 2H), 7.86 – 7.84 (m, 2H), 7.71 (d, J = 11.6 Hz, 2H), 7.60 – 7.51 (m, 3H), 7.24 (d, J = 16.0 Hz, 1H), 6.98 (d, J = 8.8 Hz, 2H), 3.80 (s, 3H). ^{13}C NMR (DMSO-d_6, 100 MHz): δ (ppm) 159.1, 134.6, 133.4, 131.0, 130.8, 123.0, 128.5, 128.3, 127.5, 126.1, 125.9, 125.8, 123.9, 122.7, 122.5, 114.2, 55.2. HRMS (ESI$^+$): calcd for $C_{19}H_{17}O^+$ [M+H]$^+$, 261.1274; found 261.1275.

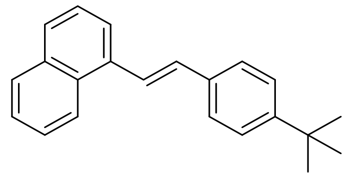

(*E*)-1-(4-(*tert*-Butyl)styryl)naphthalene (4c)

Following the general procedure I. 1-Nitronaphthalene (103.8 mg, 0.6 mmol) and 1-(tert-butyl)-4-vinylbenzene (36.6 μL, 0.2 mmol) were used. Purification via flash column chromatography on silica gel (petroleum ether/ dichloromethane = 20:1, v/v) afforded **4c** as a white solid (53.8 mg, 94% yield, *E*/*Z* = 95:5). ^{1}H NMR (DMSO-d_6, 400 MHz): δ (ppm) 8.39 (d, J = 8.0 Hz, 1H), 8.01 (d, J = 16.0 Hz, 1H), 7.96 – 7.94 (m, 1H), 7.87 (d, J = 7.3 Hz, 2H), 7.69 (d, J = 8.4 Hz, 2H), 7.61 – 7.52 (m, 3H), 7.43 (d, J = 8.4 Hz, 2H), 7.27 (d, J = 16.0 Hz, 1H), 1.31 (s, 9H). ^{13}C NMR (DMSO-d_6, 100 MHz): δ (ppm) 150.5, 134.5, 134.4, 133.4, 131.2, 130.8, 128.5, 127.8, 126.7, 126.2, 126.0, 125.8, 125.5, 124.1, 123.8, 123.0, 34.4, 31.1. HRMS (ESI$^+$): calcd for $C_{22}H_{23}^+$ [M+H]$^+$, 287.1794; found 287.1792.

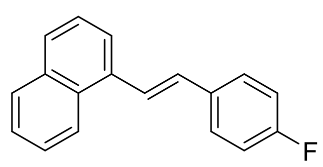

(*E*)-1-(4-Fluorostyryl)naphthalene (4d)

Following the general procedure I. 1-Nitronaphthalene (103.8 mg, 0.6 mmol) and 1-(tert-butyl)-4-vinylbenzene (23.9 μL, 0.2 mmol) were used. Purification via flash column chromatography on silica gel (petroleum ether/ dichloromethane = 20:1, v/v) afforded **4d** as a white solid (26.8 mg, 54% yield, *E*/*Z* > 99:1). ^{1}H NMR (DMSO-d_6, 400 MHz): δ (ppm) 8.42 (d, J = 8.0 Hz, 1H), 8.04 (d, J = 16.0 Hz, 1H), 7.96 – 7.94 (m, 1H), 7.89 – 7.86 (m, 2H), 7.85 – 7.81 (m, 2H), 7.60 – 7.52 (m, 3H), 7.32 – 7.23 (m, 3H). ^{13}C NMR (DMSO-d_6, 100 MHz): δ (ppm) 161.8 (d, $J_{C\text{-}F}$ = 244.9 Hz), 134.2, 133.9 (d, $J_{C\text{-}F}$ = 3.2 Hz), 133.4, 130.8, 130.2, 128.8 (d, $J_{C\text{-}F}$ = 8.0 Hz), 128.5, 127.9, 126.3, 126.0, 125.8, 124. 9 (d, $J_{C\text{-}F}$ = 2.4 Hz), 123.9, 123.1, 115.6 (d, $J_{C\text{-}F}$ = 21.4 Hz). HRMS (ESI$^+$): calcd for $C_{18}H_{14}F^+$ [M+H]$^+$, 249.1074; found 249.1076.

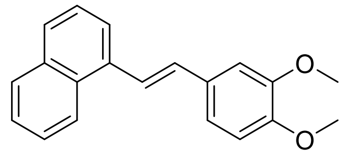

(*E*)-1-(3,4-Dimethoxystyryl)naphthalene (4e)

Following the general procedure I. 1-Nitronaphthalene (103.8 mg, 0.6 mmol) and 1,2-dimethoxy-4-vinylbenzene (29.6 μL, 0.2 mmol) were used. Purification via flash column chromatography on silica gel (petroleum ether/ dichloromethane = 8:1, v/v) afforded **4e** as a brown oil (46.4 mg, 80% yield, *E*/*Z* = 96:4). ^{1}H NMR (DMSO-d_6, 400 MHz): δ (ppm) 8.44 (d, J = 8.0 Hz, 1H), 7.98 – 7.93 (m, 2H), 7.86 – 7.83 (m, 2H), 7.60 – 7.51 (m, 3H), 7.44 (s, 1H), 7.24 – 7.20 (m, 2H), 6.98 (d, J = 8.4 Hz, 1H), 3.87 (s, 3H), 3.79 (s, 3H). ^{13}C NMR (DMSO-d_6, 100 MHz): δ (ppm) 149.0, 148.9, 134.7, 133.5, 131.5, 130.8, 130.2, 128.4, 127.5, 126.1, 125.93, 125.87, 124.0, 122.7, 122.7, 120.6, 111.7, 109.5, 55.7, 55.5. HRMS (ESI$^+$): calcd for $C_{20}H_{18}O_2Na^+$ [M+Na]$^+$, 313.1199 found 313.1201.

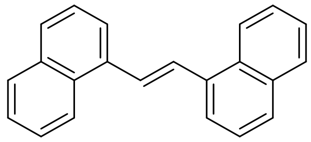

(*E*)-1,2-Di(naphthalen-1-yl)ethene (4f)

Following the general procedure I. 1-Nitronaphthalene (103.8 mg, 0.6 mmol) and 1-vinylnaphthalene (29.7 μL, 0.2 mmol) were used. Purification via flash column chromatography on silica gel (petroleum ether/ dichloromethane = 20:1, v/v) afforded **4f** as a white solid (36.4 mg, 65% yield, *E*/*Z* = 97:3). ^{1}H NMR (DMSO-d_6, 400 MHz): δ (ppm) 8.43 (d, *J* = 7.8 Hz, 2H), 8.11– 8.10 (m, 4H), 7.98 (d, *J* = 7.2 Hz, 2H), 7.93 (d, *J* = 8.0 Hz, 2H), 7.62 – 7.55 (m, 6H). ^{13}C NMR (DMSO-d_6, 100 MHz): δ (ppm) 134.4, 133.4, 130.9, 128.5, 128.1, 127.9, 126.4, 126.0, 125.9, 123.80, 123.81. HRMS (ESI$^+$): calcd for $C_{22}H_{17}^+$ [M+H]$^+$, 281.1325 found 281.1330.

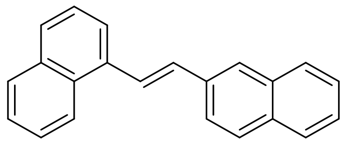

(*E*)-1-(2-(Naphthalen-2-yl)vinyl)naphthalene (4g)

Following the general procedure I. 1-Nitronaphthalene (103.8 mg, 0.6 mmol) and 2-vinylnaphthalene (27.8 μL, 0.2 mmol) were used. Purification via flash column chromatography on silica gel (petroleum ether/ dichloromethane = 20:1, v/v) afforded **4g** as a white solid (23.1 mg, 41% yield, *E*/*Z* > 99:1). ^{1}H NMR (DMSO-d_6, 400 MHz): δ (ppm) 8.50 (d, *J* = 7.6 Hz, 1H), 8.24 (d, *J* = 16.0Hz, 1H), 8.16 (s, 1H), 8.11 (dd, *J* = 8.6, 1.6Hz, 1H), 7.98 – 7.90 (m, 6H), 7.64 – 7.46 (m, 6H). ^{13}C NMR (DMSO-d_6, 100 MHz): δ (ppm) 134.9, 134.3, 133.5, 133.3, 132.7, 131.4, 130.9, 128.5, 128.2, 128.0, 127.97, 127.7, 126.8, 126.5, 126.3, 126.1, 126.0, 125.9, 125.4, 124.2, 123.9, 123.2. HRMS (ESI$^+$): calcd for $C_{22}H_{17}^+$ [M+H]$^+$, 281.1325 found 281.1327.

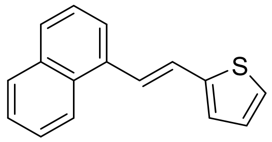

(*E*)-2-(2-(Naphthalen-1-yl)vinyl)thiophene (4h)

Following the general procedure I. 1-Nitronaphthalene (103.8 mg, 0.6 mmol) and 2-vinylnaphthalene (27.8 μL, 0.2 mmol) were used. Purification via flash column chromatography on silica gel (petroleum ether/ dichloromethane = 20:1, v/v) afforded **4h** as a white solid (25.5 mg, 54% yield, *E*/*Z* = 97:3). ^{1}H NMR (DMSO-d_6, 400 MHz): δ (ppm) 8.29 (d, *J* = 8.3 Hz, 1H), 7.95 (d, *J* = 7.6 Hz, 1H), 7.87 (t, *J* = 7.2 Hz, 2H), 7.75 (d, *J* = 16.0 Hz, 1H), 7.61 – 7.48 (m, 5H), 7.37 (d, *J* = 3.6 Hz, 1H), 7.11 (dd, *J* = 5.2, 3.6 Hz, 1H). ^{13}C NMR (DMSO-d_6, 100 MHz): δ (ppm) 142.4, 133.6, 133.4, 130.5, 128.6 128.1 128.0, 127.1, 126.4, 126.0, 125.9, 125.7, 124.6, 123.9, 123.5, 123.1. HRMS (ESI$^+$): calcd for $C_{16}H_{13}S^+$ [M+H]$^+$, 237.0732 found 237.0727.

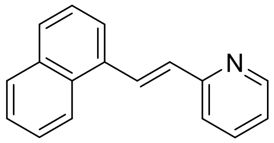

(*E*)-2-(2-(Naphthalen-1-yl)vinyl)pyridine (4i)

Following the general procedure I. 1-Nitronaphthalene (103.8 mg, 0.6 mmol) and 2-vinylpyridine (21.6 μL, 0.2 mmol) were used. Purification via flash column chromatography on silica gel (petroleum ether/ dichloromethane = 8:1, v/v) afforded **4i** as a brown solid (38.1 mg, 82% yield, *E*/*Z* > 99:1). ^{1}H NMR (DMSO-d_6, 400 MHz): δ (ppm) 8.64 (d, J = 4.4 Hz, 1H), 8.50 (d, J = 16.0 Hz, 1H), 8.33 (d, J = 8.4 Hz, 1H), 7.99 – 7.93 (m, 3H), 7.83 (t, J = 7.6 Hz, 1H), 7.72 (d, J = 7.6 Hz, 1H), 7.64 – 7.55 (m, 3H), 7.38 (d, J = 16.0 Hz, 1H), 7.32 – 7.29 (m, 1H). ^{13}C NMR (DMSO-d_6, 100 MHz): δ (ppm) 155.0, 149.6, 136.9, 133.43, 133.40, 130.94, 130.89, 128.6, 128.3, 126.6, 126.1, 125.9, 123.7, 123.4, 122.7. HRMS (ESI$^+$): calcd for $C_{17}H_{14}N^+$ [M+H]$^+$, 232.1121; found 232.1120.

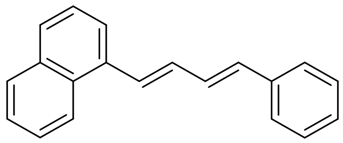

1-((1*E*,3*E*)-4-Phenylbuta-1,3-dien-1-yl)naphthalene (4j)

Following the general procedure I. 1-Nitronaphthalene (103.8 mg, 0.6 mmol) and (*E*)-buta-1,3-dien-1-ylbenzene (28.0 μL, 0.2 mmol) were used. Purification via flash column chromatography on silica gel (petroleum ether/ dichloromethane = 20:1, v/v) afforded **4j** as a light yellow solid (43.3 mg, 85% yield, *E*,*E*/other isomers > 99:1). ^{1}H NMR (DMSO-d_6, 400 MHz): δ (ppm) 8.29 (d, J = 8.4 Hz, 1H), 7.95 (dd, J = 8.0, 1.2 Hz, 1H), 7.86 (dd, J = 7.6, 3.2 Hz, 2H), 7.65 (d, J = 15.2 Hz, 1H), 7.62 – 7.51 (m, 5H), 7.38 (t, J = 7.6 Hz, 2H), 7.34 – 7.25 (m, 2H), 7.19 (dd, J = 15.2, 10.4 Hz, 1H), 6.82 (d, J = 15.6 Hz, 1H). ^{13}C NMR (DMSO-d_6, 100 MHz): δ (ppm) 137.1, 133.8, 133.5, 132.9, 131.9, 130.4, 129.9, 129.0, 128.9, 128.6, 127.9, 127.8, 126.4, 126.4, 126.0, 125.9, 123.3, 122.8. HRMS (ESI$^+$): calcd for $C_{20}H_{17}^+$ [M+H]$^+$, 257.1325; found 257.1321.

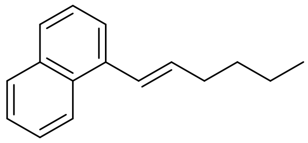

(*E*)-1-(Hex-1-en-1-yl)naphthalene (4k)

Following the general procedure I. 1-Nitronaphthalene (103.8 mg, 0.6 mmol) and hex-1-ene (24.8 μL, 0.2 mmol) were used. Purification via flash column chromatography on silica gel (petroleum ether/dichloromethane = 20:1, v/v) afforded **4k** as a white solid (43.3 mg, 62% yield, *E*/*Z* = 94:6). ^{1}H NMR (DMSO-d_6, 400 MHz): δ (ppm) 8.17 – 8.15 (m, 1H), 7.91 (dd, J = 7.6, 1.6 Hz, 1H), 7.81

(d, J = 8.0 Hz, 1H), 7.61 (d, J = 7.2 Hz, 1H), 7.56 – 7.49 (m, 2H), 7.46 (t, J = 8.0 Hz, 1H), 7.18 (d, J = 15.6 Hz, 1H), 6.28 (dt, J = 15.5, 7.0 Hz, 1H), 2.31 (qd, J = 7.2, 1.6 Hz, 2H), 1.53 – 1.46 (m, 2H), 1.43 – 1.34 (m, 2H), 0.93 (t, J = 7.2 Hz, 3H). ^{13}C NMR (DMSO-d_6, 100 MHz): δ (ppm) 134.8, 134.0, 133.3, 130.4, 128.4, 127.1, 126.4, 126.0, 125.7, 123.6, 123.1, 32.4, 31.0, 21.8, 13.8. HRMS (ESI$^+$): calcd for $C_{16}H_{18}Na^+$ [M+Na]$^+$, 233.1301; found 233.1301.

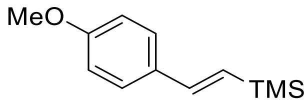

(*E*)-(4-Methoxystyryl)trimethylsilane (4l)

Following the general procedure I. 1-Methoxy-4-nitrobenzene (91.8 mg, 0.6 mmol) and trimethyl(vinyl)silane (29.3 μL, 0.2 mmol) were used. Purification via flash column chromatography on silica gel (petroleum ether/dichloromethane = 10:1, v/v) afforded **4l** as colorless oil (14.5 mg, 35% yield, E/Z > 99:1). ^{1}H NMR ($CDCl_3$, 400 MHz): δ (ppm) 7.40 – 7.36 (m, 2H), 6.88 – 6.85 (m, 2H), 6.81 (d, J = 19.2 Hz, 1H), 6.31 (d, J = 19.2 Hz, 1H), 3.81 (s, 3H), 0.14 (s, 9H). ^{13}C NMR ($CDCl_3$, 100 MHz): δ (ppm) 159.6, 143.1, 131.5, 127.7, 126.8, 114.0, 55.5, -0.9. HRMS (ESI$^+$): calcd for $C_{12}H_{18}OSiNa^+$ [M+Na]$^+$, 229.1019; found 229.1018.

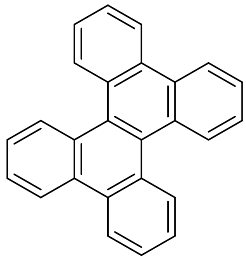

Dibenzo[*g,p*]chrysene (5a)

Following the general procedure IV. 2-Nitro-1,1'-biphenyl (39.8 mg, 0.2 mmol) and 2-vinyl-1,1'-biphenyl (54.0 mg, 0.3 mmol) were used. Purification via flash column chromatography on silica gel (petroleum ether/dichloromethane = 40:1, v/v) afforded **5a** as a pale yellow solid (19.3 mg, 29% yield). ^{1}H NMR ($CDCl_3$, 400 MHz): δ (ppm) 8.72 (dd, J = 3.6, 1.2 Hz, 4H), 8.70 (dd, J = 3.6, 1.2 Hz, 4H), 7.72 – 7.61 (m, 8H). ^{13}C NMR ($CDCl_3$, 100 MHz): δ (ppm) 131.0, 129.4, 129.0, 127.6, 126.7, 123.7. HRMS (ESI$^+$): calcd for $C_{26}H_{17}^+$ [M+H]$^+$, 329.1325; found 329.1320.

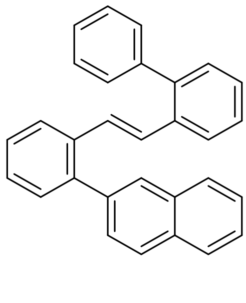

(*E*)-2-(2-(2-([1,1'-Biphenyl]-2-yl)vinyl)phenyl)naphthalene (5b')

Following the general procedure I. 2-Nitro-1,1'-biphenyl (39.8 mg, 0.2 mmol) and 2-(2-vinylphenyl)naphthalene (64.0 mg, 0.3 mmol) were used. Purification via flash column chromatography on silica gel (petroleum ether/dichloromethane = 40:1, v/v) afforded **5b'** as a white solid (31.3 mg, 41% yield). ^{1}H NMR ($CDCl_3$, 400 MHz): δ (ppm) 7.93 – 7.87 (m, 4H), 7.56 – 7.53 (m, 4H), 7.46 – 7.38 (m, 7H),7.34 – 7.32 (m, 2H), 7.30 – 7.28 (m, 2H), 7.25 – 7.20 (m, 1H), 7.10 (s, 2H). ^{13}C NMR ($CDCl_3$, 100 MHz): δ (ppm) 141.0, 140.94, 140.88, 138.5, 135.9, 135.6, 133.3, 132.4, 130.5, 130.2, 129.9, 128.7, 128.56, 128.4, 128.3, 128.2, 128.1, 127.70, 127.65, 127.6, 127.5, 127.43, 127.39, 127.1, 126.3, 126.1, 126.0, 125.9. HRMS (ESI$^+$): calcd for $C_{30}H_{22}Na^+$ [M+Na]$^+$, 405.1614; found 405.1611.

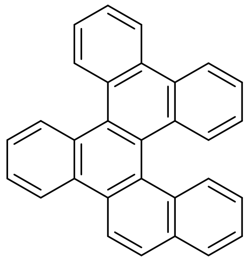

Tribenzo[*c,g,p*]chrysene (5b)

Following the general procedure IV. 2-Nitro-1,1'-biphenyl (39.8 mg, 0.2 mmol) and 2-(2-vinylphenyl)naphthalene (64.0 mg, 0.3 mmol) were used. Purification via flash column chromatography on silica gel (petroleum ether/dichloromethane = 40:1, v/v) afforded **5b** as a yellow solid (16.1 mg, 21% yield). ^{1}H NMR ($CDCl_3$, 400 MHz): δ (ppm) 8.83 (t, J = 7.6 Hz, 2H), 8.77 – 8.72 (m, 3H), 8.58 (d, J = 7.6 Hz, 1H), 8.26 (d, J = 8.8 Hz, 1H), 8.09 (d, J = 8.8 Hz, 1H), 8.01 (d, J = 8.4 Hz, 1H), 7.97 (d, J = 8.0 Hz, 1H), 7.72 – 7.63 (m, 4H), 7.57 – 7.53 (m, 1H), 7.50 – 7.46 (m, 1H), 7.29 – 7.20 (m, 2H). ^{13}C NMR ($CDCl_3$, 100 MHz): δ (ppm) 132.9, 131.6, 131.4, 130.9, 130.8, 123.0, 129.9, 129.8, 129.3, 129.2, 129.17, 129.0, 128.6, 128.2, 128.1, 127.0, 126.8, 126.73, 126.65, 126.6, 126.1, 125.9, 125.4, 125.0, 124.0, 123.8, 123.4, 121.1. HRMS (ESI$^+$): calcd for $C_{30}H_{18}Na^+$ [M+Na]$^+$, 401.1301; found 401.1306.

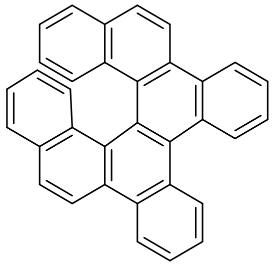

Dibenzo[*c,p*]naphtho[*1,2-g*]chrysene (5c)

Following the general procedure IV (The last intramolecular cyclodehydrogenation was conducted -10 °C). 1-Nitro-2-phenylnaphthalene (49.8 mg, 0.2 mmol) and 2-(2-vinylphenyl)naphthalene (64.0

mg, 0.3 mmol) were used. Purification via flash column chromatography on silica gel (petroleum ether/dichloromethane = 40:1, v/v) afforded **5c** as a yellow solid (9.4 mg, 11% yield). ^{1}H NMR ($CDCl_3$, 400 MHz): δ (ppm) 8.03 (d, J = 8.4 Hz, 2H), 7.96 (d, J = 8.4 Hz, 2H), 7.80 (d, J = 8.4 Hz, 2H), 7.69 – 7.60 (m, 5H), 7.53 (d, J = 8.4 Hz, 2H), 7.49 – 7.44 (m, 7H). ^{13}C NMR ($CDCl_3$, 100 MHz): δ (ppm) 136.7, 133.0, 131.6, 130.6, 130.0, 129.6, 129.1, 129.0, 128.8, 128.4, 128.3, 128.2, 128.0, 127.7, 127.6, 126.0, 124.6, 121.9. HRMS (ESI^{+}): calcd for $C_{34}H_{21}^{+}$ [M+H]$^{+}$, 429.1638; found 429.1636.

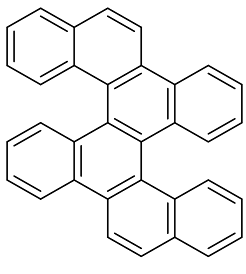

Tetrabenzo[*c,g,l,p*]chrysene (5d)

Following the general procedure IV. 2-(2-Nitrophenyl)naphthalene (49.8 mg, 0.2 mmol) and 2-(2-vinylphenyl)naphthalene (69.0 mg, 0.3 mmol) were used. Purification via flash column chromatography on silica gel (petroleum ether/dichloromethane = 40:1, v/v) afforded **5d** as a tawny solid (18.1 mg, 21% yield). ^{1}H NMR ($CDCl_3$, 400 MHz): δ (ppm) δ 8.77 (d, J = 9.2 Hz, 2H), 8.69 (d, J = 8.0 Hz, 2H), 8.43 (d, J = 8.8 Hz, 2H), 8.15 (dd, J = 8.4, 6.4 Hz, 4H), 8.01 (dd, J = 8.0, 1.2 Hz, 2H), 7.58 – 7.50 (m, 4H), 7.31 – 7.27 (m, 4H). ^{13}C NMR ($CDCl_3$, 100 MHz): δ (ppm) 132.9, 131.9, 131.0, 129.9, 129.4, 129.3, 129.1, 128.29, 128.25, 126.7, 126.2, 126.1, 125.41, 125.38, 125.36, 123.5, 121.2. HRMS (ESI^{+}): calcd for $C_{34}H_{21}^{+}$ [M+H]$^{+}$, 429.1638; found 429.1635.

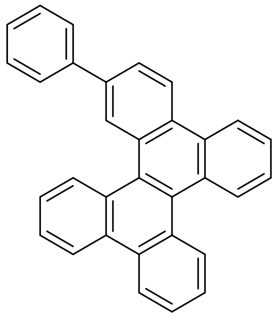

3-Phenyldibenzo[*g,p*]chrysene (5e)

Following the general procedure IV. 2'-Nitro-1,1':4',1''-terphenyl (55.0 mg, 0.2 mmol) and 2-vinyl-1,1'-biphenyl (54.0 mg, 0.3 mmol) were used. Purification via flash column chromatography on silica gel (petroleum ether/dichloromethane = 10:1, v/v) afforded **5e** as a light yellow solid (19.2 mg, 24% yield). ^{1}HNMR ($CDCl_3$, 400 MHz): δ (ppm) δ 8.92 (d, J = 2.0 Hz, 1H), 8.79 – 8.71 (m, 7H), 7.94 (dd, J = 8.4, 2.0 Hz, 1H), 7.68 – 7.63 (m, 8H), 7.50 (t, J = 7.6 Hz, 2H), 7.42 – 7.38 (m, 1H). ^{13}C NMR ($CDCl_3$, 100 MHz): δ (ppm) 141.2, 139.3, 131.01, 131.00, 130.8, 130.0, 129.7, 129.4, 129.3, 129.09, 129.08, 129.0, 128.9, 128.3, 128.01, 127.99, 127.7, 127.6, 127.5, 127.3, 126.9,

126.81, 126.79, 126.7, 126.0, 125.8, 124.3, 123.82, 123.76, 123.7. HRMS (ESI$^+$): calcd for $C_{32}H_{20}Na^+$ [M+Na]$^+$, 427.1457; found 427.1461

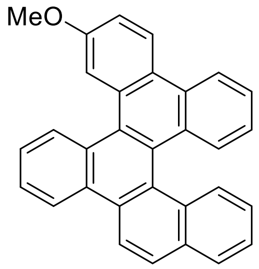

13-Methoxytribenzo[*c,g,p*]chrysene (5f)

Following the general procedure IV. 4'-Methoxy-2-nitro-1,1'-biphenyl (45.8 mg, 0.2 mmol) and 2-(2-vinylphenyl)naphthalene (69.0 mg, 0.3 mmol) were used. Purification via flash column chromatography on silica gel (petroleum ether/dichloromethane = 15:1, v/v) afforded **5e** as a yellow solid (12.2 mg, 15% yield). ^{1}HNMR ($CDCl_3$, 400 MHz): δ (ppm) 9.58 (d, J = 8.4 Hz, 1H), 9.11 – 9.05 (m, 4H), 8.80 (d, J = 8.8 Hz, 2H), 8.68 (d, J = 8.8 Hz, 2H), 7.97 (d, J = 8.4 Hz, 1H), 7.89 – 7.69 (m, 4H), 7.65 – 7.52 (m, 3H), 4.22 (s, 3H). ^{13}C NMR ($CDCl_3$, 100 MHz): δ (ppm) 131.30, 130.26, 130.0, 129.3, 129.0, 128.9, 128.7, 128.6, 128.3, 128.2, 127.8, 127.6, 127.4, 127.1, 126.7, 126.5, 126.3, 125.9, 125.6, 125.1, 124.3, 123.9, 123.5, 123.3, 123.0, 122.5, 117.8, 116.8, 113.1, 112.2, 56.4. HRMS (ESI$^+$): calcd for $C_{31}H_{21}O^+$ [M+H]$^+$, 409.1587; found 409.1591.

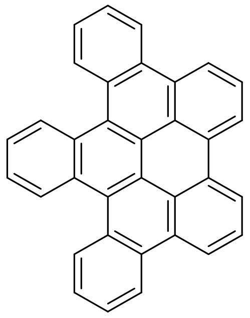

Dibenzo[*fg,ij*]naphtho[*1,2,3,4-rst*]pentaphene (5g)

Following the general procedure IV. 9-(2-Nitrophenyl)phenanthrene (59.8 mg, 0.2 mmol) and 2-vinyl-1,1'-biphenyl (54.0 mg, 0.3 mmol) were used. Purification via flash column chromatography on silica gel (petroleum ether/dichloromethane = 30:1, v/v) afforded **5g** as a yellow solid (17.2 mg, 20% yield). ^{1}H NMR ($CDCl_3$, 400 MHz): δ (ppm) 9.09 (dd, J = 6.4, 3.2 Hz, 2H), 8.95 – 8.93 (m, 2H), 8.78 (t, J = 7.2 Hz, 6H), 7.92 (t, J = 8.0 Hz, 2H), 7.7 – 7.67 (m, 6H). ^{13}C NMR (CDCl3, 100 MHz): δ (ppm) 131.3, 130.2, 123.0, 129.9, 129.6, 129.0, 128.5, 127.0, 126.9, 126.5, 125.8, 125.3, 124.6, 123.9, 122.2, 121.9, 121.8. HRMS (ESI$^+$): calcd for $C_{34}H_{19}^+$ [M+H]$^+$, 427.1481; found 427.1484.

V. Single crystal X-ray structures of 5d and 5g

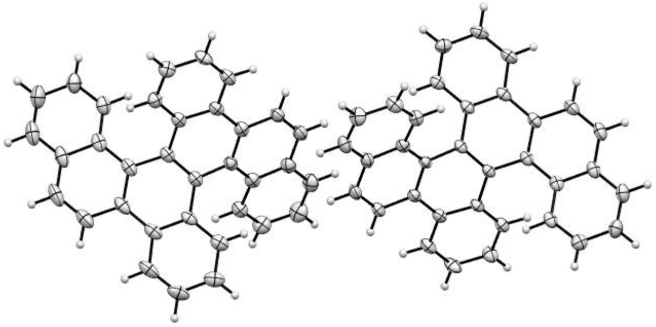

ORTEP diagram of **5d**

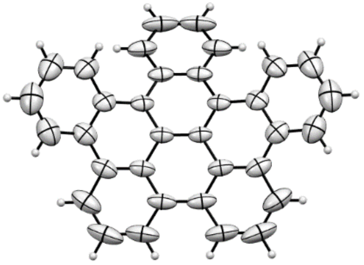

ORTEP diagram of **5g**

Fig. S2 ORTEP diagrams of 5d and 5g. Thermal ellipsoids are shown at the 50% probability level. CCDC 1975224 (**5d**), CCDC 1975225 (**5g**) contain the supplementary crystallographic data for this paper. These data can be obtained free of charge from The Cambridge Crystallographic Data Centre.

VI. Photophysical properties of double carbohelicene 5

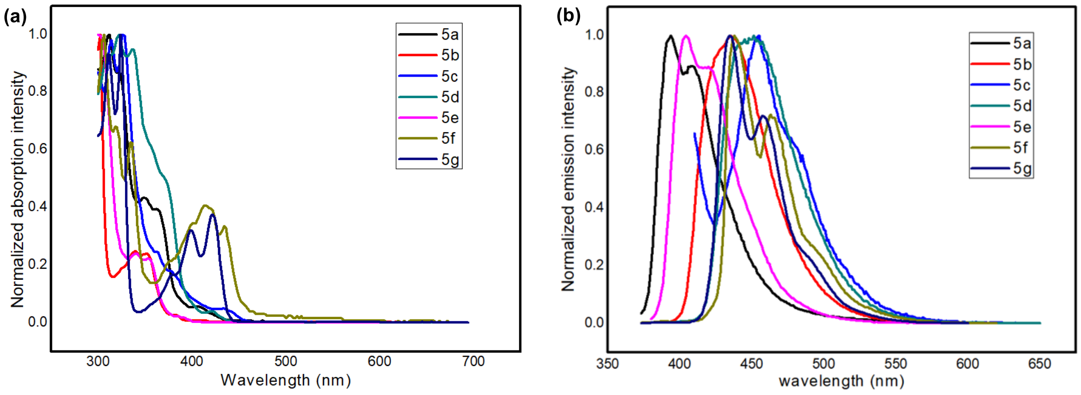

Fig. S3 UV-vis absorption (a) and fluorescence emission (b) of **5a-5g** in dichloromethane (5.0 μM)

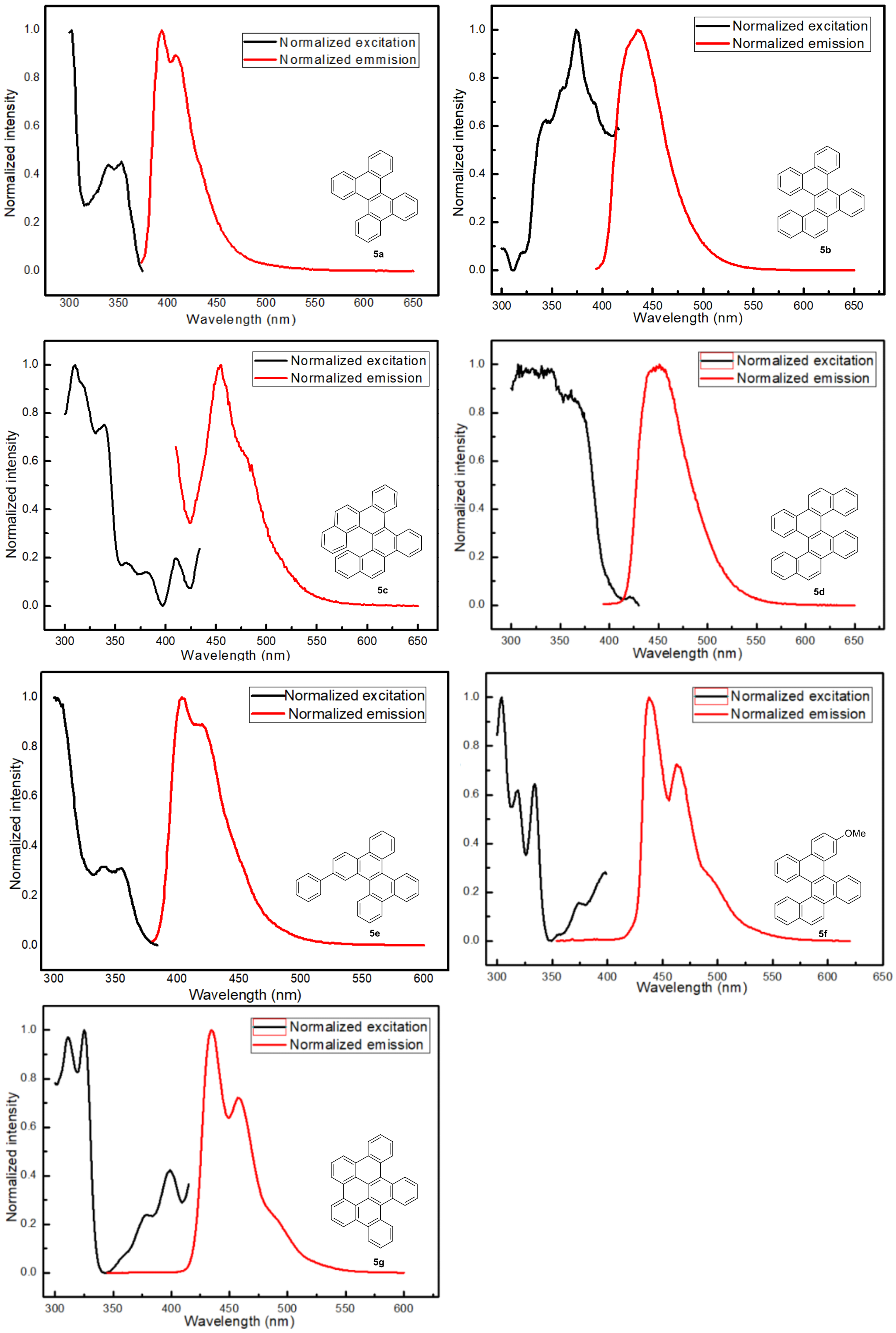

Fig. S4 Room temperature normalized excitation and emission spectra of double carbohelicene **5** in dichloromethane (5.0 μM).

Table S2 Photophysical data of double carbohelicene **5** in dichloromethane (5.0 μM)

Compound	λ_{abs} (nm)	λ_{ex} (nm)	λ_{em} (nm)	Stokes shift (cm^{-1})	Quantum yield (%)
**5a**	352	353	394	3028	9
**5b**	372	374	435	3893	12
**5c**	326	339	455	8696	5
**5d**	374	374	451	4565	5
**5e**	354	352	404	3657	23
**5f**	334	334	438	7109	24
**5g**	324	325	435	7875	32

VII. Computational study

The Gaussian 09 program was used for optimization (B3LYP/6-31G(d)). The **5d** was optimized without any symmetry assumptions. Zero-point energy, enthalpy, and Gibbs free energy at 298.15 K and 1 atm were estimated from the gas-phase studies unless otherwise noted. Harmonic vibration frequency calculations at the same level were performed to verify all stationary points as local minima (with no imaginary frequency) or transition states (with one imaginary frequency). IRC calculations were also performed to check transition states. ACID calculation was performed by use of POV-Ray.

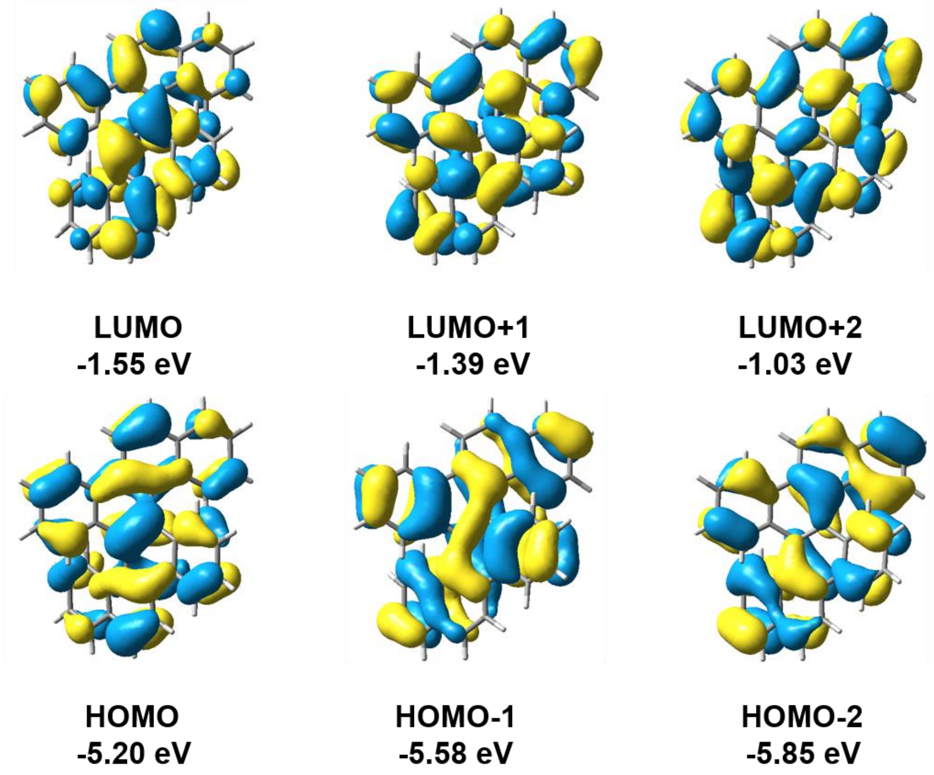

Fig. S5 Frontier molecular orbitals and orbital energies of (*P*,*P*)-**5d**.

Table S3 Cartesian coordinates of optimized species of **5d**

(*P,P*)-5d

C	-2.95580000	0.09240500	0.68893600
C	-1.69508500	1.93735100	-0.35028100
C	-0.35597400	3.74831700	-1.46557500
H	-1.25023900	4.35078900	-1.57934700
C	-3.99635000	2.30089700	0.36951200
H	-4.85440900	2.95688700	0.49442800
C	-0.73795100	-1.68150400	-0.75809600
C	0.73796000	1.68149400	-0.75810400
C	-0.44356700	2.47031700	-0.86878600
C	0.84172900	4.24128900	-1.95000600
H	0.87660600	5.22357100	-2.41315000
C	2.95580100	-0.09240200	0.68894000
C	1.69509600	-1.93736600	-0.35027800
C	-0.63745400	-0.29222400	-0.32096500
C	2.00107700	3.45393300	-1.86854000
H	2.93945500	3.81604600	-2.27947500
C	0.44357700	-2.47033000	-0.86878200
C	-4.13664100	-1.56718700	2.04372700
H	-4.13824300	-2.53078500	2.54606100
C	-1.78482200	0.56615300	-0.03235900
C	3.01201000	1.16309900	1.35429700
H	2.14746900	1.81385900	1.33666600
C	2.83160600	-2.77943900	-0.15686700
H	2.76704600	-3.82920600	-0.41836300
C	1.78483200	-0.56616200	-0.03235900
C	-5.23650300	0.51089100	1.53242200
H	-6.08526900	1.18589200	1.61474700
C	4.08159000	-0.96196800	0.84389000
C	4.13660700	1.56721000	2.04373000
H	4.13821600	2.53081500	2.54605200
C	0.63746600	0.29220300	-0.32096400
C	-3.01203400	-1.16309000	1.35429500
H	-2.14751200	-1.81387500	1.33667200
C	-2.00108300	-3.45394800	-1.86851000
H	-2.93946400	-3.81606200	-2.27943900
C	1.94493600	2.20047400	-1.28907900
H	2.83612700	1.58616500	-1.27254600
C	-1.94493900	-2.20049500	-1.28905300
H	-2.83613400	-1.58618700	-1.27249400
C	0.35597100	-3.74833800	-1.46557200
H	1.25023500	-4.35080600	-1.57935000
C	-2.83158400	2.77943900	-0.15688300
H	-2.76699800	3.82919800	-0.41840600
C	-4.08158000	0.96198500	0.84388600
C	3.99637000	-2.30087900	0.36951600
H	4.85443600	-2.95686400	0.49442900
C	5.23650400	-0.51085800	1.53243600
H	6.08527200	-1.18585200	1.61479000
C	5.27494700	0.73627400	2.11470800
H	6.16097400	1.06792300	2.64911600

C	-0.84173200	-4.24130600	-1.94999100
H	-0.87662300	-5.22358900	-2.41312900
C	-5.27497200	-0.73624300	2.11469500
H	-6.16100700	-1.06787500	2.64909600

TS-1

C	-3.26074000	-0.15605500	0.33780500
C	-1.85699900	1.81235200	-0.01380700
C	-0.62802900	3.98306300	0.02592300
H	-1.55940100	4.51104400	0.19735300
C	-4.15236000	1.83153400	-0.83444400
H	-4.96742600	2.32297200	-1.35942200
C	-0.46086900	-1.76795300	0.29551300
C	0.61031700	1.86871200	-0.03140800
C	-0.62382800	2.56298600	0.02857400
C	0.52900000	4.70563600	-0.16271500
H	0.50719500	5.79178100	-0.14367500
C	3.16759400	0.04123300	0.27452300
C	1.69465300	-1.51506100	-0.88676400
C	-0.60264200	-0.28688000	0.35103200
C	1.72775400	4.01667900	-0.42125000
H	2.63437200	4.56438000	-0.66348300
C	0.48000600	-2.29306400	-0.64436000
C	-4.93414400	-1.83394100	1.01878600
H	-5.17032900	-2.69926600	1.63238800
C	-1.90036300	0.42676200	0.29360800
C	3.41094100	0.89067000	1.38926800
H	2.56492200	1.29766300	1.93265700
C	2.82371900	-2.04759600	-1.57686000
H	2.69212600	-2.88525600	-2.25351500
C	1.83905700	-0.33147100	-0.14653100
C	-5.61483300	-0.08681300	-0.45450800
H	-6.37553100	0.43990400	-1.02593600
C	4.29846500	-0.55152600	-0.37296500
C	4.69403000	1.18664100	1.79637800
H	4.85017800	1.82439600	2.66223800
C	0.60230600	0.42240200	0.13101100
C	-3.66433400	-1.28651300	1.08820600
H	-2.99420300	-1.70753100	1.80758300
C	-1.07531100	-4.07937500	0.83069900
H	-1.58351600	-4.77474700	1.49274000
C	1.76211500	2.63976700	-0.36062500
H	2.69587900	2.14737100	-0.57832200
C	-1.09083700	-2.71864000	1.11721100
H	-1.52572800	-2.39789700	2.05144600
C	0.41523300	-3.65626600	-0.99568200
H	1.08151800	-4.04581200	-1.75779800
C	-2.99440200	2.48606700	-0.56899100
H	-2.87819800	3.50875900	-0.90382800
C	-4.33535900	0.50893400	-0.34786500
C	4.07764100	-1.53812800	-1.37571900
H	4.93082900	-1.94558500	-1.91243000
C	5.60747600	-0.19185100	0.03850500
H	6.45455000	-0.63278000	-0.48198200
C	5.80722000	0.66258300	1.10046900
H	6.81480300	0.91545900	1.41894100
C	-0.39672800	-4.54031000	-0.30018300

H	-0.41982800	-5.59170500	-0.57279900
C	-5.91530500	-1.26556500	0.19265500
H	-6.90447300	-1.70822000	0.11577600

***(P,M)*-5d**

C	-0.64235400	0.27169700	-0.09187200
C	1.80368900	0.61295900	0.13573700
C	4.25373400	1.03583500	0.28512900
C	2.68340700	2.77048900	0.89682600
H	2.49217500	3.77012700	1.27120200
C	1.83977500	-2.29945900	1.01854700
H	2.58955000	-1.66095600	1.46783900
C	-1.80368200	-0.61302800	-0.13528900
C	-4.25362600	-1.03590300	-0.28572400
C	-1.83986200	2.29965500	-1.01774400
H	-2.58973400	1.66123500	-1.46695700
C	0.64226800	-0.27154900	0.09275800
C	-1.58837200	-1.97504700	-0.43699400
C	3.49876700	-0.92567700	-0.94236100
H	2.70113900	-1.52106700	-1.36848100
C	-3.96116200	-2.29173000	-0.89341100
H	-4.77991800	-2.89783900	-1.27327300
C	-0.29615900	-2.55955100	-0.11881800
C	1.96087600	-3.67091700	1.14169400
H	2.80234700	-4.09247000	1.68447100
C	0.29629500	2.55962000	0.11916600
C	0.75565100	-1.71026000	0.32577400
C	-3.17261000	-0.20419900	0.14541900
C	0.99214700	-4.51291100	0.57103700
H	1.09051000	-5.59247200	0.64624700
C	-0.75569600	1.71036700	-0.32508800
C	-1.96088200	3.67109600	-1.14097200
H	-2.80242000	4.09263000	-1.68366400
C	1.58852500	1.97510900	0.43711300
C	-0.99197500	4.51308300	-0.57059900
H	-1.09024300	5.59264200	-0.64592700
C	-5.59186900	-0.64116000	-0.03560800
H	-6.39583100	-1.27630100	-0.40039700
C	-0.12343200	-3.96207000	-0.03063600
H	-0.90927800	-4.62391500	-0.37672800
C	3.17256400	0.20404200	-0.14540400
C	5.87193300	-0.49348300	-0.69566400
H	6.90107300	-0.77684200	-0.89877000
C	-3.49915700	0.92540900	0.94243100
H	-2.70174900	1.52076900	1.36902600
C	-2.68302700	-2.77042000	-0.89716800
H	-2.49166400	-3.77005600	-1.27148400
C	-5.87224800	0.49329300	0.69455800
H	-6.90146600	0.77668500	0.89721100
C	5.59185600	0.64108300	0.03445400
H	6.39598800	1.27626600	0.39878700
C	4.80928000	-1.26208800	-1.21547500
H	5.02103100	-2.12278400	-1.84410700
C	3.96151100	2.29176900	0.89273500
H	4.78039800	2.89786000	1.27234700
C	-4.80978800	1.26177800	1.21498600
H	-5.02178500	2.12242700	1.84360300

C	0.12366000	3.96218000	0.03085900
H	0.90967300	4.62397600	0.37667500

(TS-2)

C	-8.84501200	-4.90670300	-6.91514900
C	-7.39159400	-6.41479000	-8.29454800
C	-5.44269200	-7.28604400	-9.63312500
C	-5.19494000	-6.96545800	-7.26839800
H	-4.57112400	-6.94689500	-6.38408800
C	-9.90728800	-5.66691800	-10.37887300
H	-10.06658100	-6.73022800	-10.28449100
C	-9.80034700	-3.78403400	-6.86948100
C	-11.29251200	-2.18869400	-5.69618600
C	-8.19974400	-4.54500000	-4.47473500
H	-8.87845800	-3.71316600	-4.37810000
C	-8.57262700	-5.53009500	-8.15633300
C	-9.81430900	-2.93206400	-7.98451200
C	-7.60871800	-7.49147400	-10.63408000
H	-8.67338200	-7.55345000	-10.55451800
C	-11.00254400	-1.23326400	-6.71130300
H	-11.39323900	-0.22347000	-6.61297400
C	-9.58161500	-3.55696000	-9.28646400
C	-10.38737300	-5.02738500	-11.51662700
H	-10.81649800	-5.61451500	-12.32362900
C	-6.99984700	-6.14333000	-5.83740400
C	-9.33247500	-4.96481000	-9.30527700
C	-10.70894400	-3.49273200	-5.78767800
C	-10.32358800	-3.63464400	-11.60608900
H	-10.64075000	-3.12006400	-12.50880200
C	-8.05860300	-5.20756100	-5.72805200
C	-7.48341900	-4.92737800	-3.36059000
H	-7.63289400	-4.40127100	-2.42171900
C	-6.56695000	-6.56689500	-7.14862600
C	-6.57260600	-5.99645500	-3.43889200
H	-6.05255400	-6.34730300	-2.55175300
C	-12.18930000	-1.89716600	-4.63641400
H	-12.60301100	-0.89366500	-4.56779400
C	-9.97265300	-2.91046900	-10.47603600
H	-10.09709000	-1.83300900	-10.48438900
C	-6.85303300	-7.02528800	-9.53142800
C	-5.64462100	-8.04735000	-11.93905700
H	-5.19406700	-8.40209300	-12.86179600
C	-11.14570900	-4.48180600	-4.86354900
H	-10.76246800	-5.49217500	-4.95801100
C	-10.34063900	-1.61341900	-7.84702600
H	-10.23707000	-0.90672700	-8.66357300
C	-12.56365100	-2.87056600	-3.73642500
H	-13.26601200	-2.64015700	-2.94006100
C	-4.86647300	-7.73220900	-10.84651900
H	-3.78789100	-7.86744700	-10.87806200
C	-7.03870400	-7.97518700	-11.79939400
H	-7.68699800	-8.32224600	-12.59960600
C	-4.62318100	-7.20334000	-8.47504700
H	-3.55660300	-7.39332100	-8.56317100
C	-12.05331500	-4.18196900	-3.87061100
H	-12.38206400	-4.96155300	-3.18872600
C	-6.31809000	-6.56738100	-4.66595100

H	-5.59603700	-7.37330200	-4.73436000

***(M,M)*-5d**

C	-0.63745700	0.29218700	-0.32090200
C	1.78483800	0.56618000	-0.03233500
C	4.08160100	0.96202500	0.84389600
C	2.83157600	2.77946700	-0.15685600
H	2.76700900	3.82922400	-0.41838300
C	1.94496100	-2.20039200	-1.28914000
H	2.83612500	-1.58602900	-1.27261500
C	-1.78481200	-0.56618500	-0.03228500
C	-4.08159200	-0.96201400	0.84390000
C	-1.94496100	2.20036800	-1.28907300
H	-2.83612900	1.58601600	-1.27249200
C	0.63747000	-0.29221500	-0.32092800
C	-1.69504500	-1.93739500	-0.35019100
C	3.01209500	-1.16307400	1.35427800
H	2.14759300	-1.81389000	1.33664900
C	-3.99632700	-2.30093200	0.36957300
H	-4.85437900	-2.95694100	0.49449000
C	-0.44352100	-2.47034000	-0.86870900
C	2.00112700	-3.45382400	-1.86862500
H	2.93948300	-3.81588100	-2.27966300
C	0.44353300	2.47032100	-0.86875000
C	0.73798100	-1.68148200	-0.75807500
C	-2.95581400	-0.09241700	0.68894200
C	0.84180600	-4.24124000	-1.95002000
H	0.87671900	-5.22351600	-2.41317400
C	-0.73796200	1.68146700	-0.75806700
C	-2.00115400	3.45380600	-1.86856500
H	-2.93953200	3.81585900	-2.27955900
C	1.69507900	1.93737500	-0.35025400
C	-0.84184500	4.24121700	-1.95001000
H	-0.87676800	5.22350100	-2.41314800
C	-5.23654000	-0.51091200	1.53238100
H	-6.08529600	-1.18592800	1.61473700
C	-0.35588400	-3.74834400	-1.46551800
H	-1.25011900	-4.35086800	-1.57923600
C	2.95582200	0.09242400	0.68893700
C	5.27502900	-0.73620000	2.11467100
H	6.16108400	-1.06781900	2.64904900
C	-3.01211000	1.16311000	1.35423800
H	-2.14760700	1.81392400	1.33659900
C	-2.83154400	-2.77948100	-0.15678000
H	-2.76697500	-3.82924900	-0.41826800
C	-5.27505300	0.73624300	2.11460400
H	-6.16111100	1.06787700	2.64896700
C	5.23653000	0.51094100	1.53241600
H	6.08528000	1.18596300	1.61478200
C	4.13671900	-1.56716100	2.04369500
H	4.13833700	-2.53077100	2.54600800
C	3.99635100	2.30092900	0.36952300
H	4.85440500	2.95693400	0.49442800
C	-4.13674200	1.56721100	2.04361800
H	-4.13838800	2.53083700	2.54590100
C	0.35587500	3.74831700	-1.46555100
H	1.25009500	4.35085800	-1.57929800

VIII. References

1 Y. Cui, L. Wang, F. Y. Kwong, M. K. Tse and A. S. C. Chan, *Synlett,* 2009, **16***,* 2696.

2 C. Wang, Y.-B. Yu, S. Fan and X. Zhang, *Org. Lett*., 2013, **15**, 5004.

IX. Copies of NMR spectra

^{1}H NMR spectra of **1o** ($CDCl_3$)

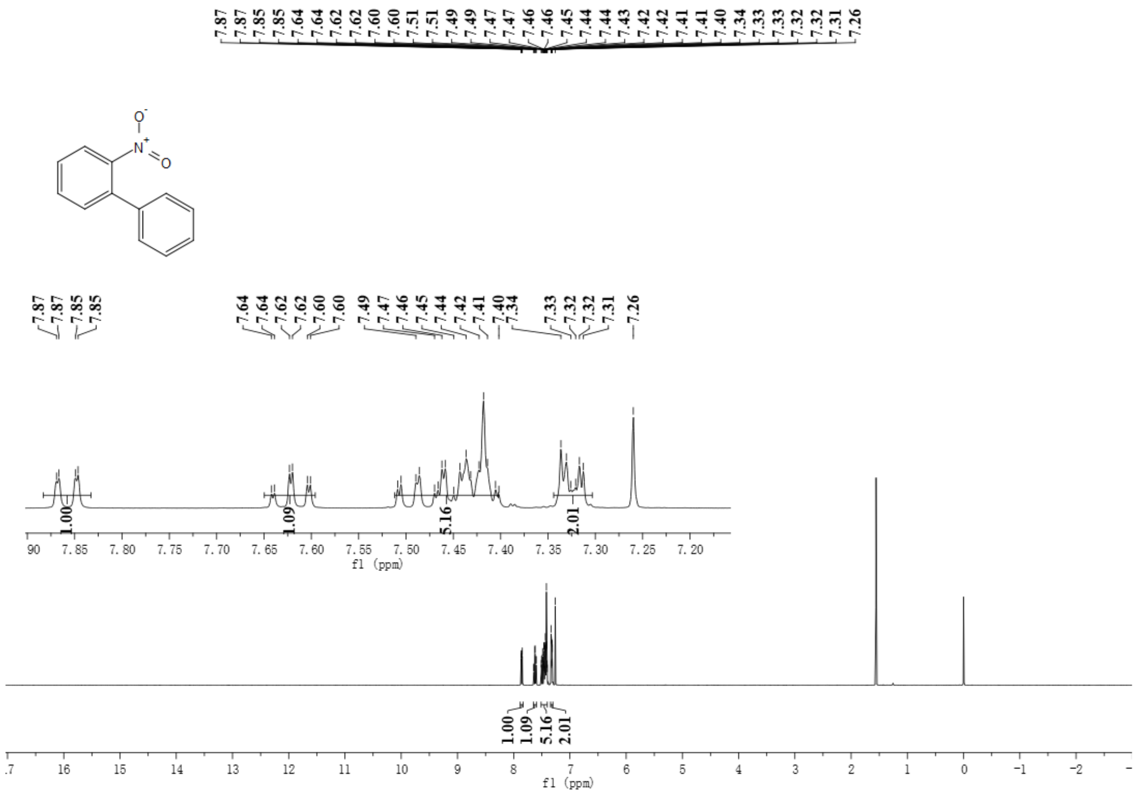

^{13}C NMR spectra of **1o** ($CDCl_3$)

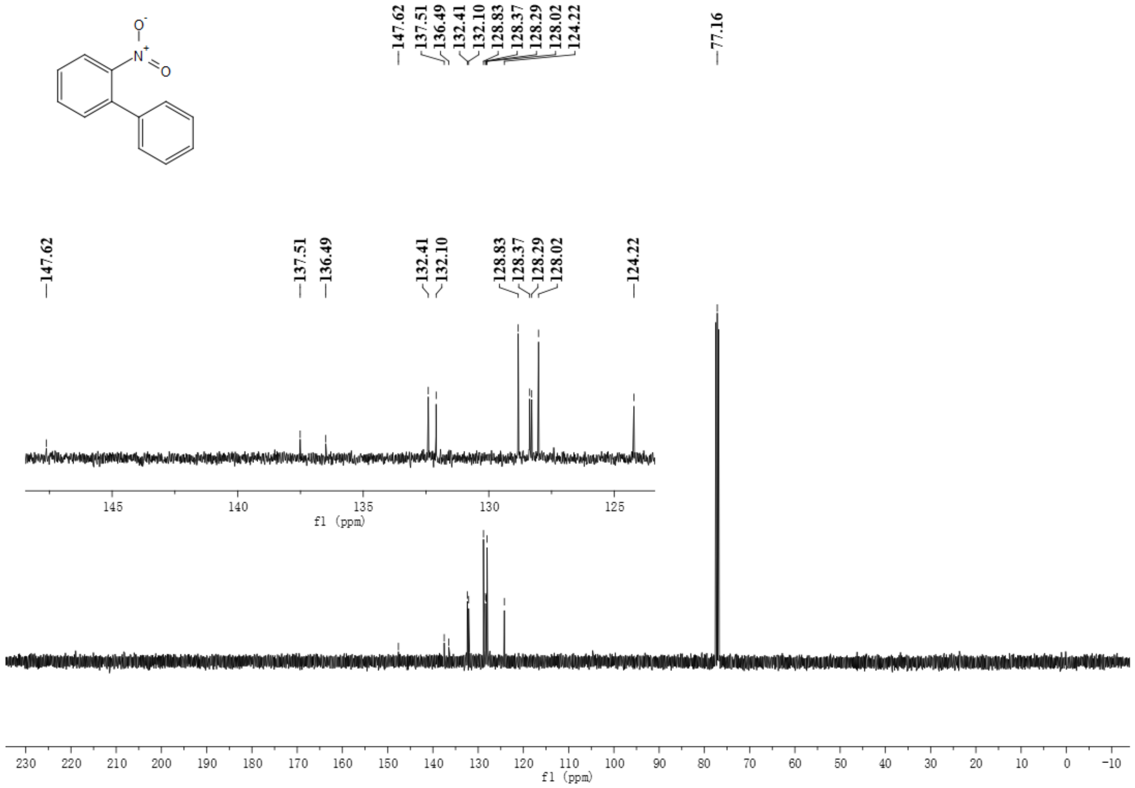

^{1}H NMR spectra of **1p** ($CDCl_3$)

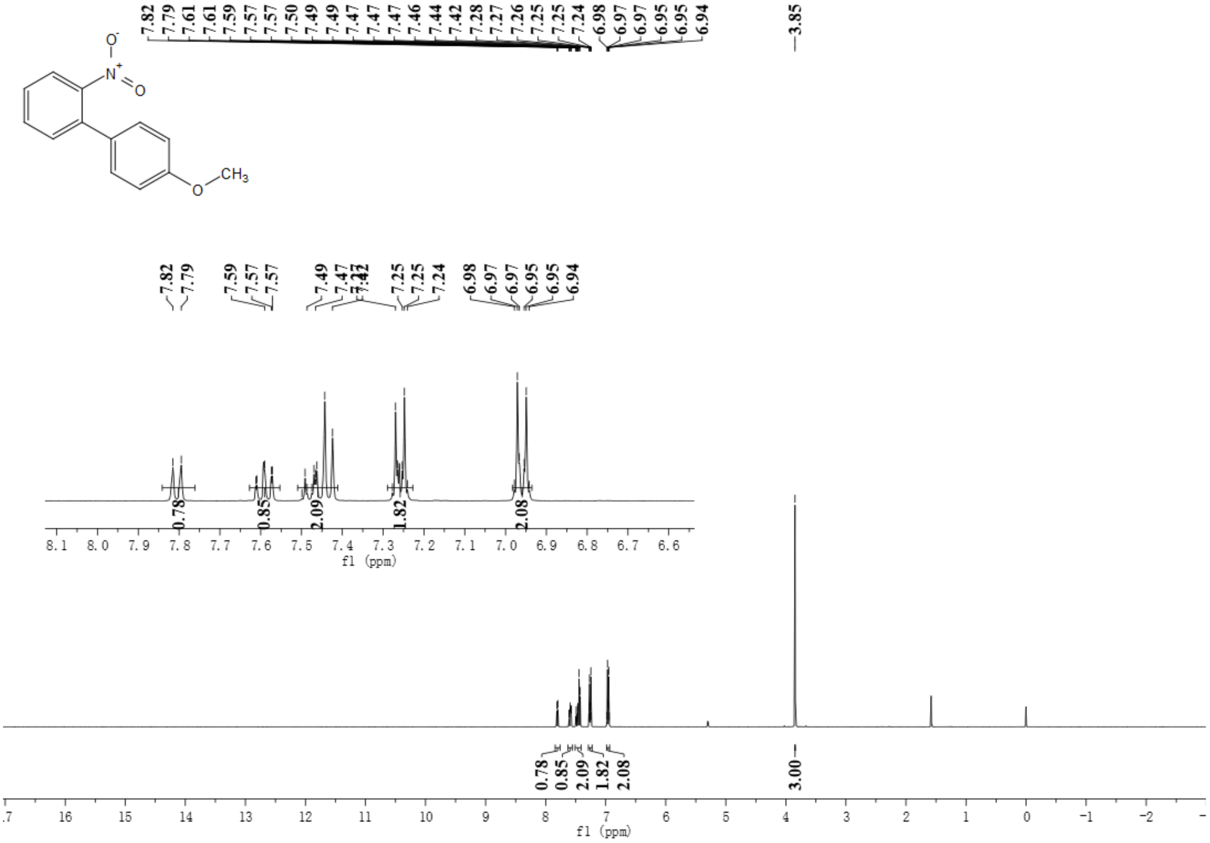

^{13}C NMR spectra of **1p** ($CDCl_3$)

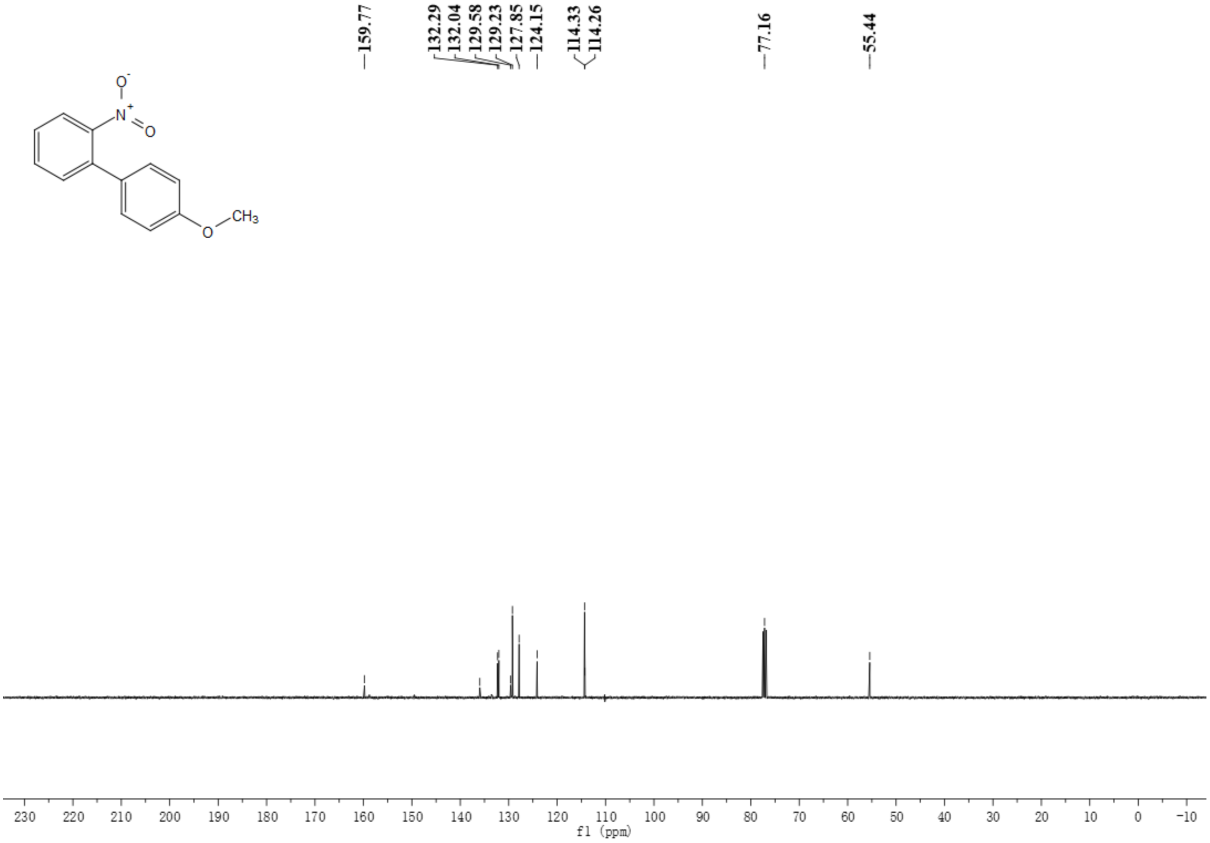

^{1}H NMR spectra of **1q** ($CDCl_3$)

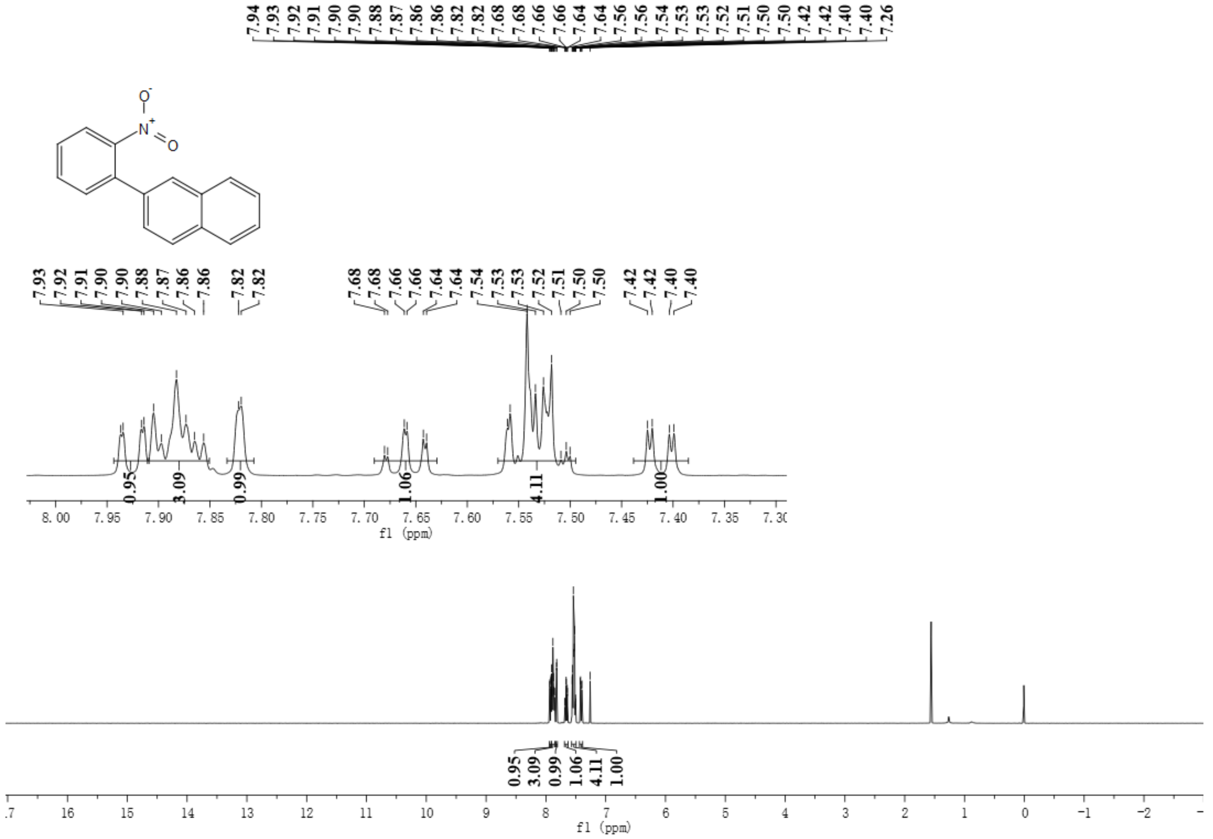

^{13}C NMR spectra of **1q** ($CDCl_3$)

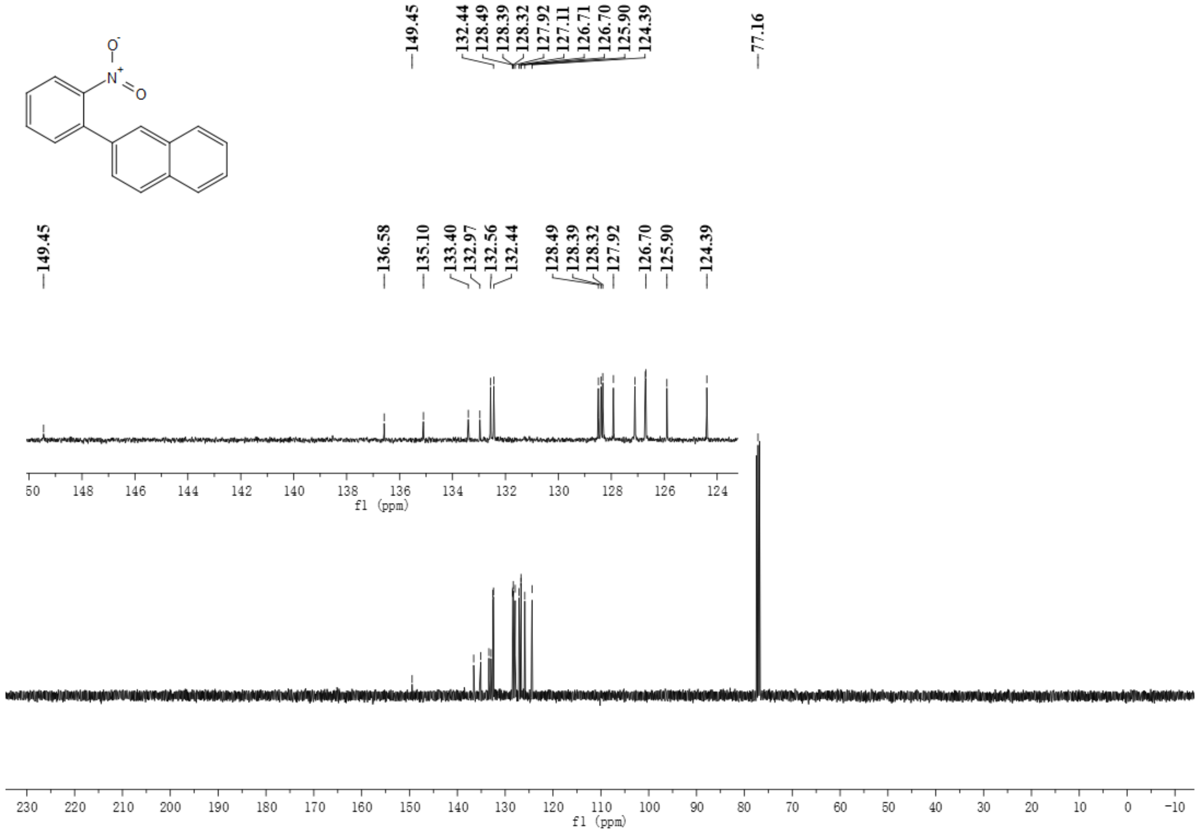

^{1}H NMR spectra of **1r** ($CDCl_3$)

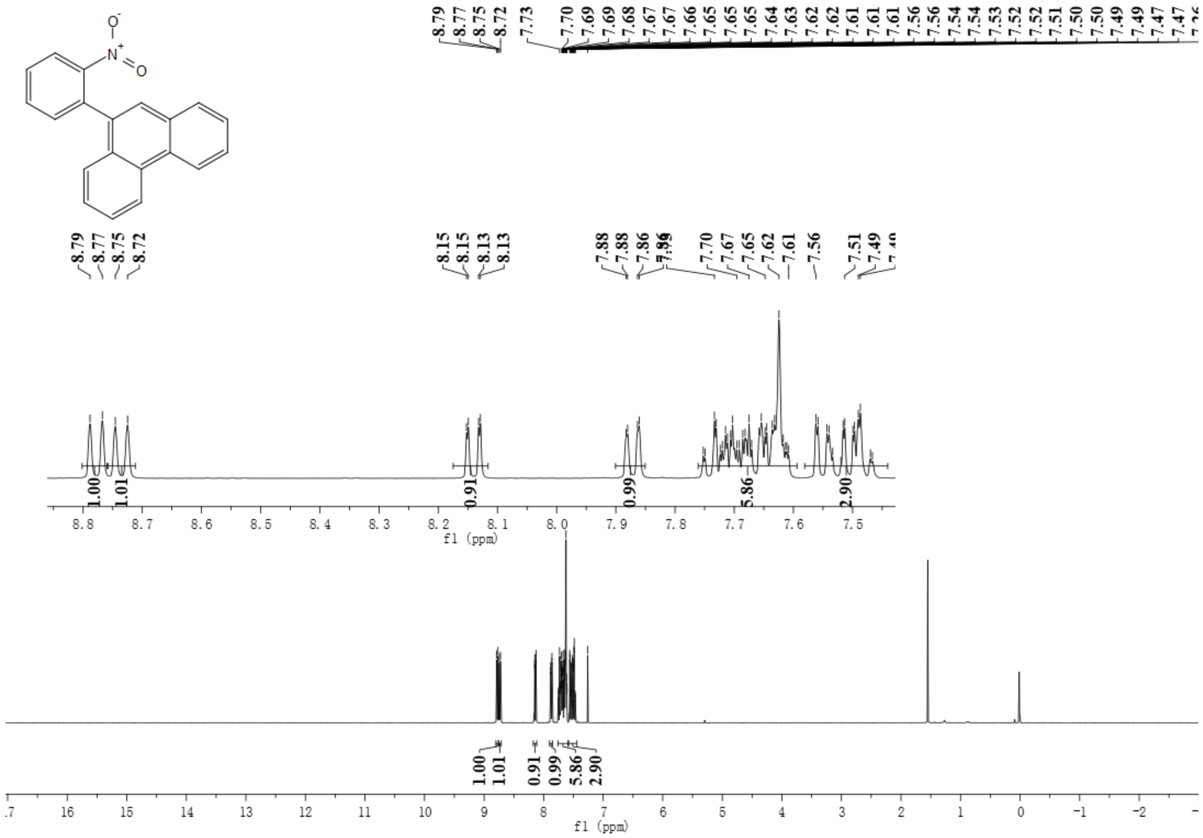

^{13}C NMR spectra of **1r** ($CDCl_3$)

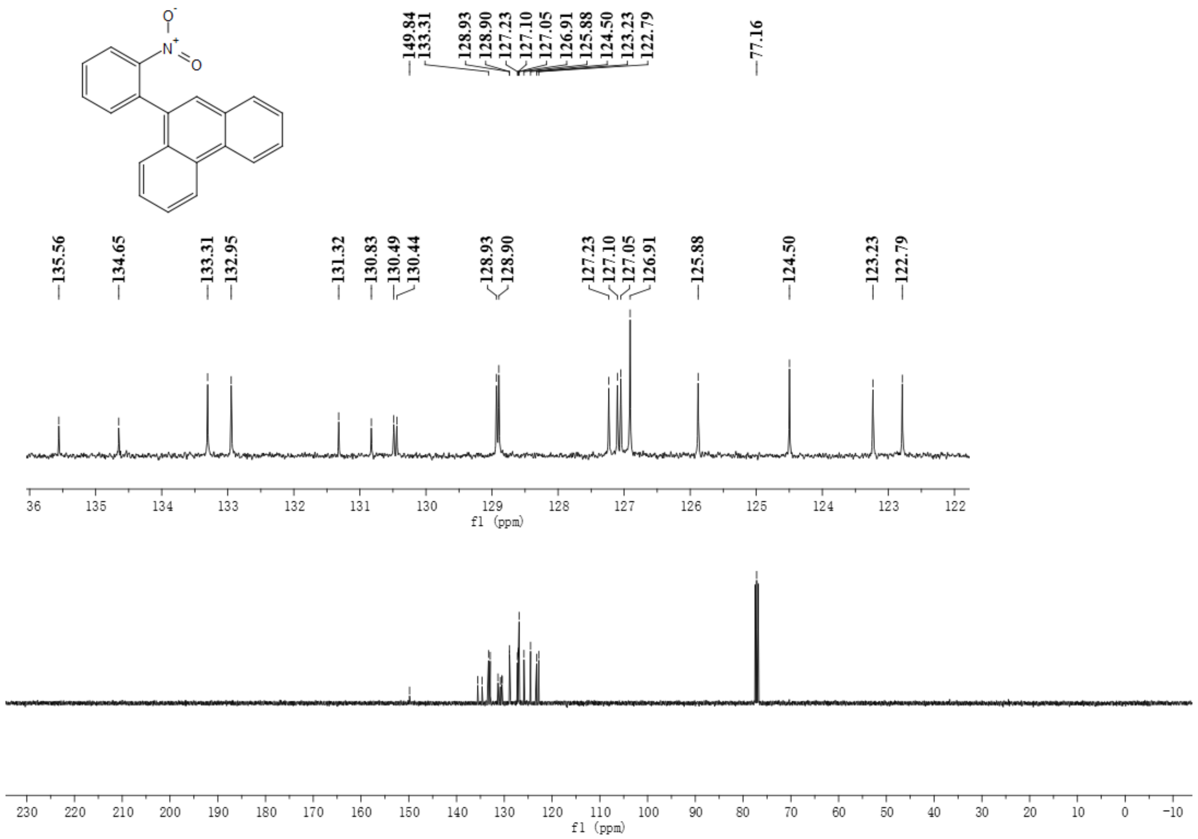

^{1}H NMR spectra of **1s** ($CDCl_3$)

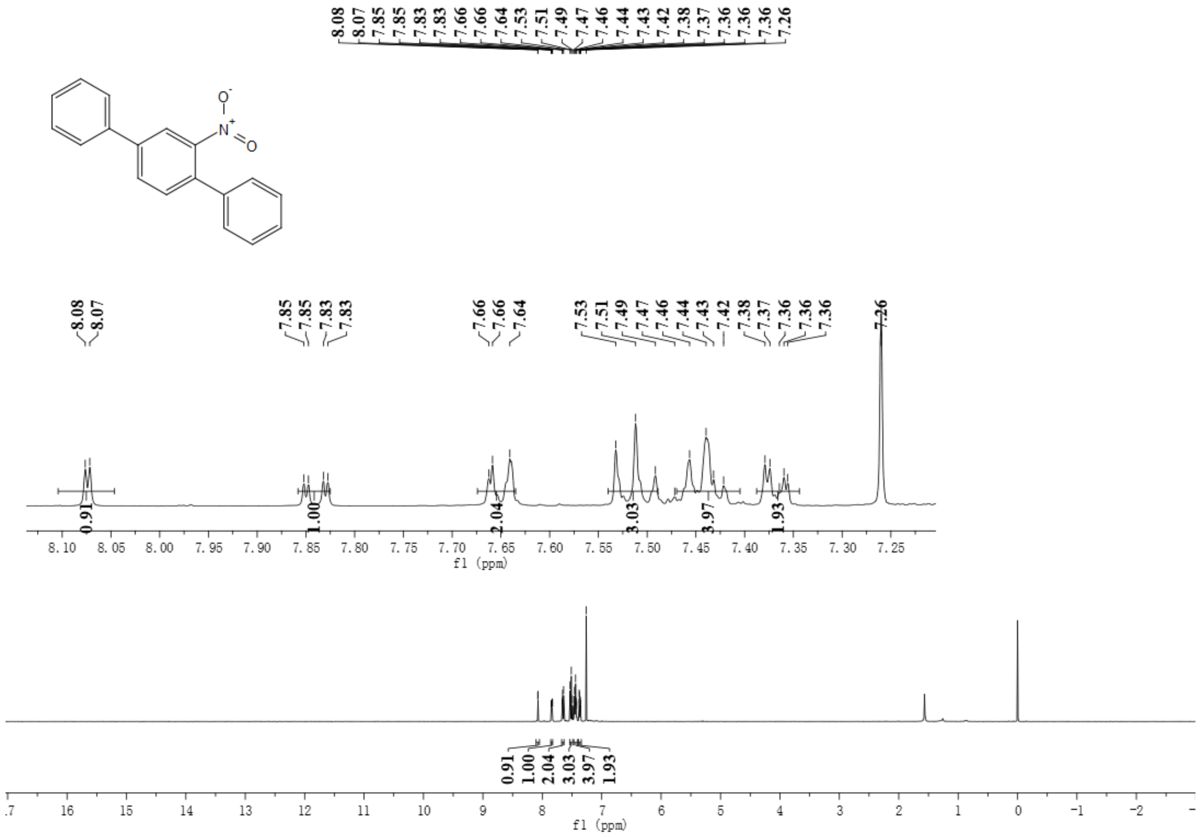

^{13}C NMR spectra of **1s** ($CDCl_3$)

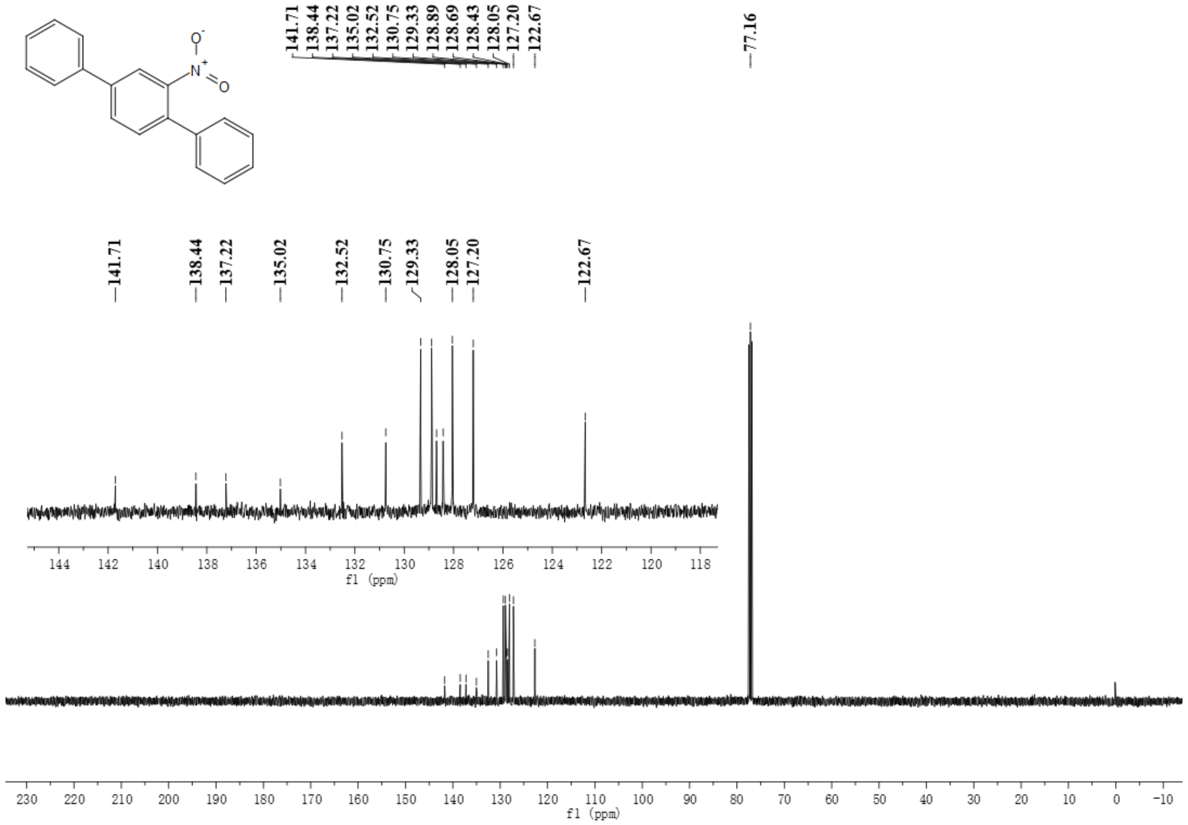

^{1}H NMR spectra of **1t** ($CDCl_3$)

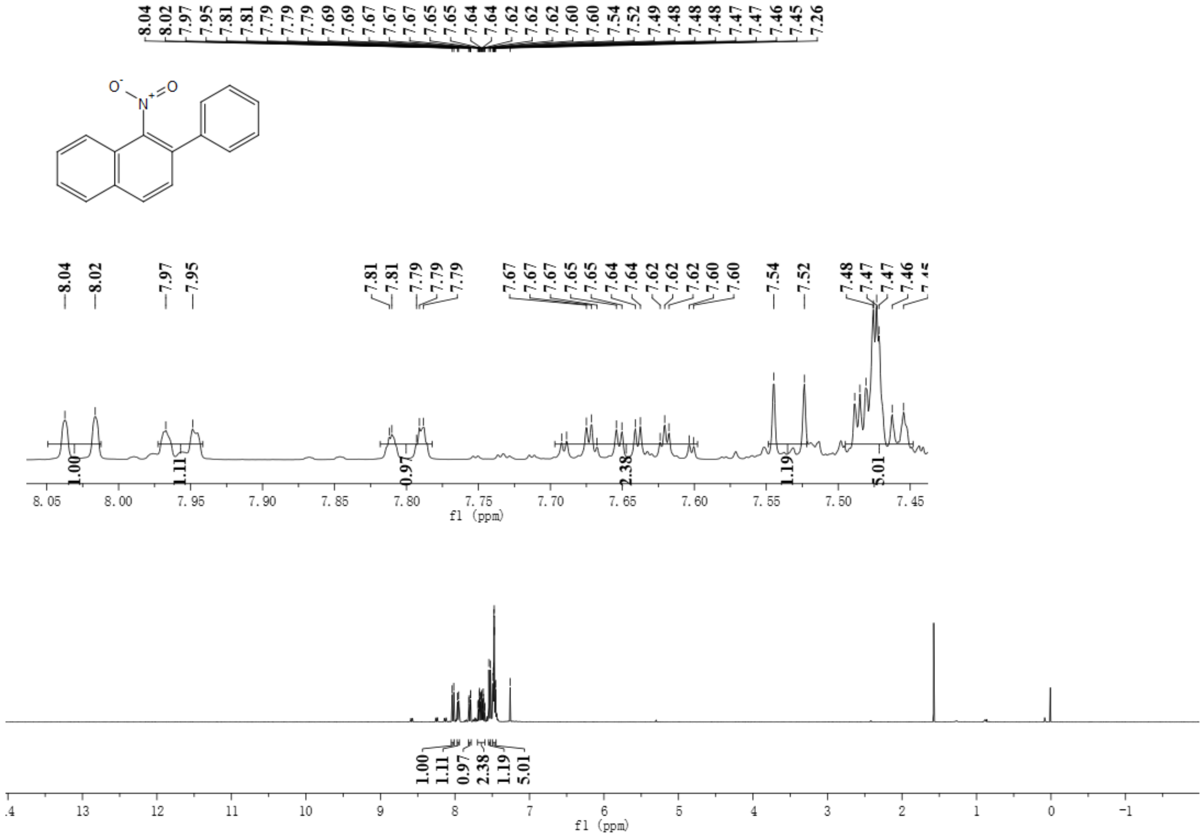

^{13}C NMR spectra of **1t** ($CDCl_3$)

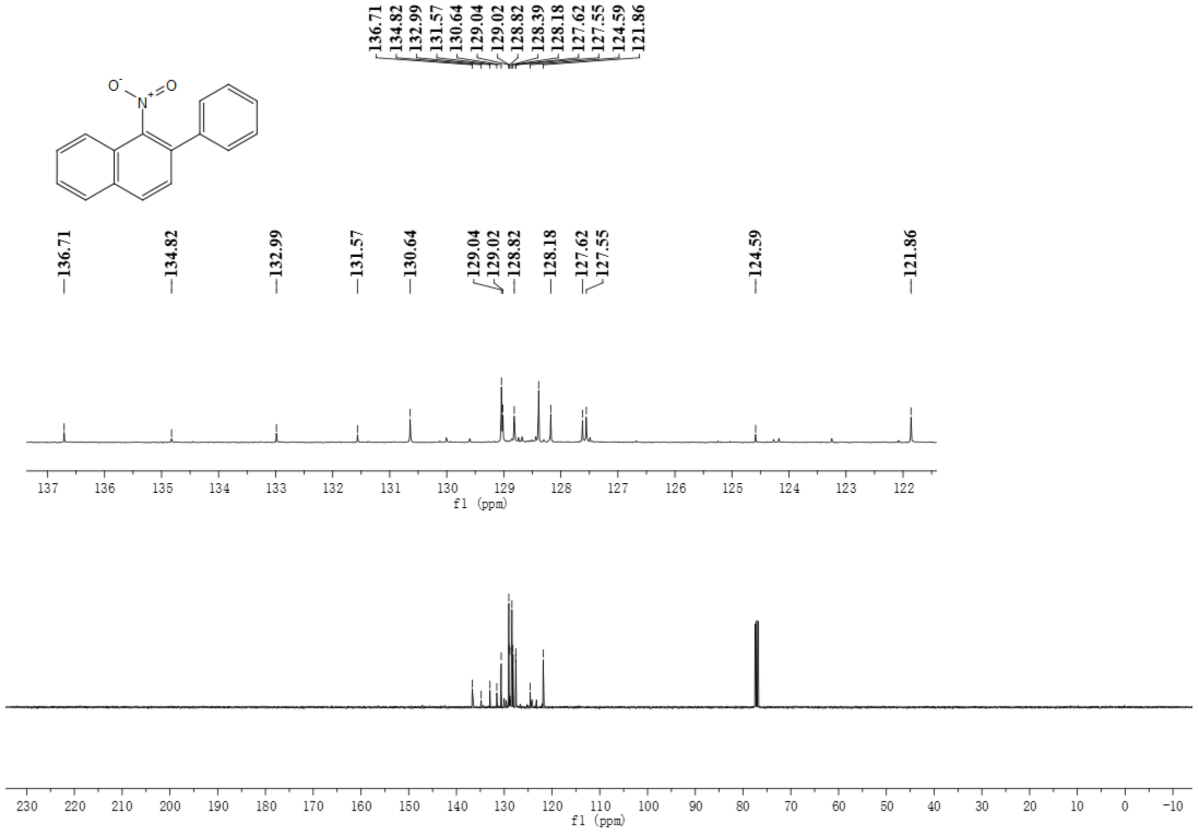

^{1}H NMR spectra of **2b** ($CDCl_3$)

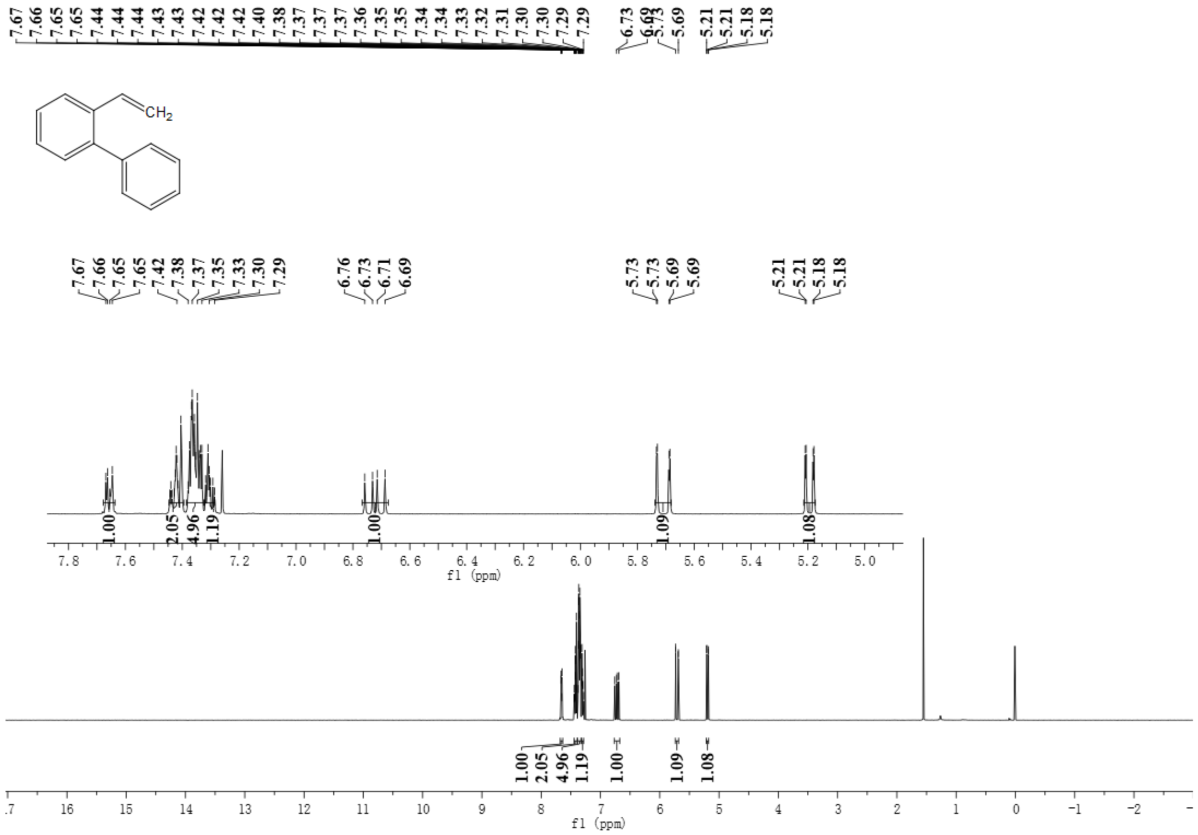

^{13}C NMR spectra of **2b** ($CDCl_3$)

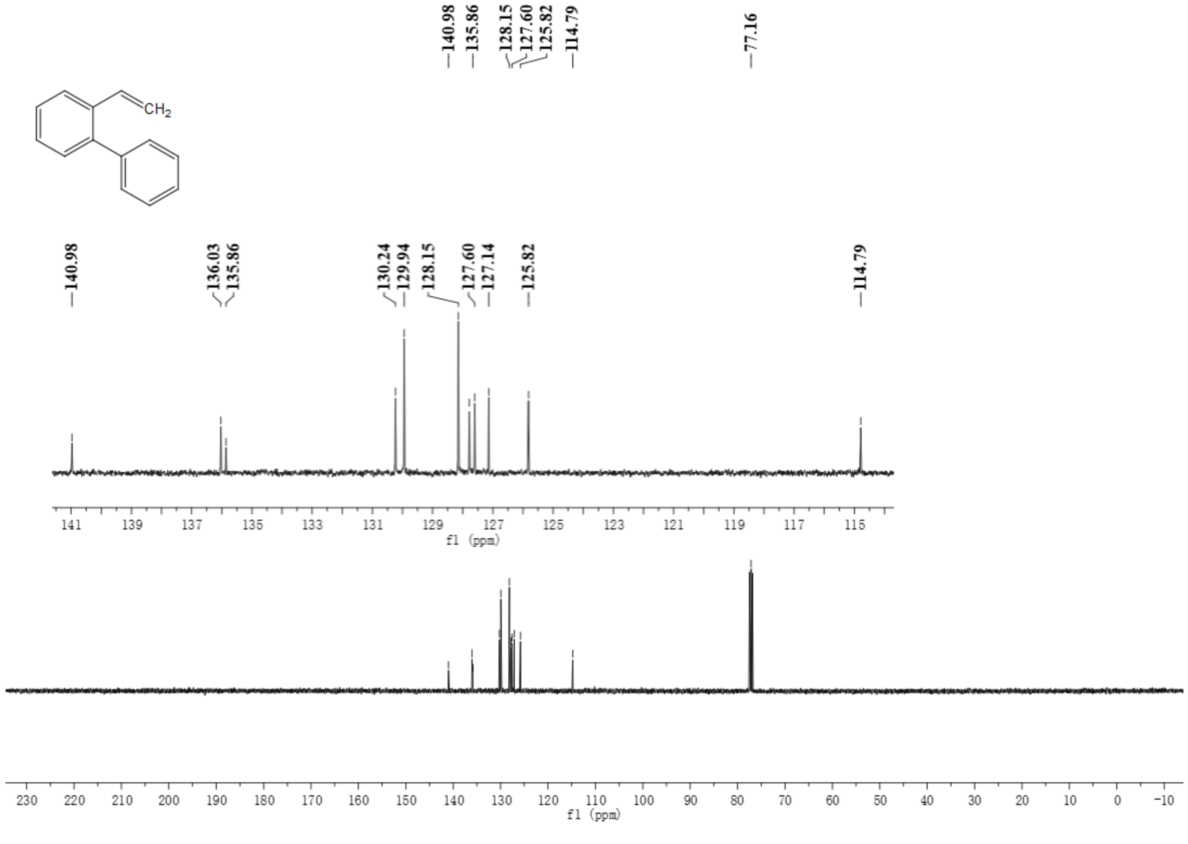

^{1}H NMR spectra of **2m** ($CDCl_3$)

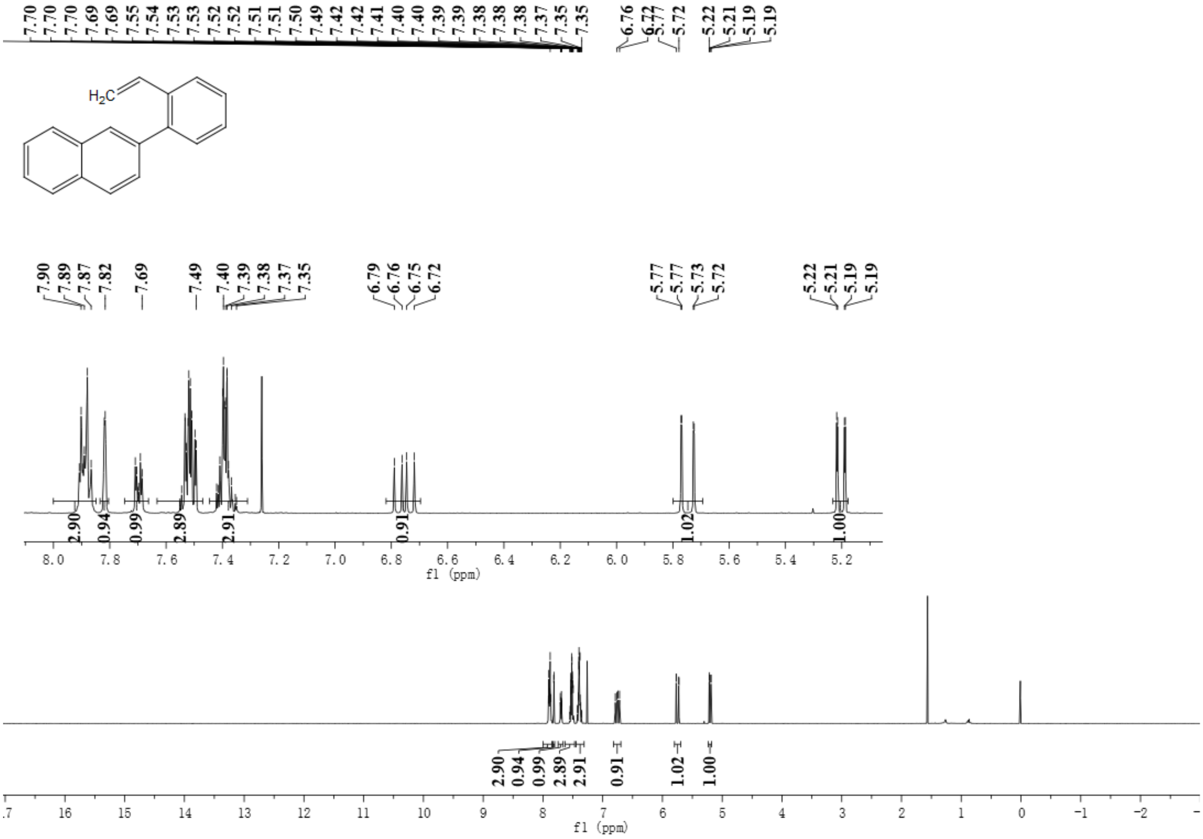

^{13}C NMR spectra of **2m** ($CDCl_3$)

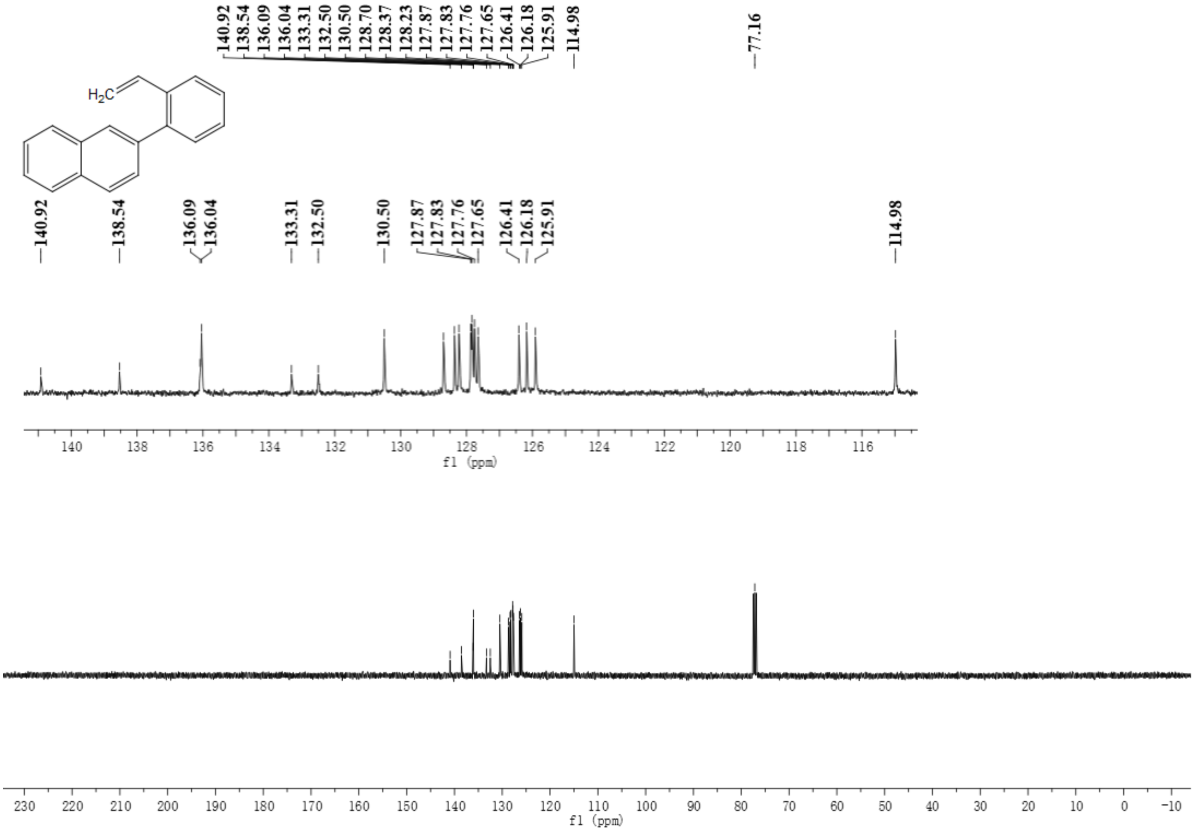

^{1}H NMR spectra of **3a** (DMSO-d_6)

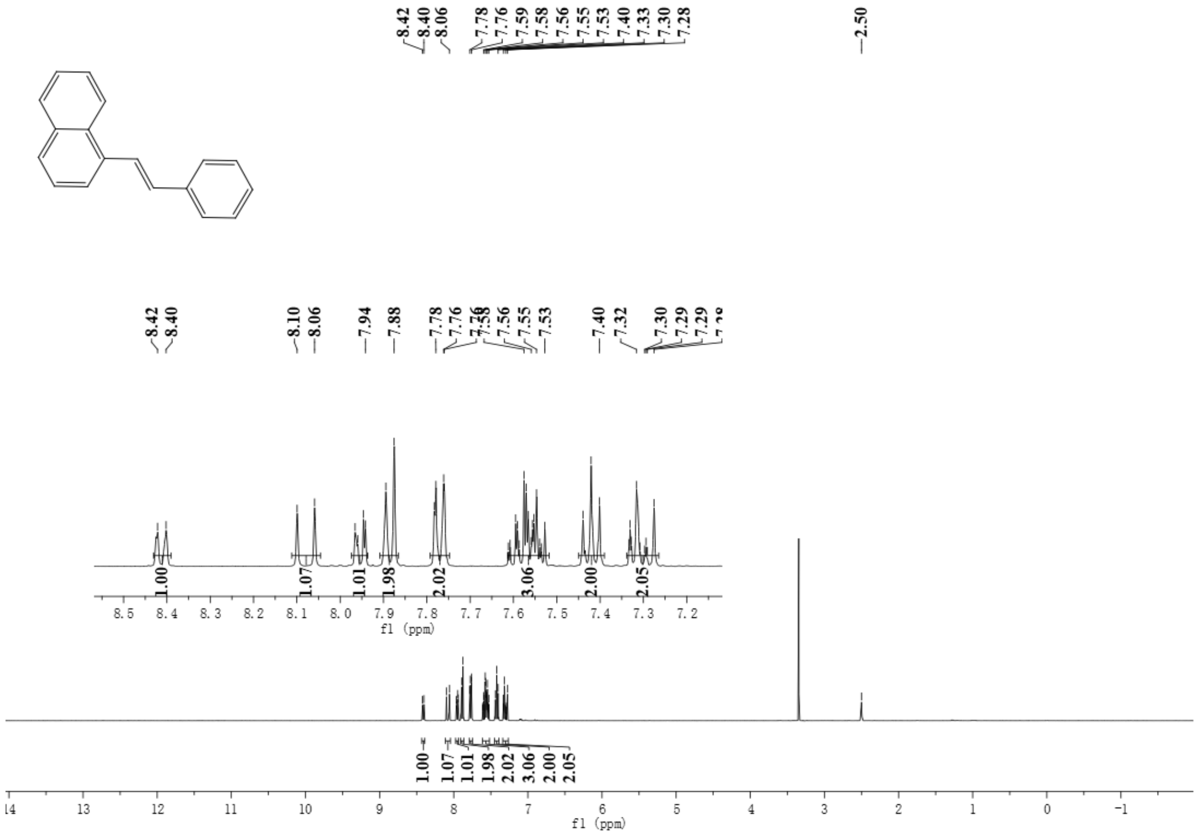

^{13}C NMR spectra of **3a** (DMSO-d_6)

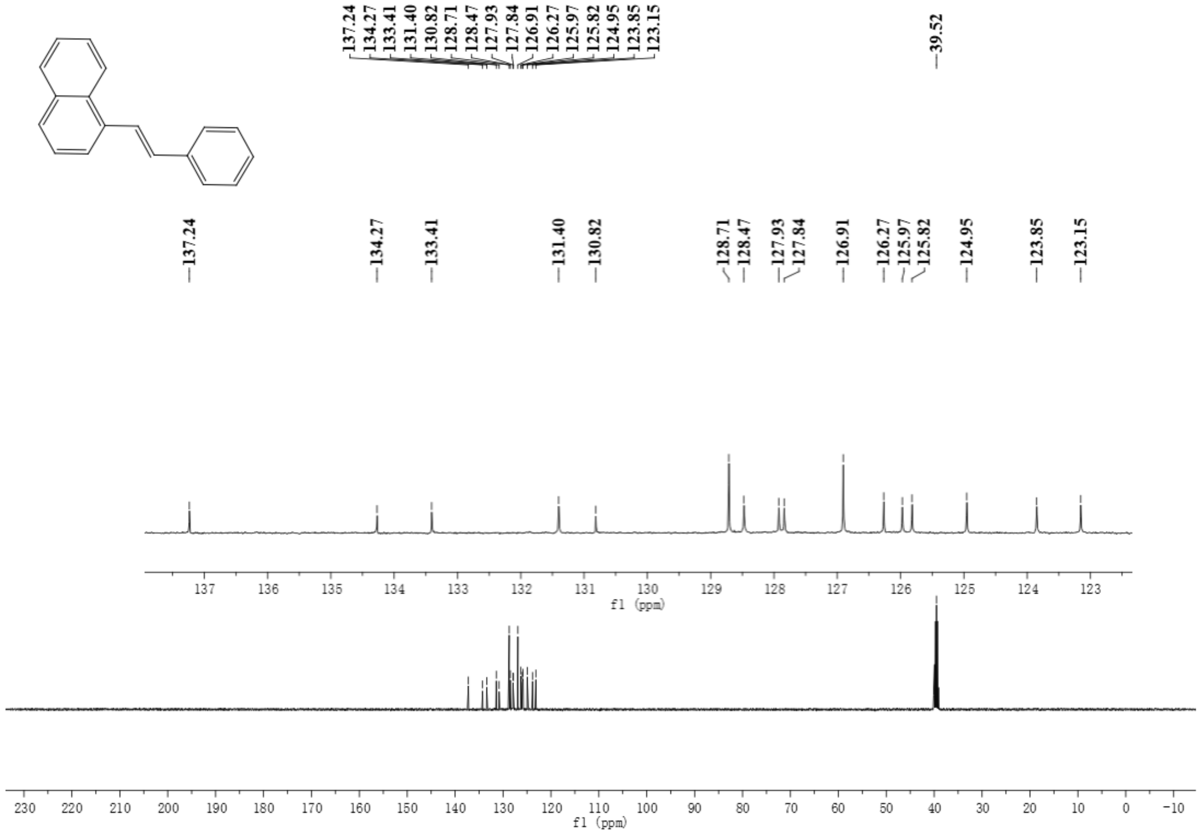

^{1}H NMR spectra of **3b** ($CDCl_3$)

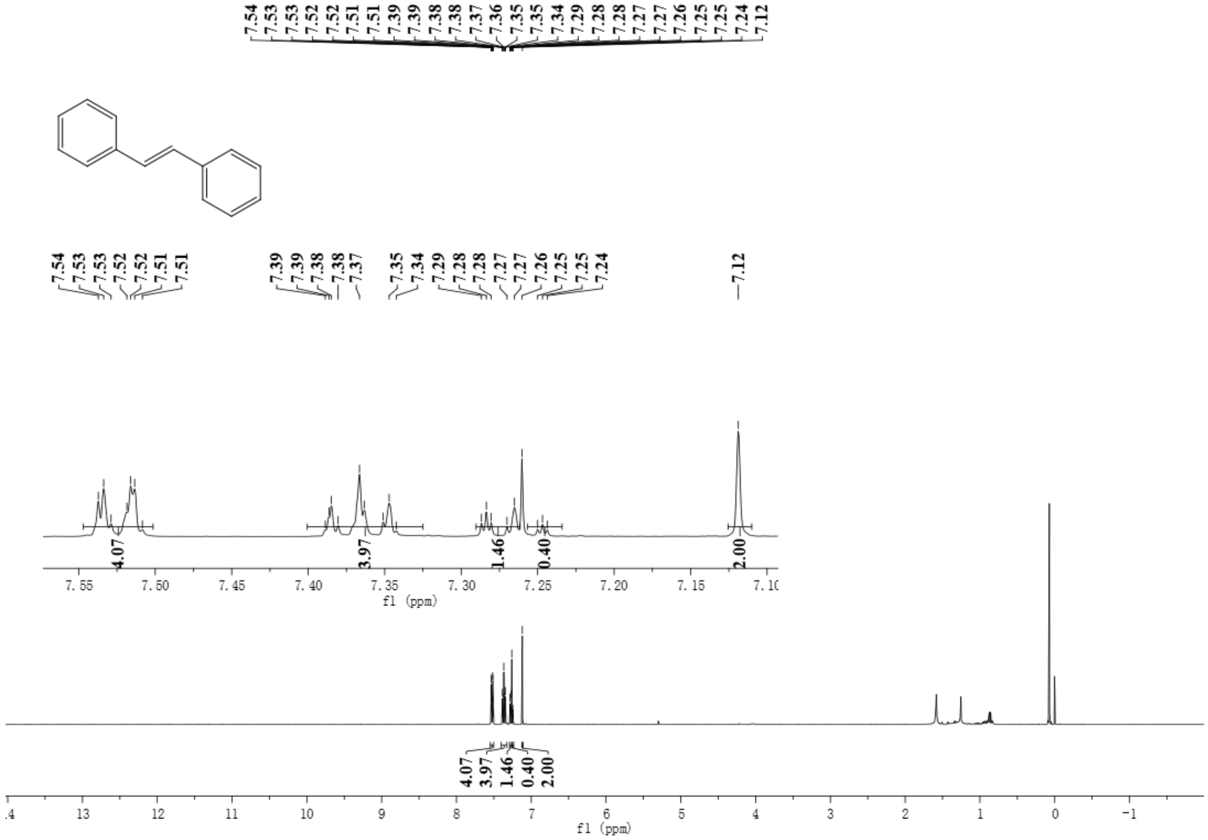

^{13}C NMR spectra of **3b** ($CDCl_3$)

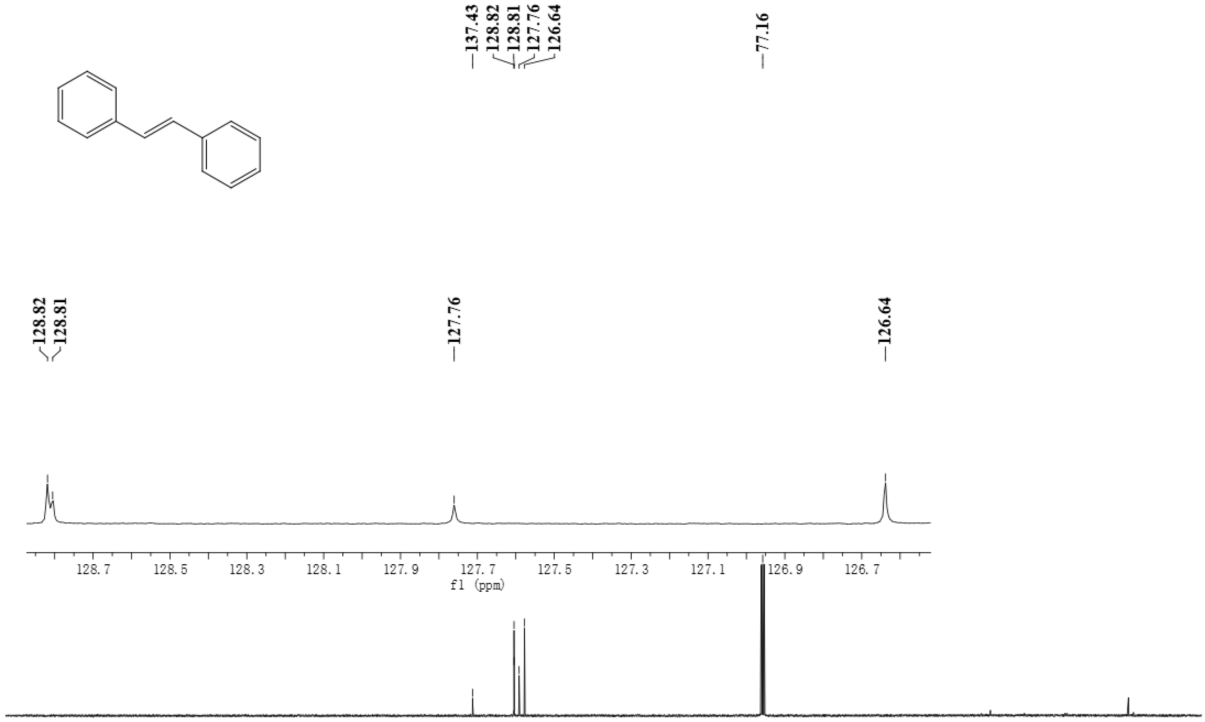

^{1}H NMR spectra of **3c** (DMSO-d_6)

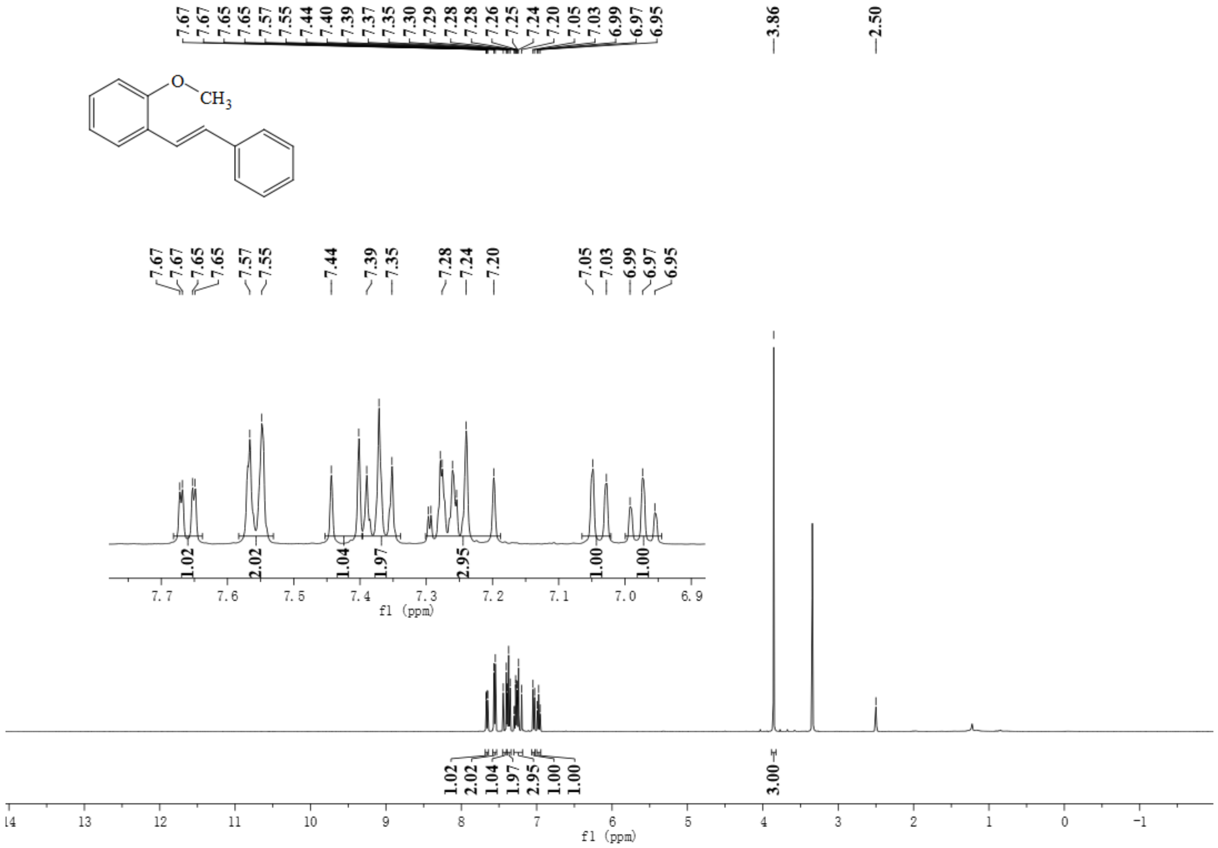

^{13}C NMR spectra of **3c** (DMSO-d_6)

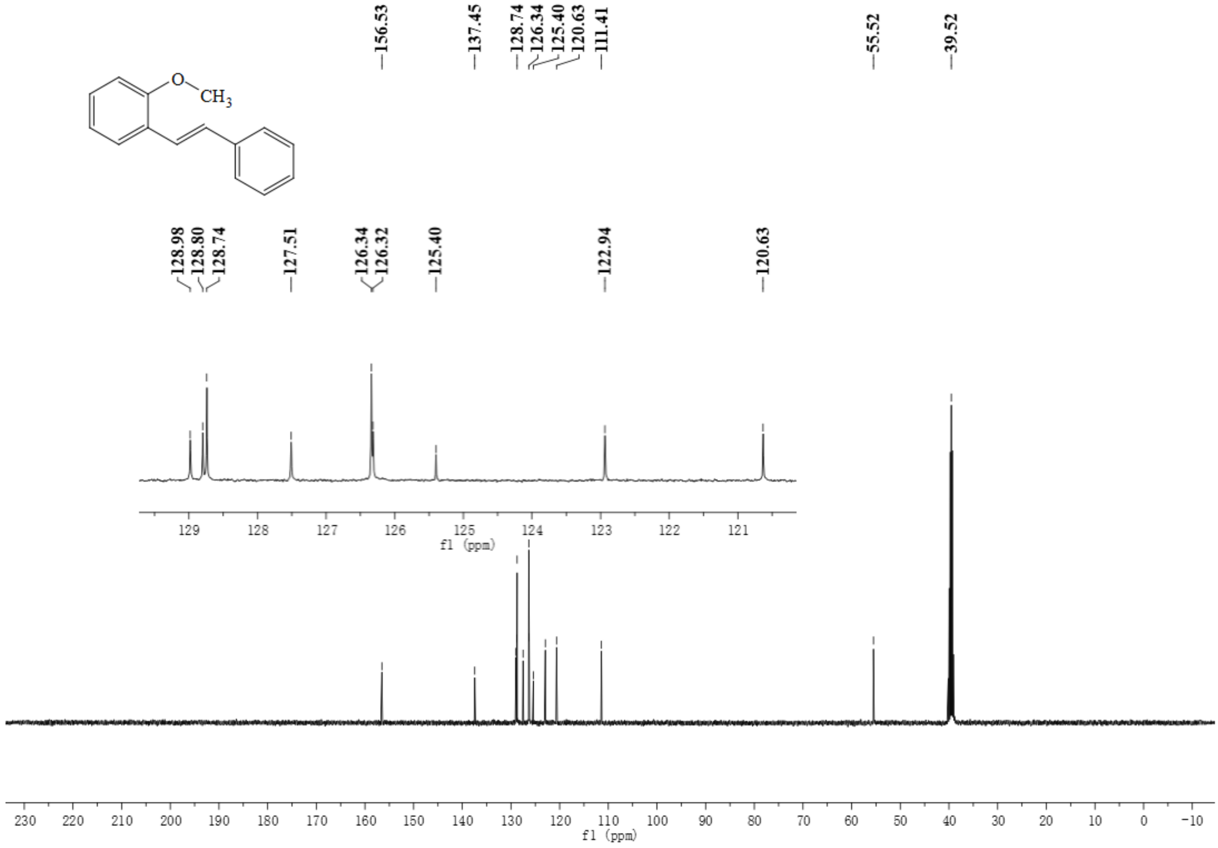

^{1}H NMR spectra of **3d** (DMSO-d_6)

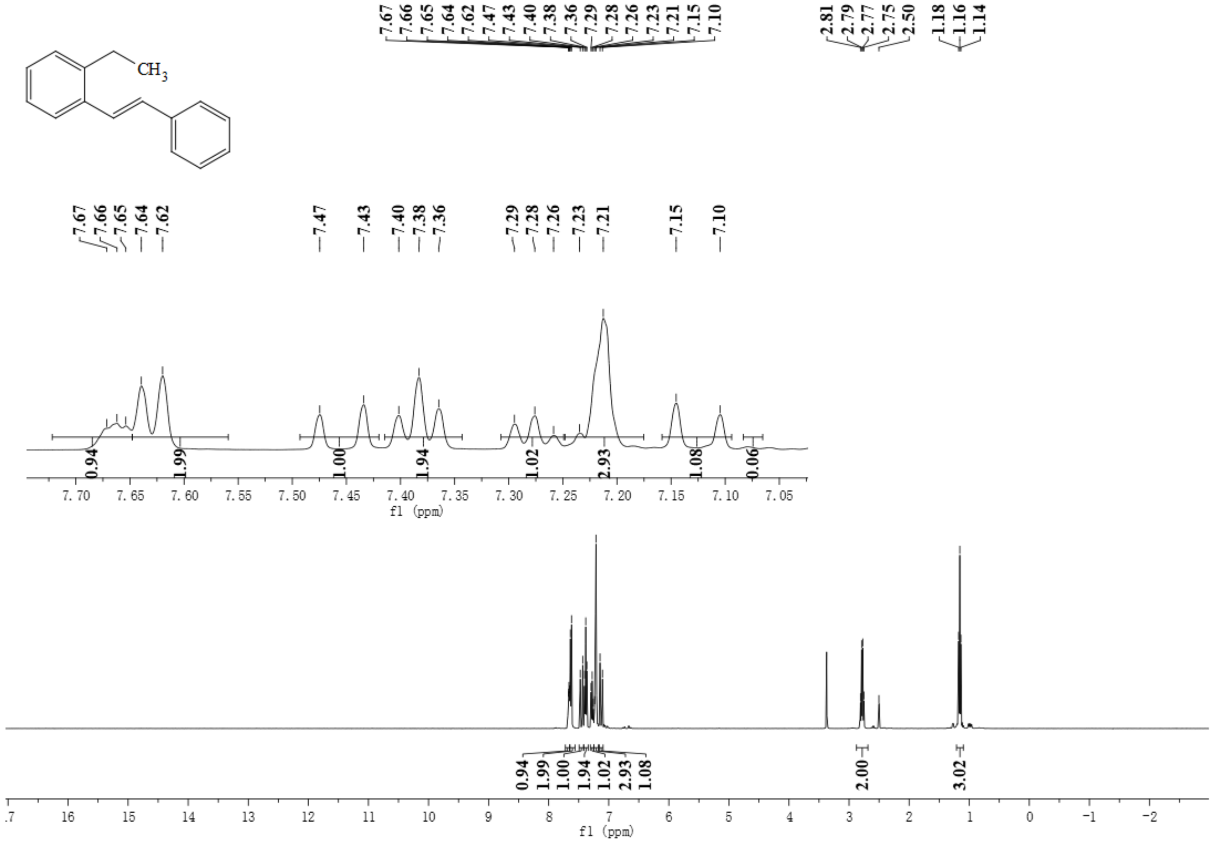

^{13}C NMR spectra of **3d** (DMSO-d_6)

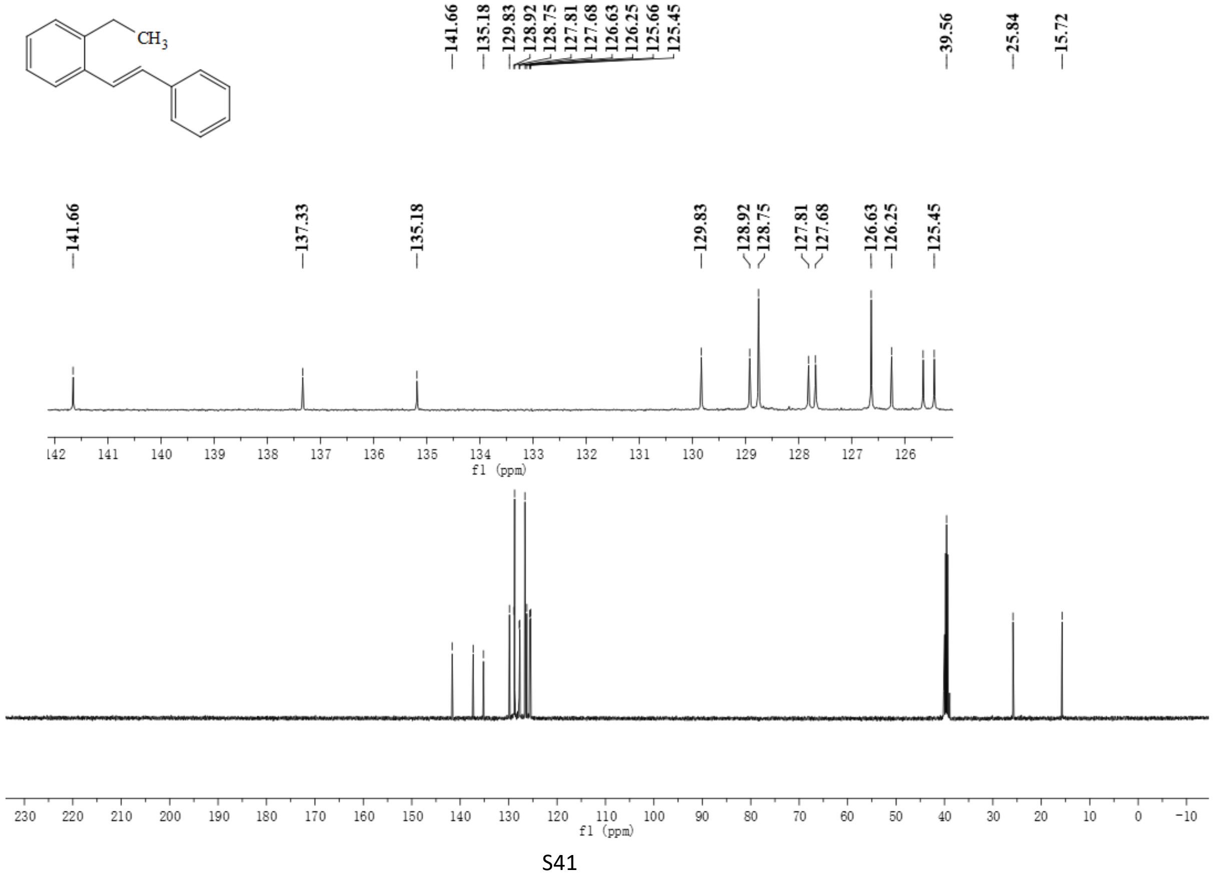

^{1}H NMR spectra of **3e** (DMSO-d_6)

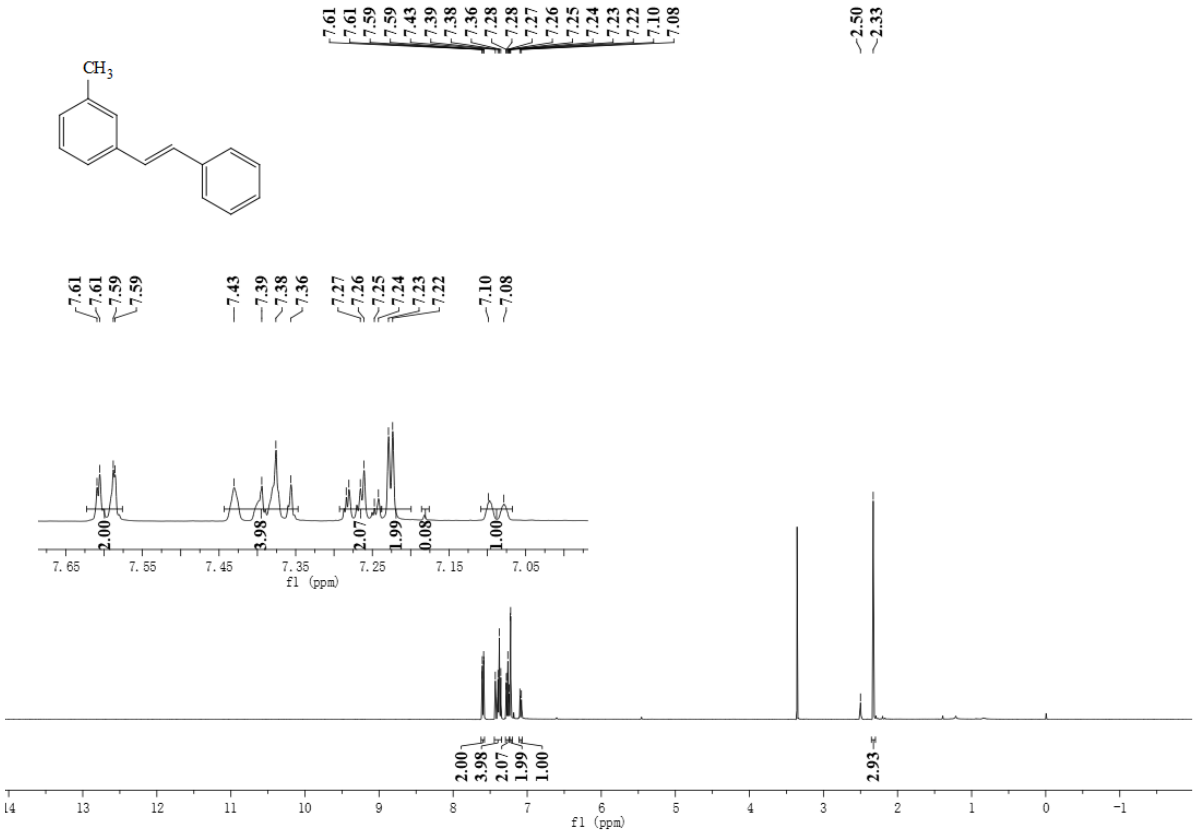

^{13}C NMR spectra of **3e** (DMSO-d_6)

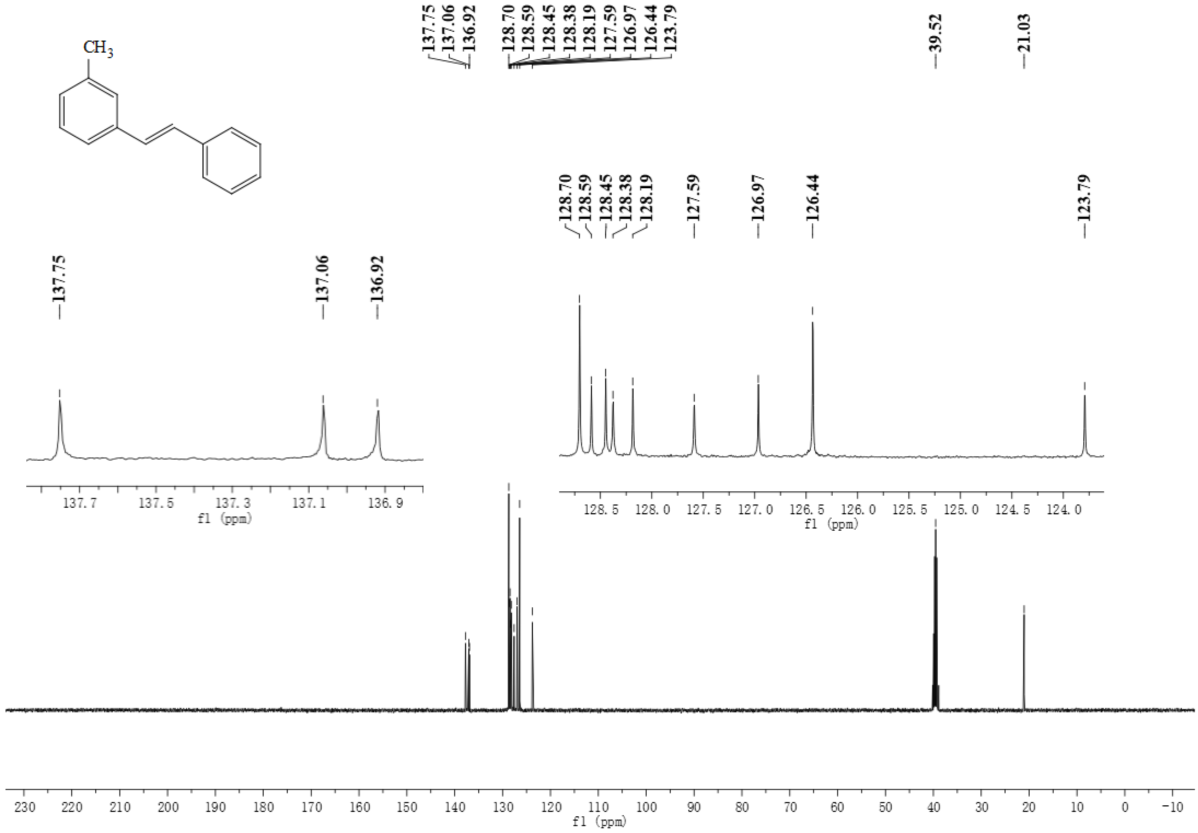

^{1}H NMR spectra of **3f** (DMSO-d_6)

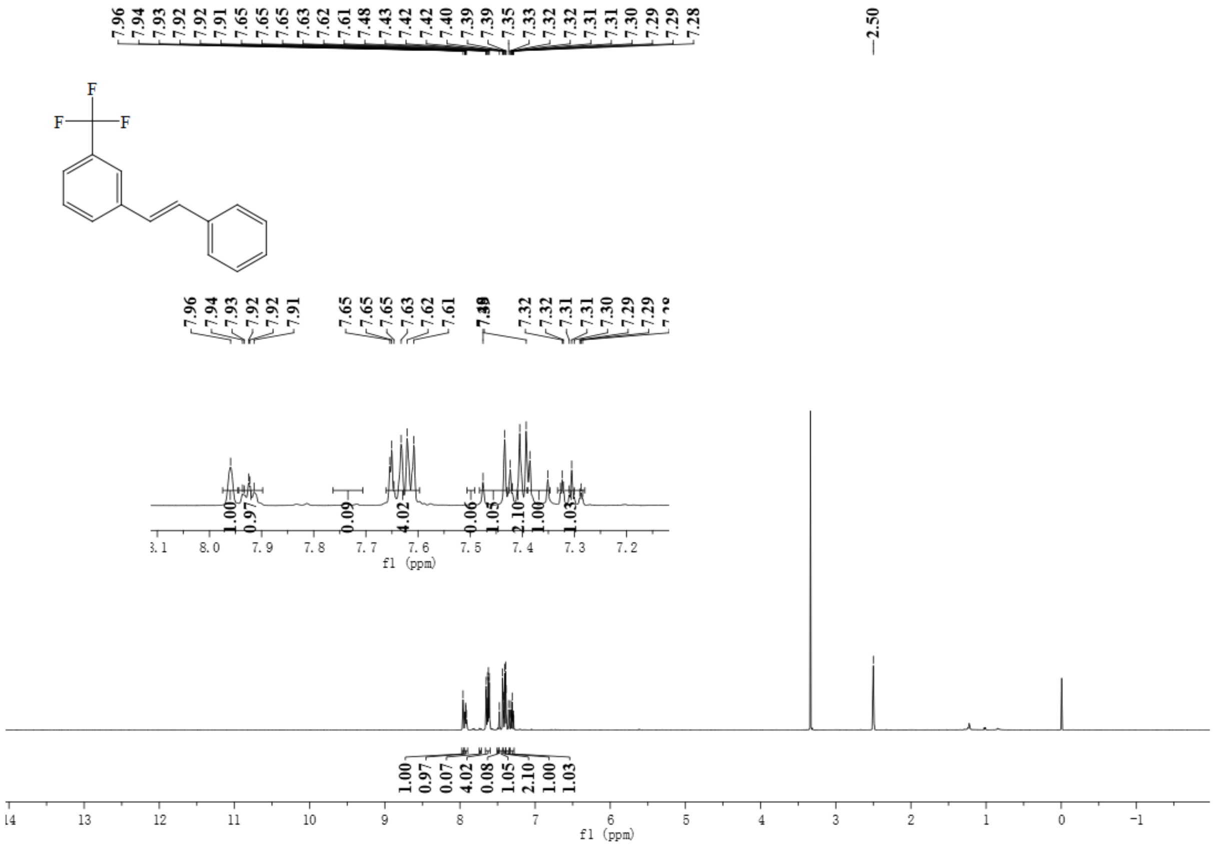

^{13}C NMR spectra of **3f** (DMSO-d_6)

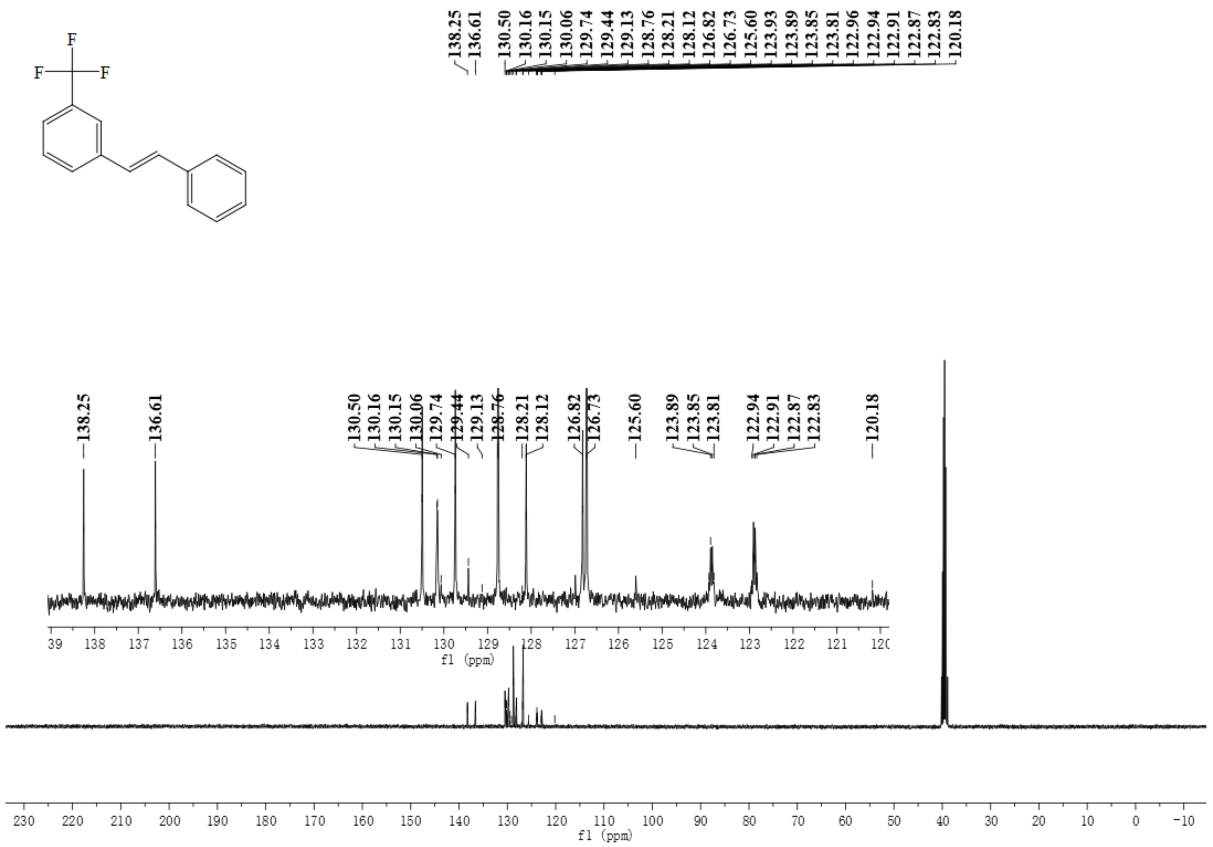

^{1}H NMR spectra of **3g** (DMSO-d_6)

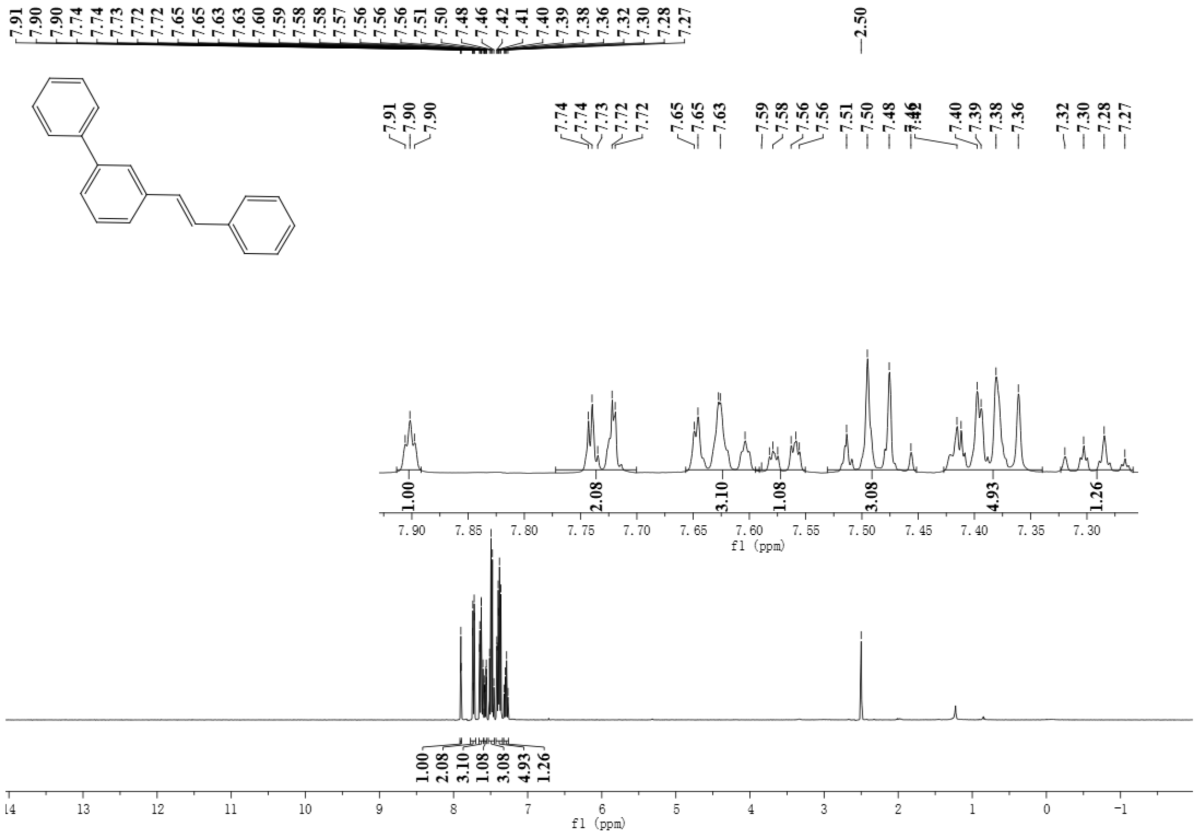

^{13}C NMR spectra of **3g** (DMSO-d_6)

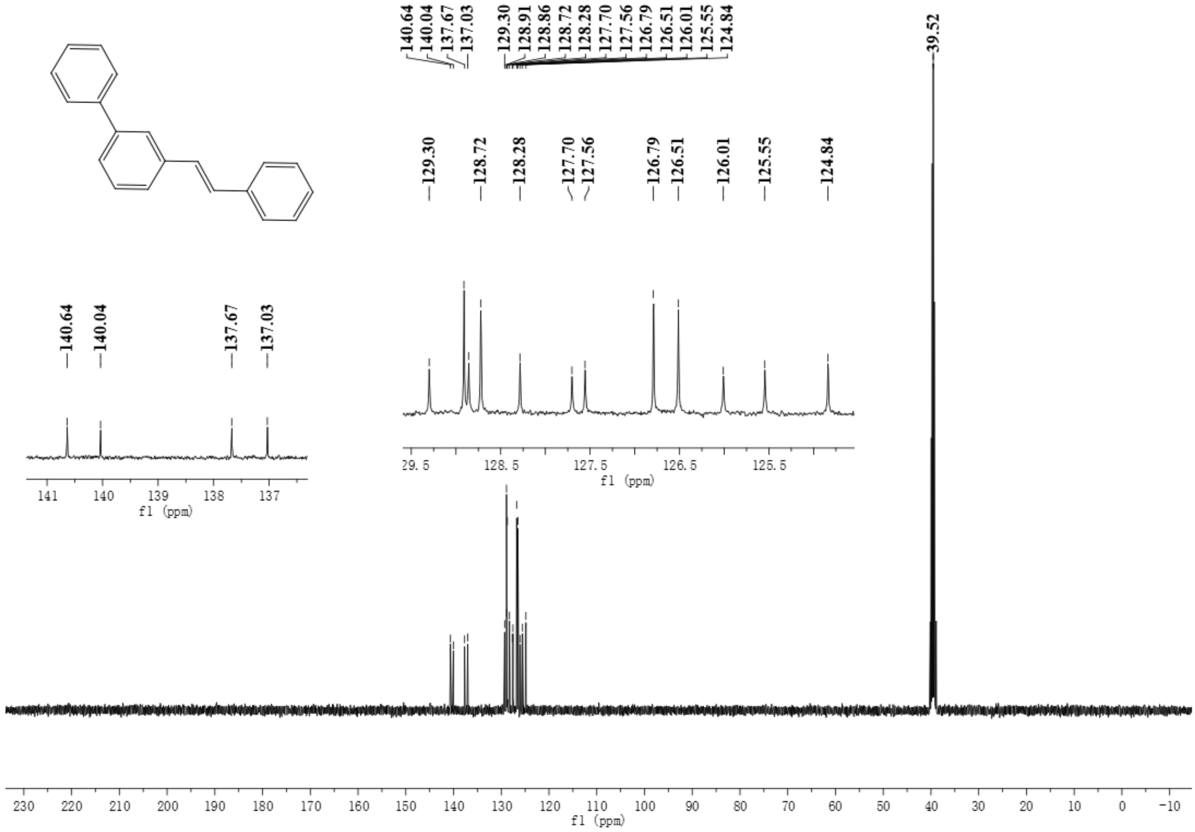

^{1}H NMR spectra of **3h** (DMSO-d_6)

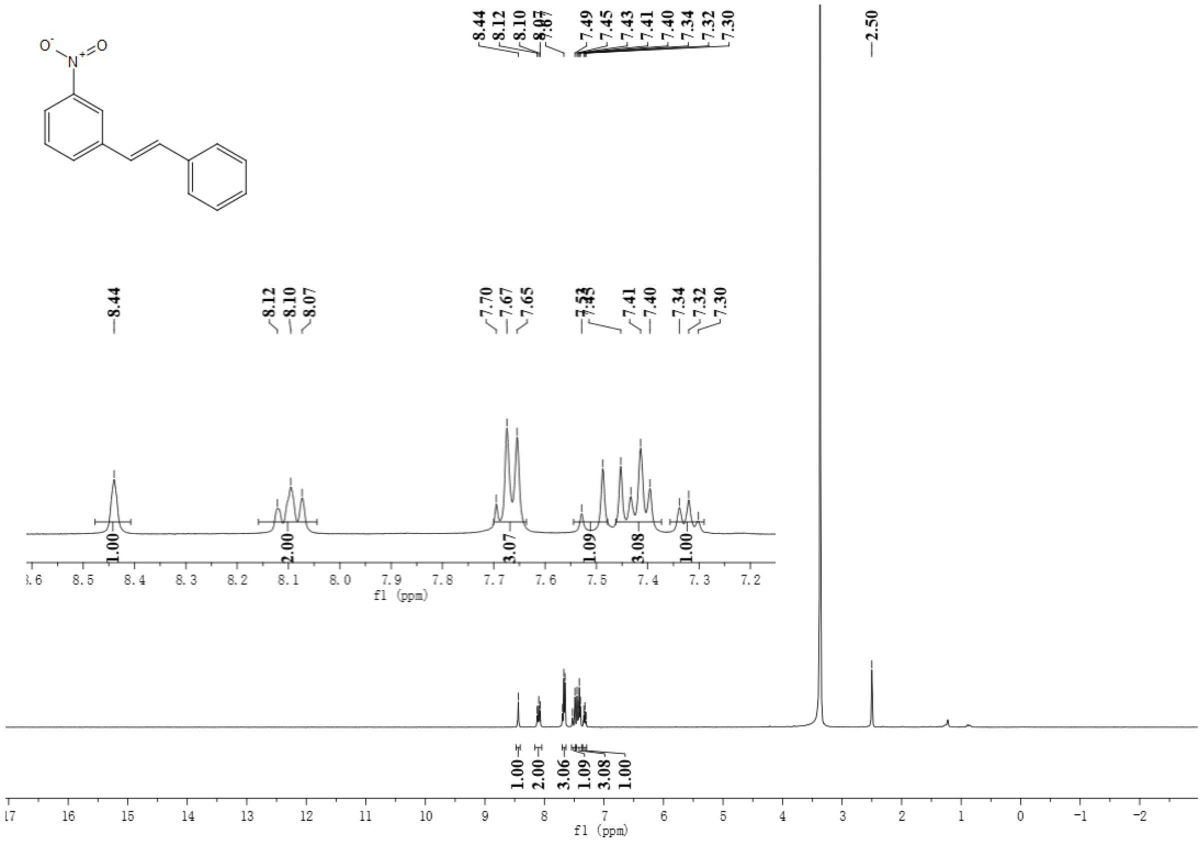

^{13}C NMR spectra of **3h** (DMSO-d_6)

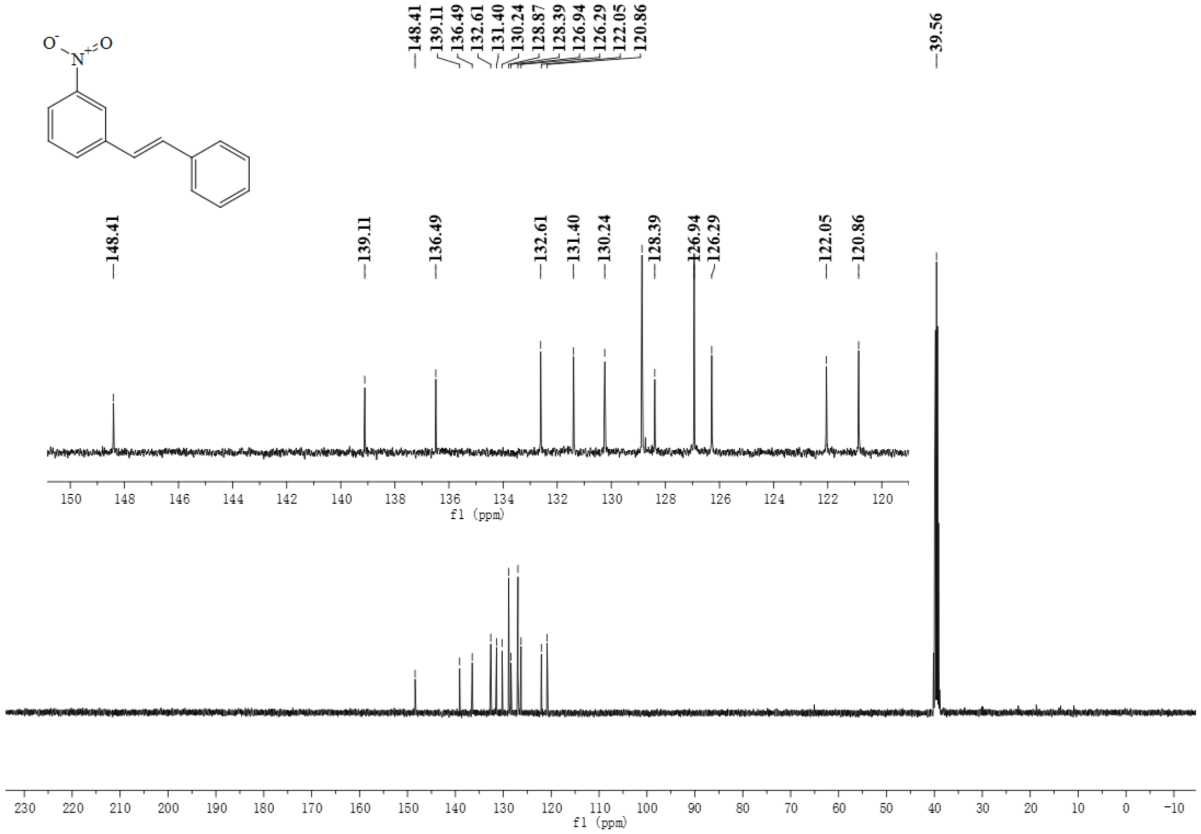

^{1}H NMR spectra of **3i** (DMSO-d_6)

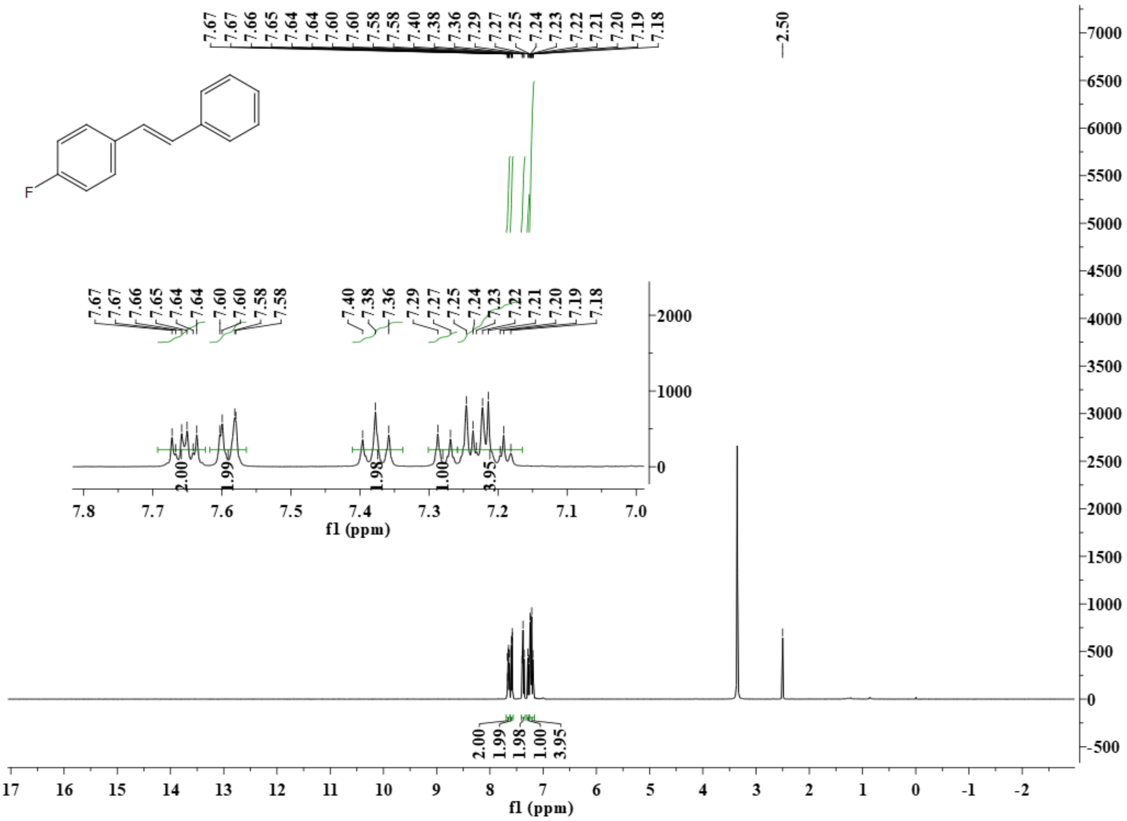

^{13}C NMR spectra of **3i** (DMSO-d_6)

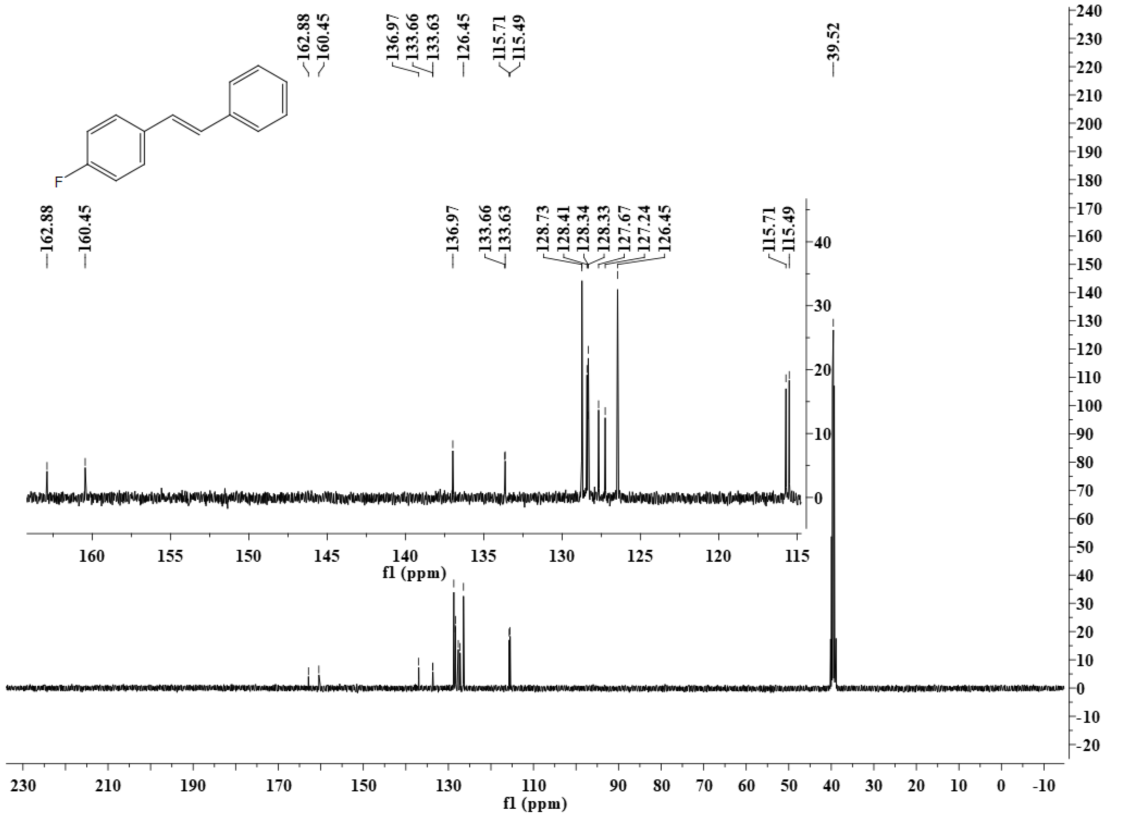

^{1}H NMR spectra of **3j** (DMSO-d_6)

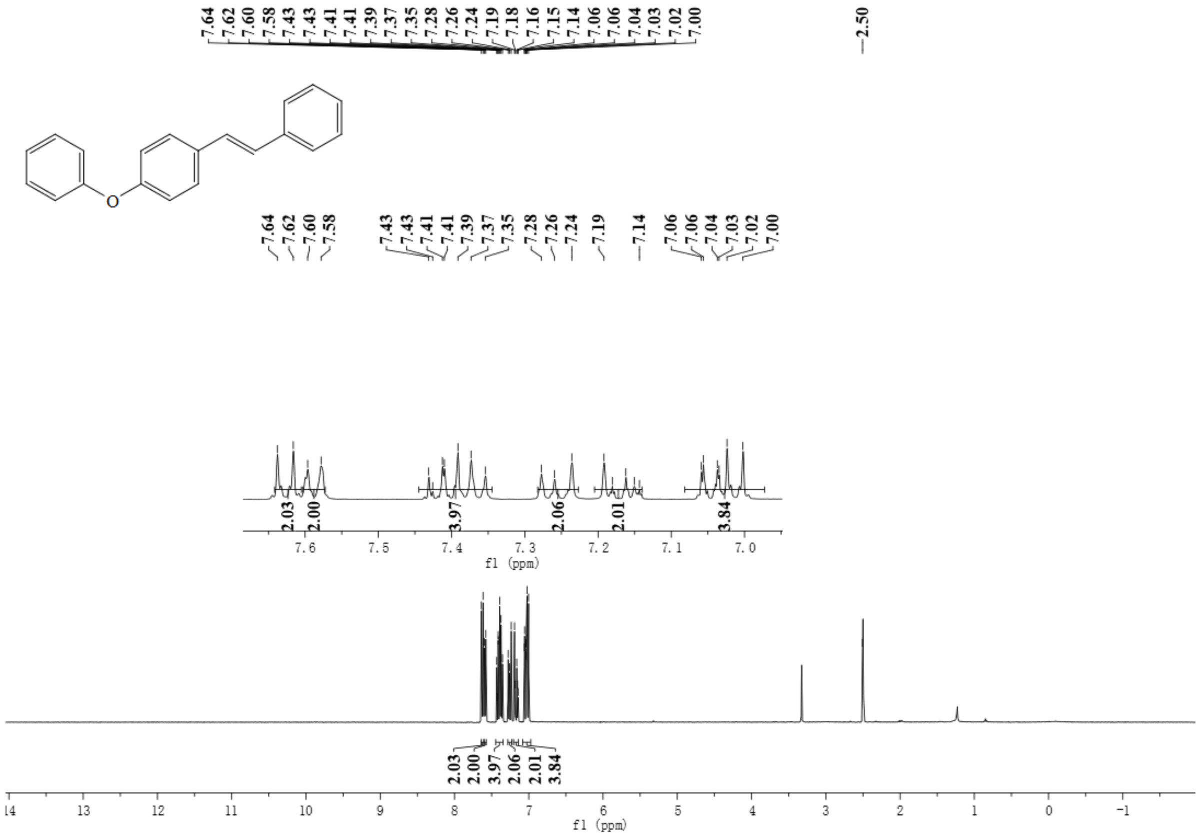

^{13}C NMR spectra of **3j** (DMSO-d_6)

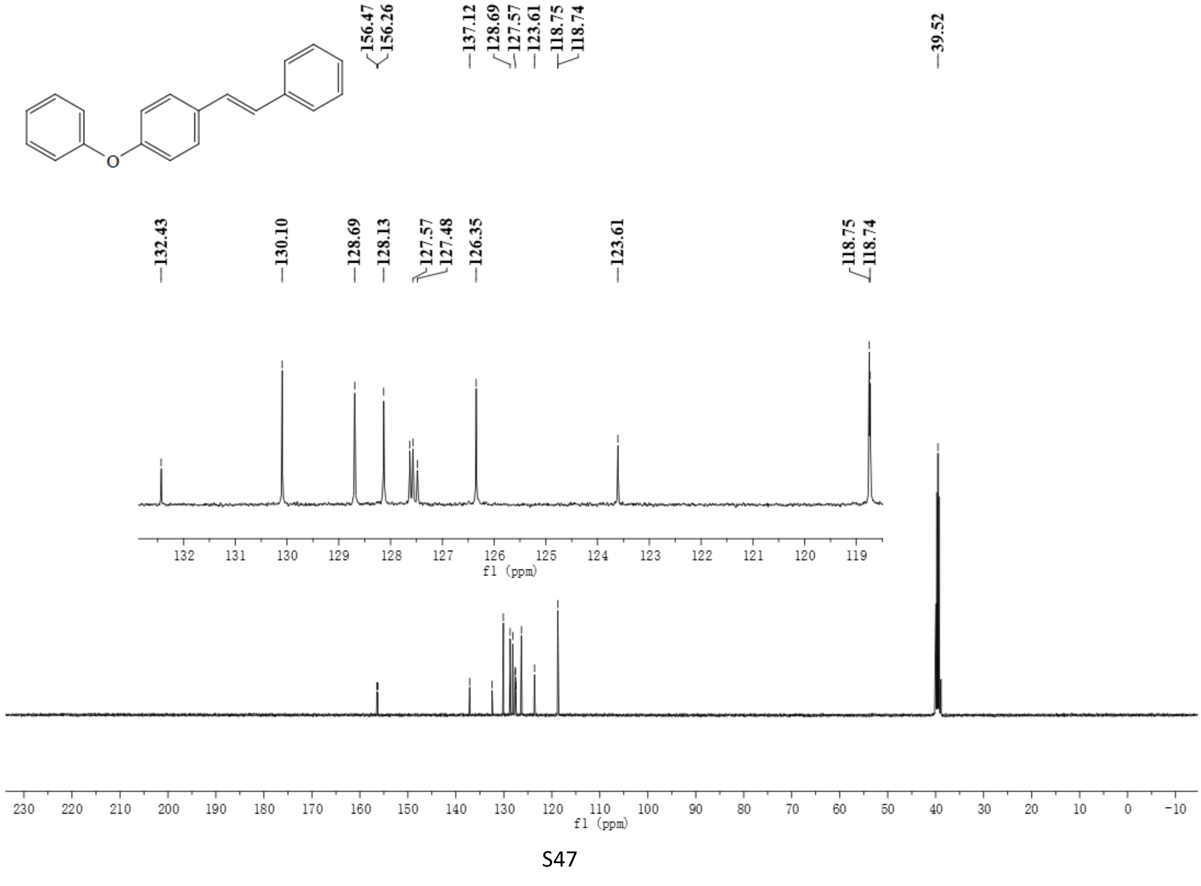

^{1}H NMR spectra of **3k** (DMSO-d_6)

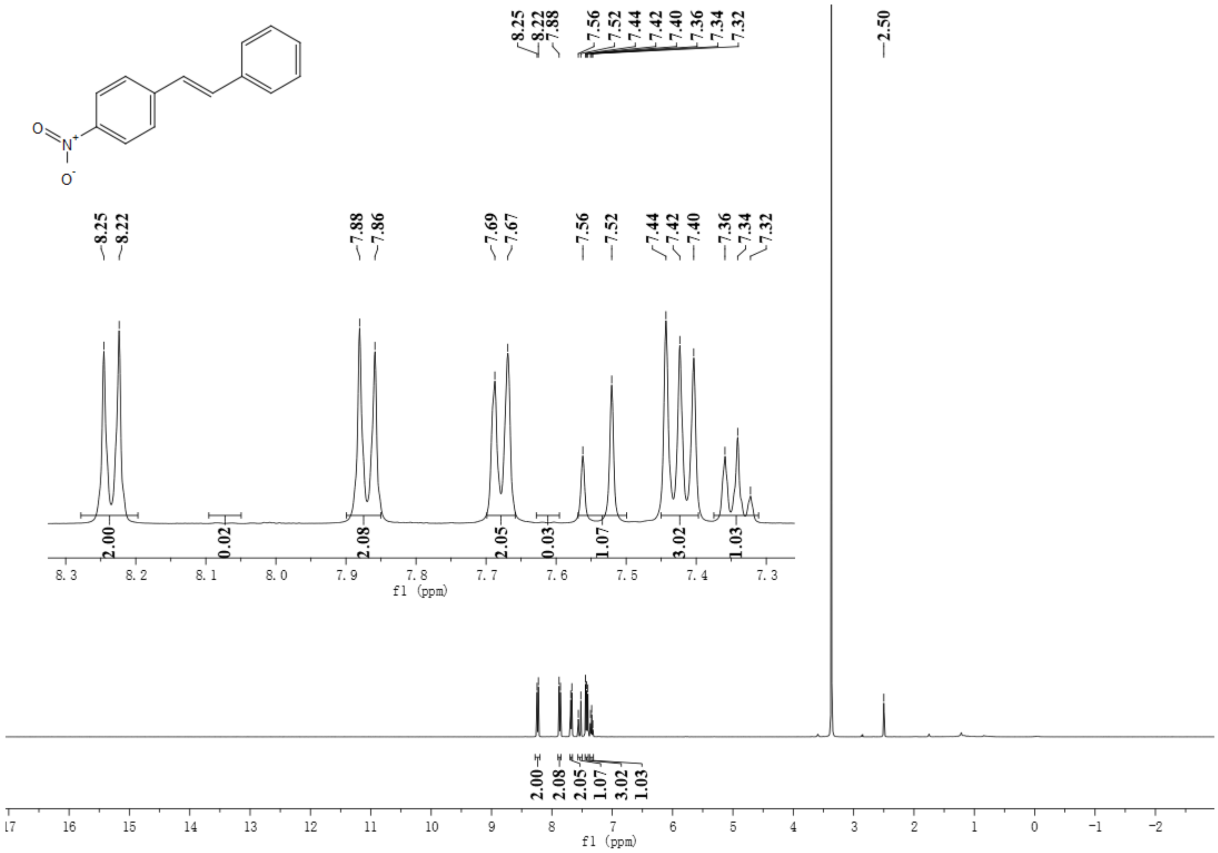

^{13}C NMR spectra of **3k** (DMSO-d_6)

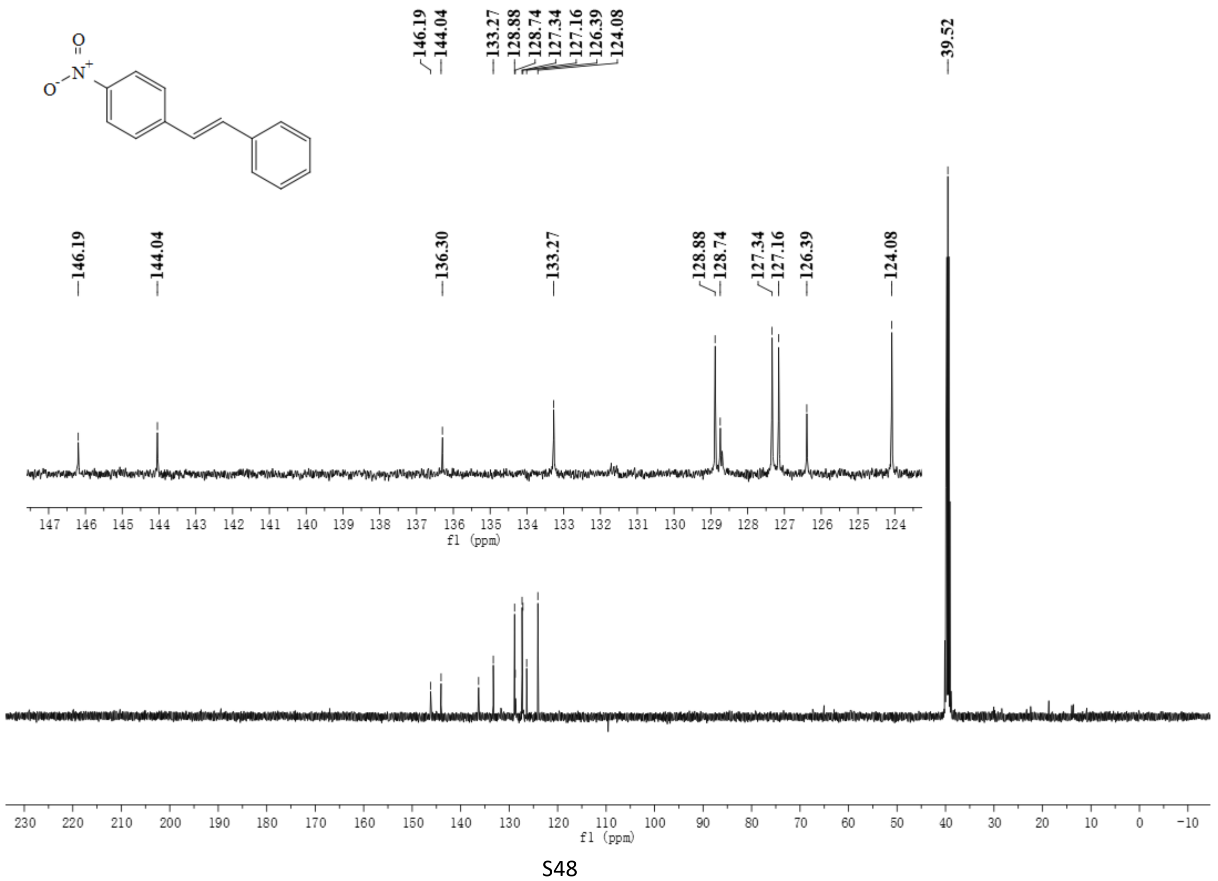

^{1}H NMR spectra of **3l** (DMSO-d_6)

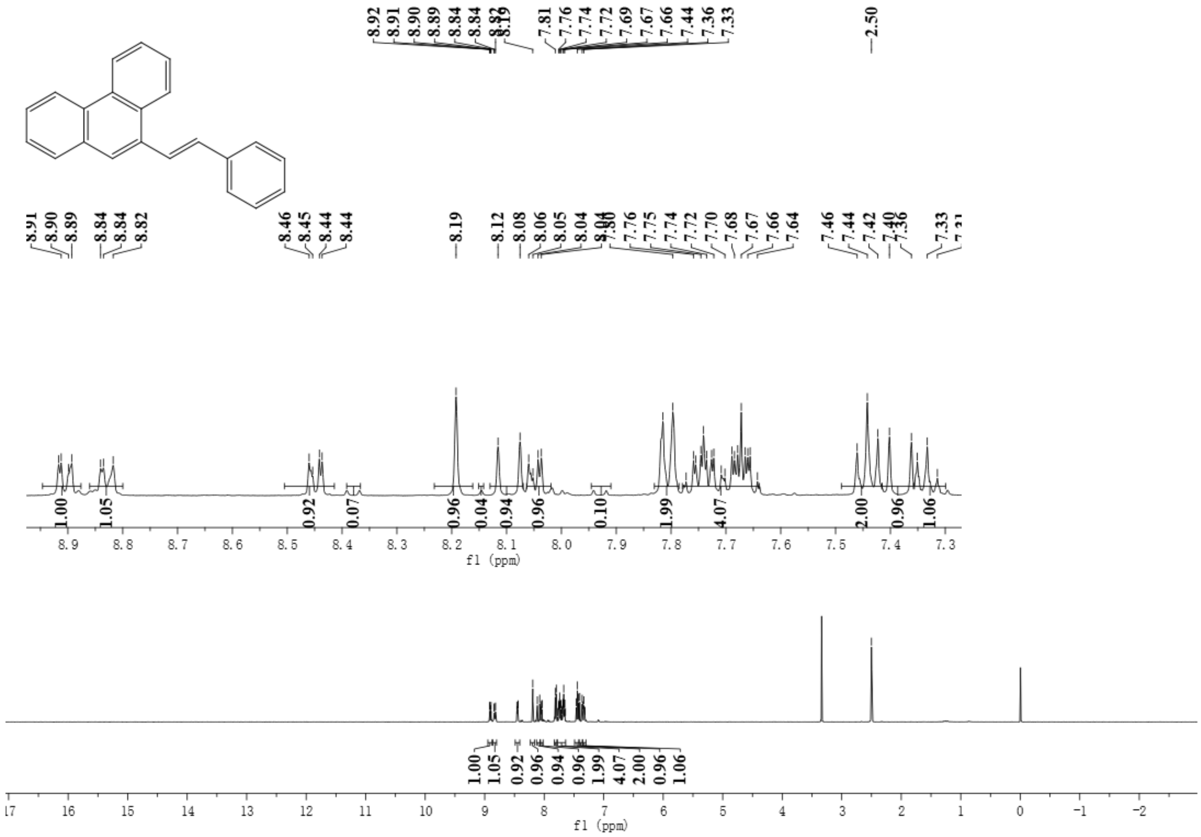

^{13}C NMR spectra of **3l** (DMSO-d_6)

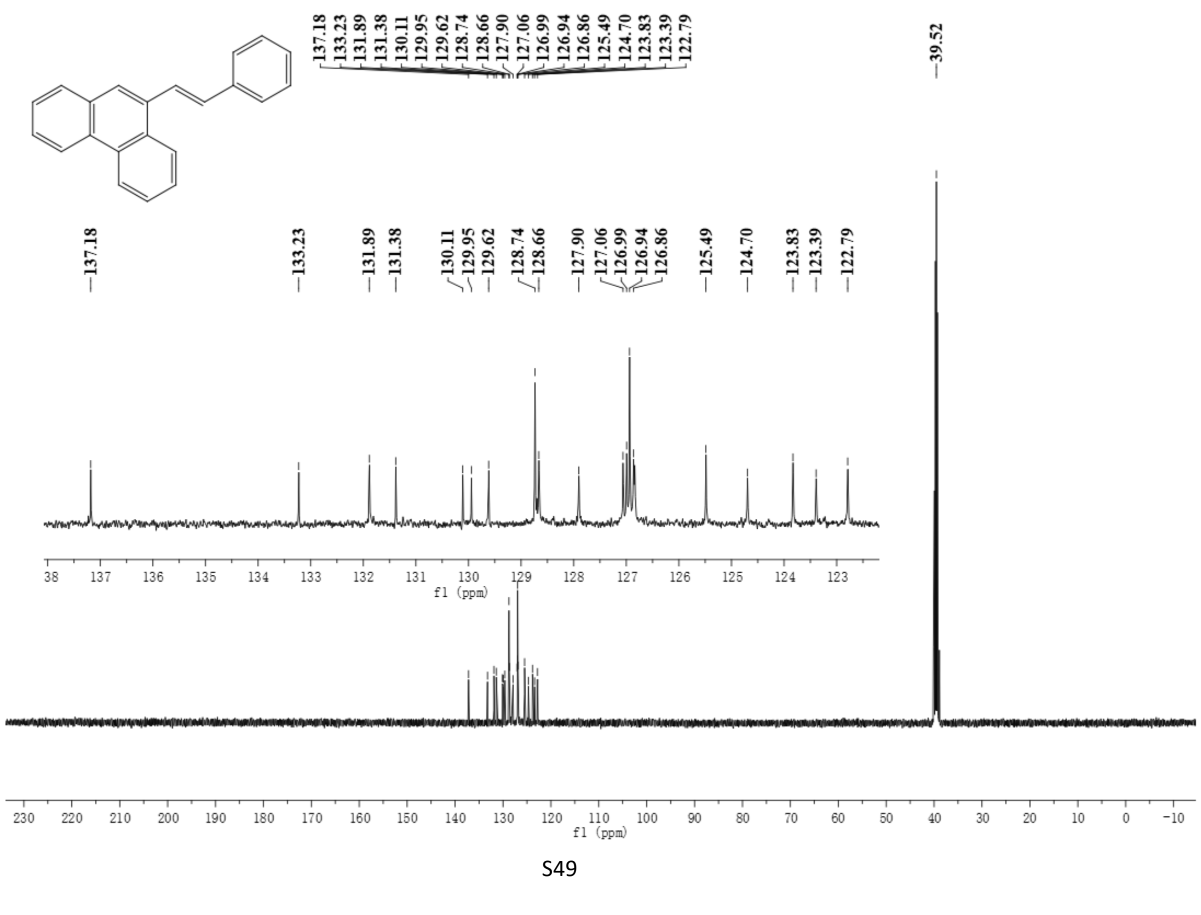

^{1}H NMR spectra of **3m** (DMSO-d_6)

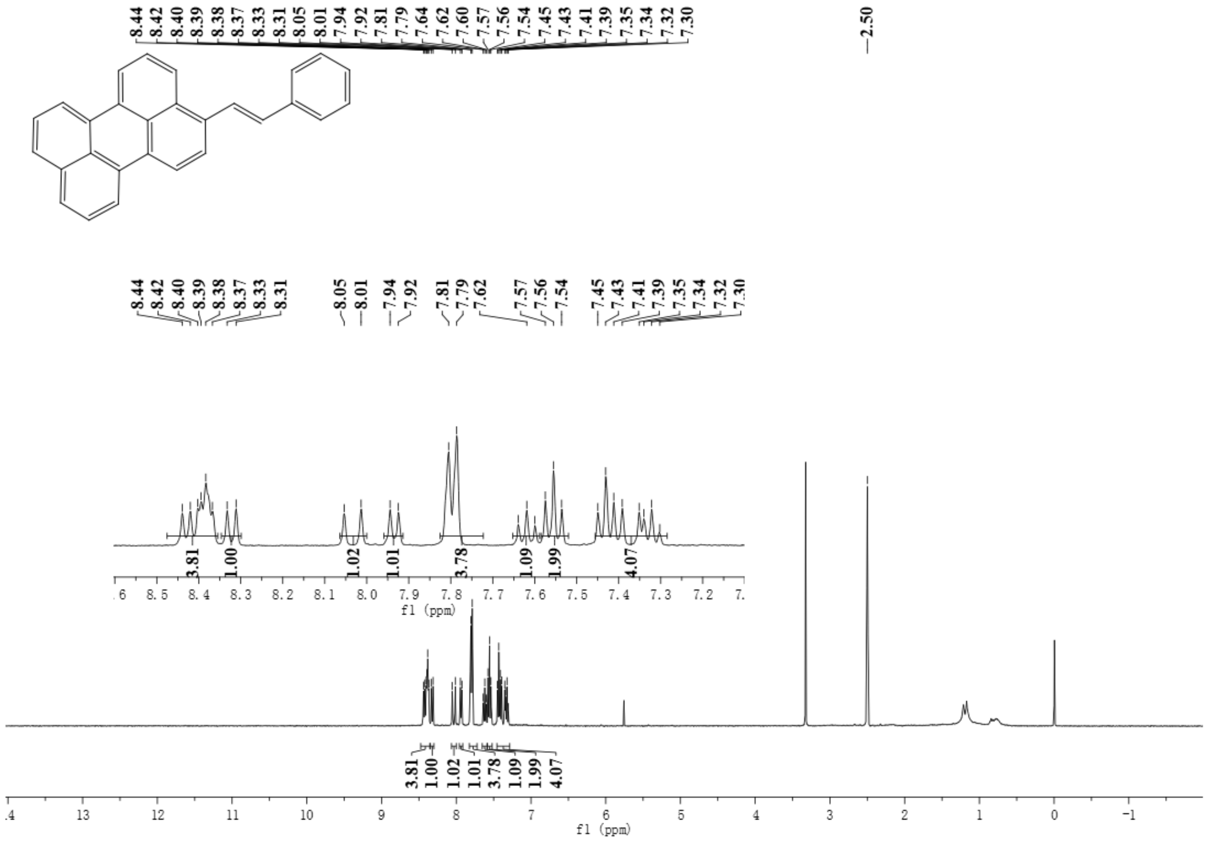

^{13}C NMR spectra of **3m** (DMSO-d_6)

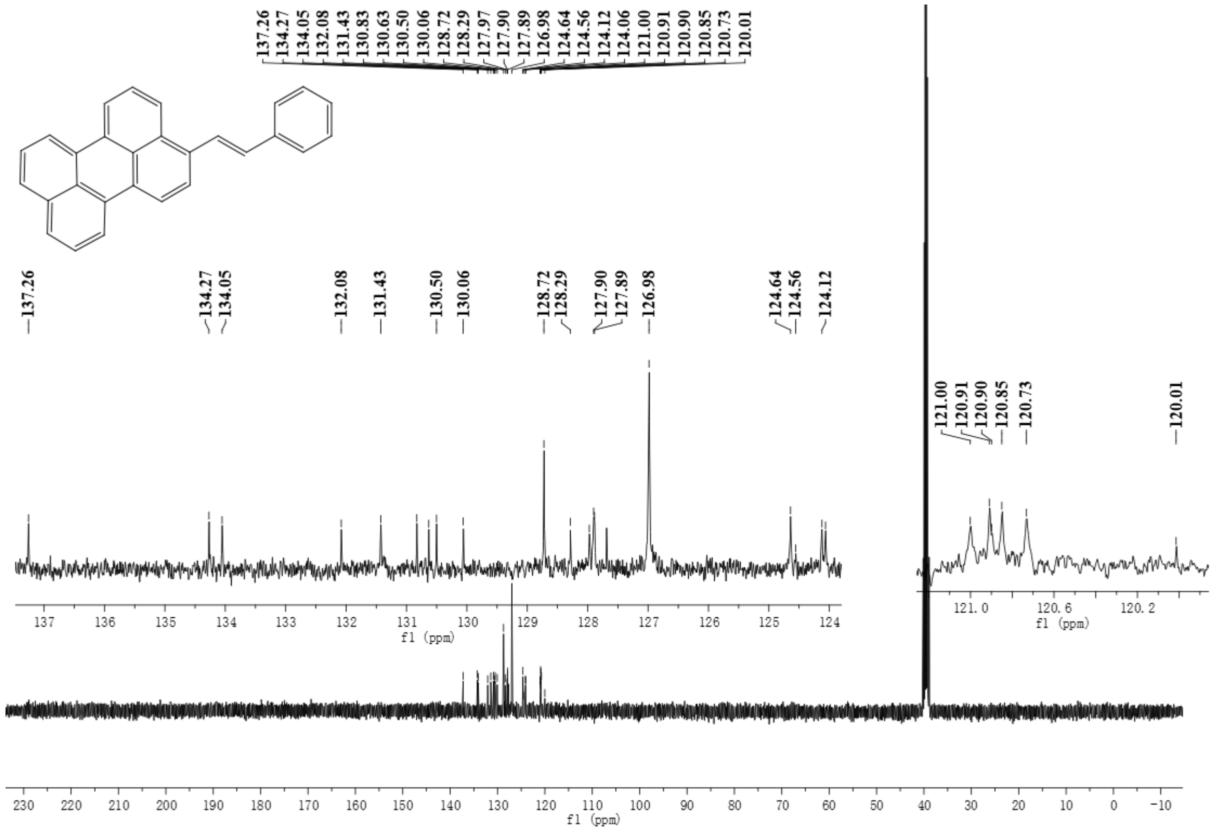

^{1}H NMR spectra of **3n** (DMSO-d_6)

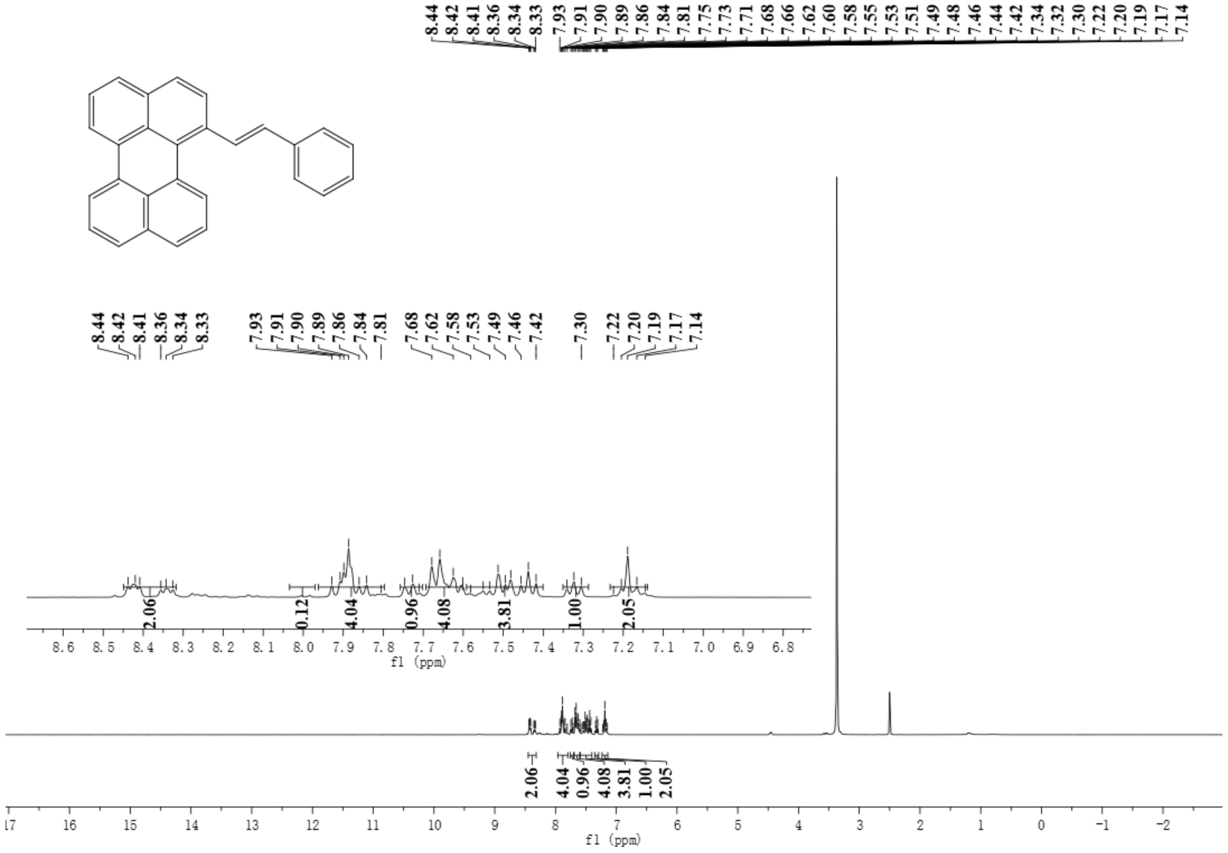

^{13}C NMR spectra of **3n** (DMSO-d_6)

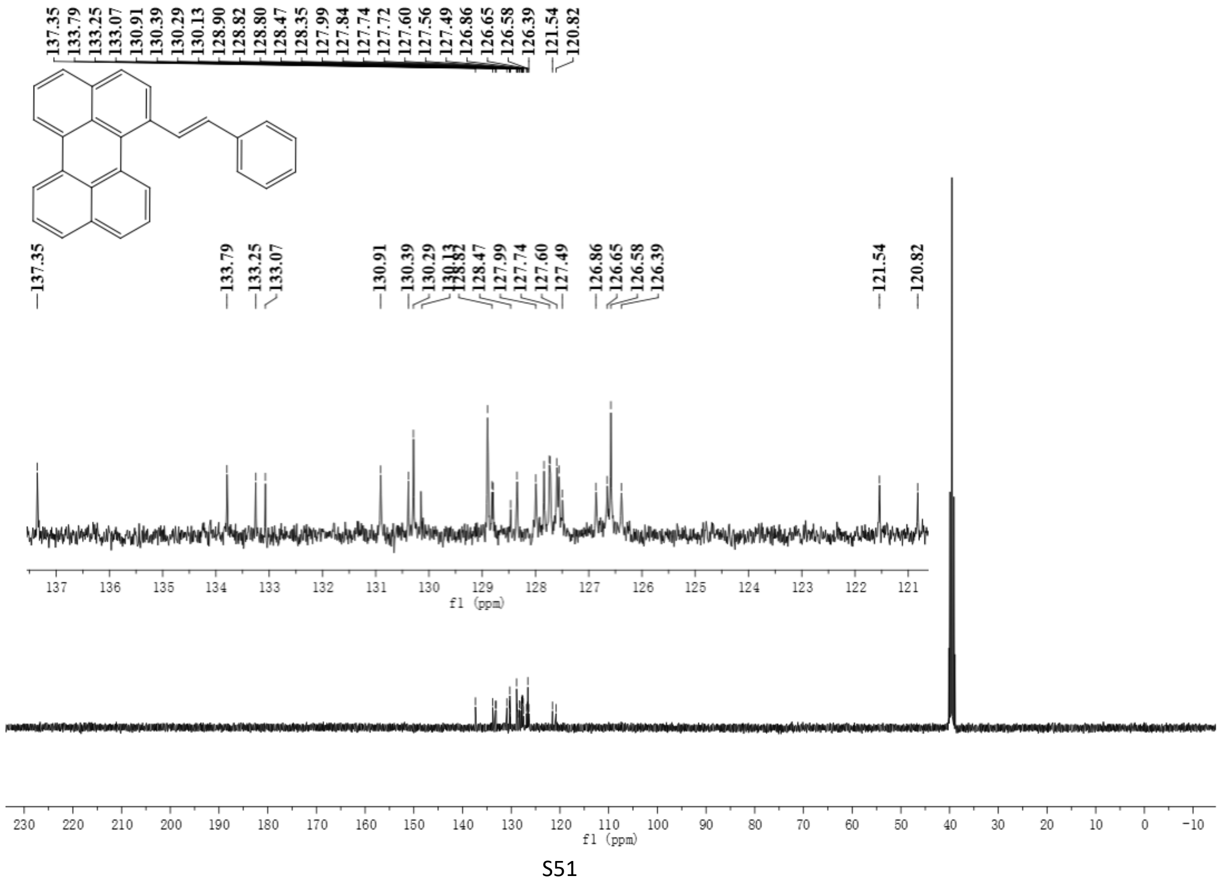

^{1}H NMR spectra of **4a** (DMSO-d_6)

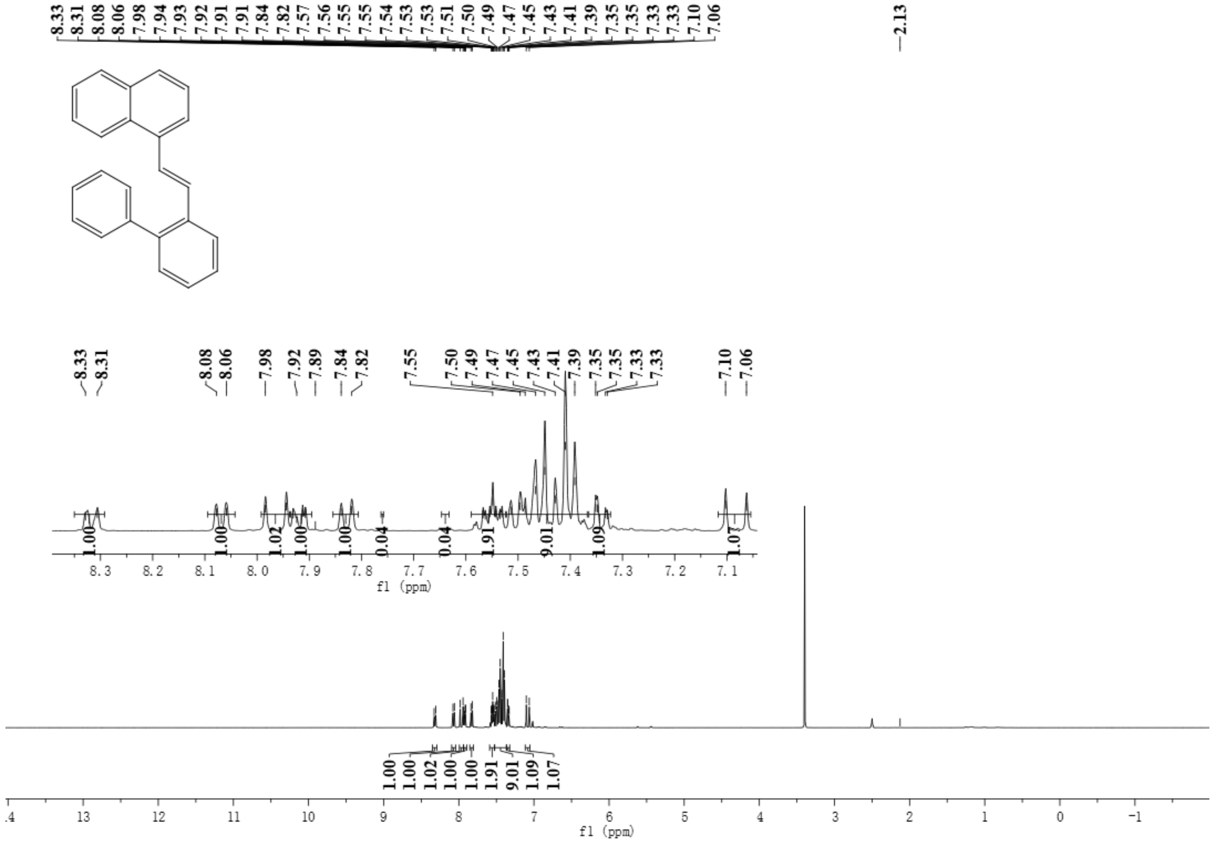

^{13}C NMR spectra of **4a** (DMSO-d_6)

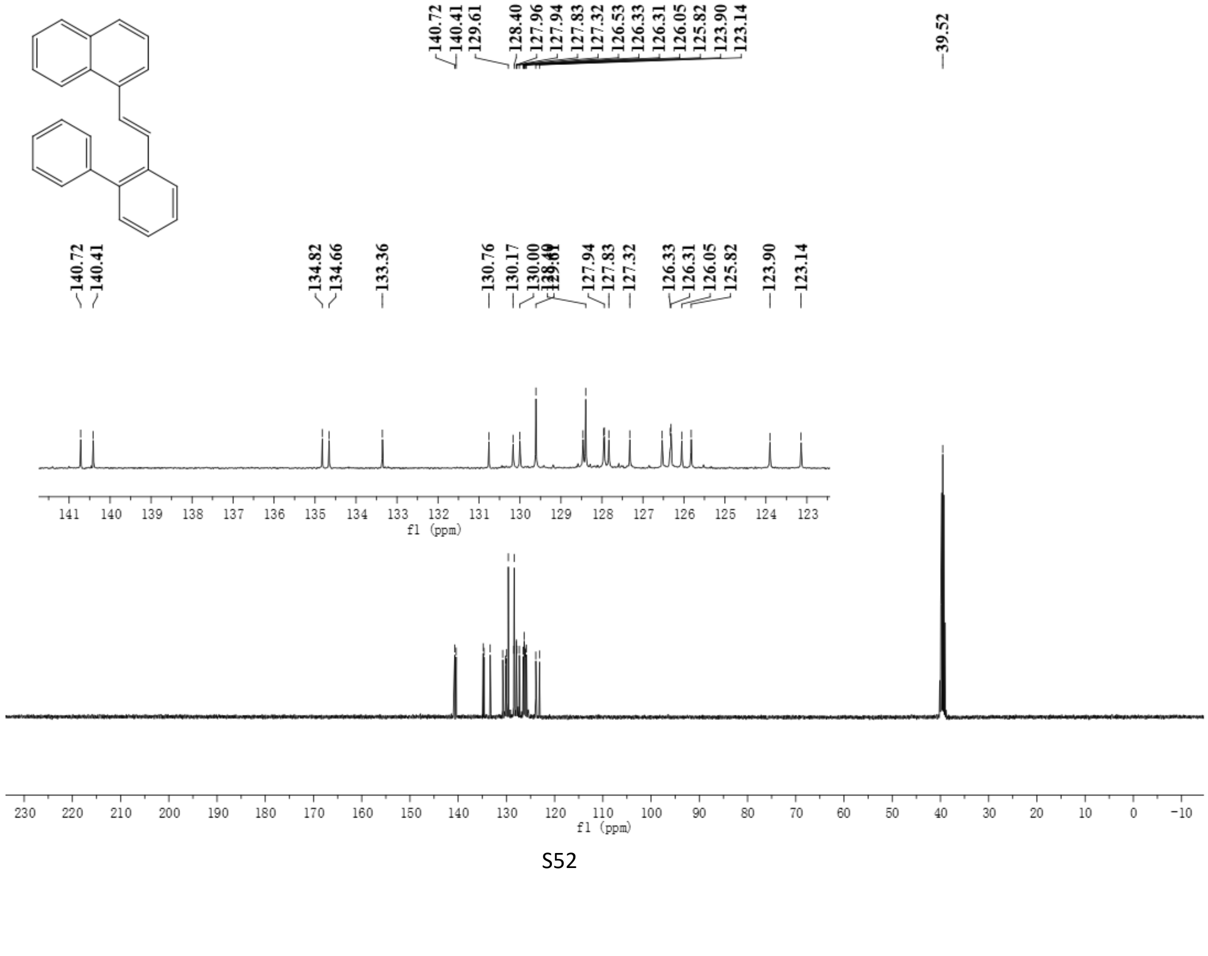

^{1}H NMR spectra of **4b** (DMSO-d_6)

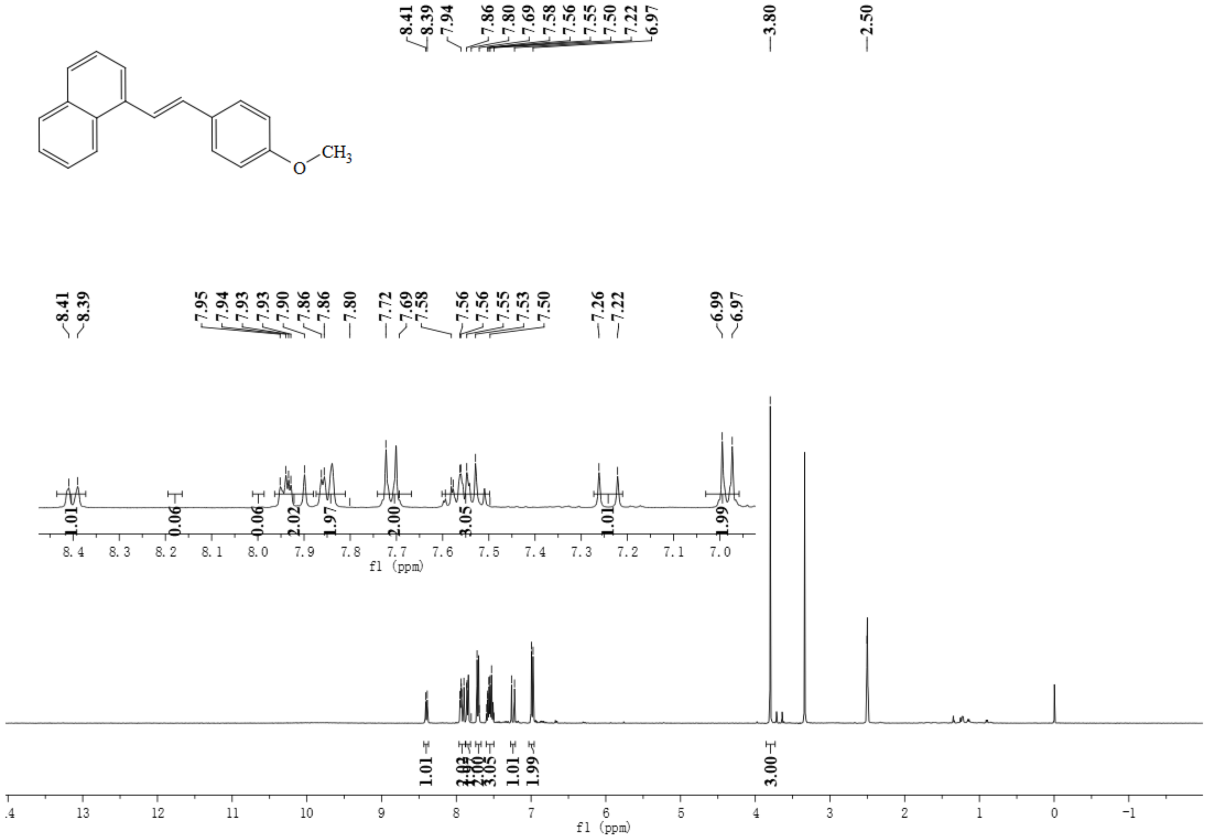

^{13}C NMR spectra of **4b** (DMSO-d_6)

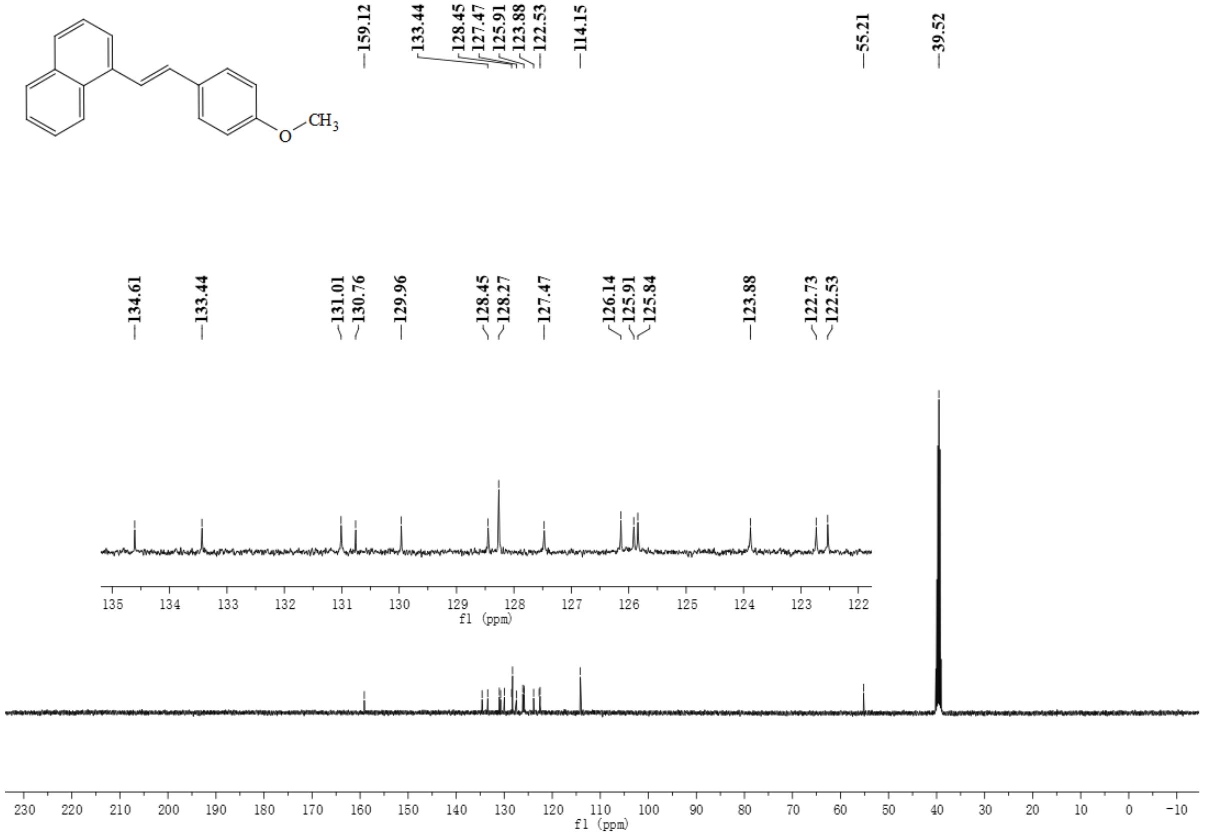

^{1}H NMR spectra of **4c** (DMSO-d_6)

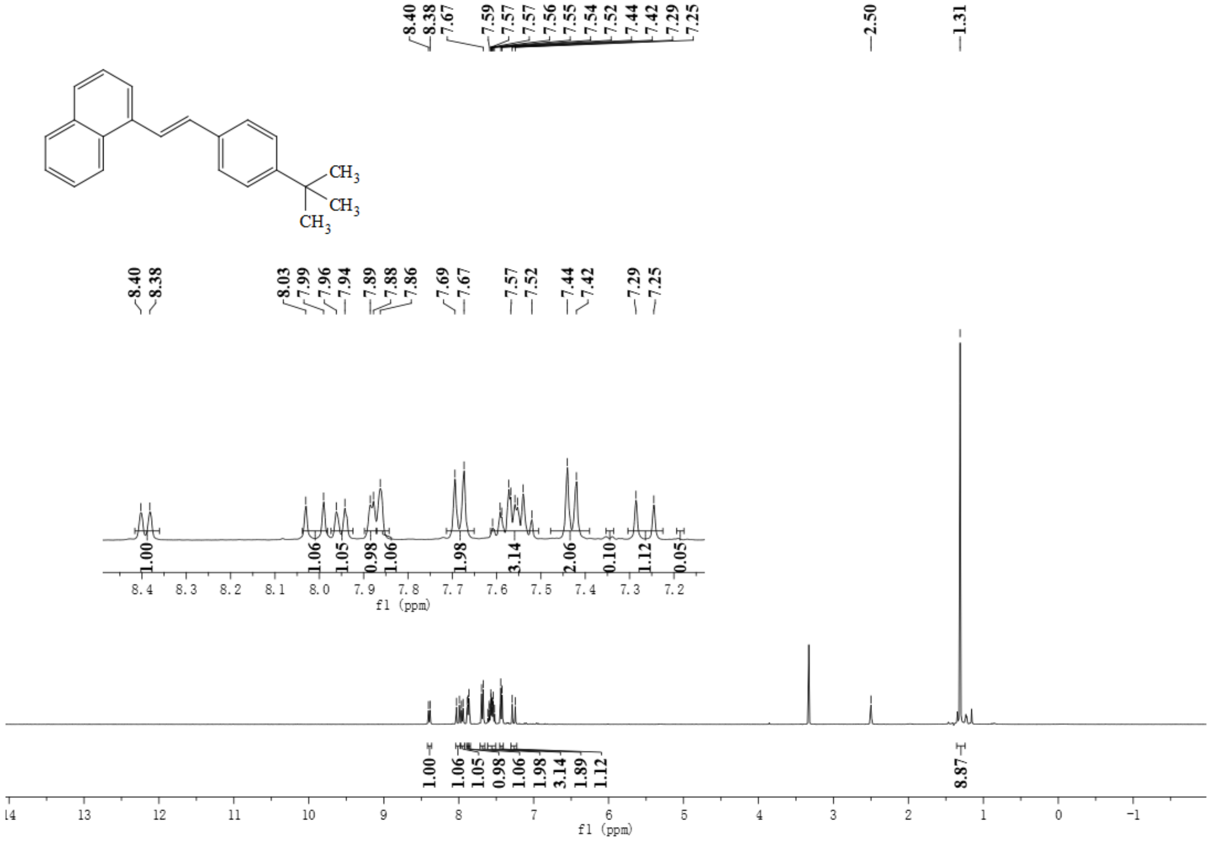

^{13}C NMR spectra of **4c** (DMSO-d_6)

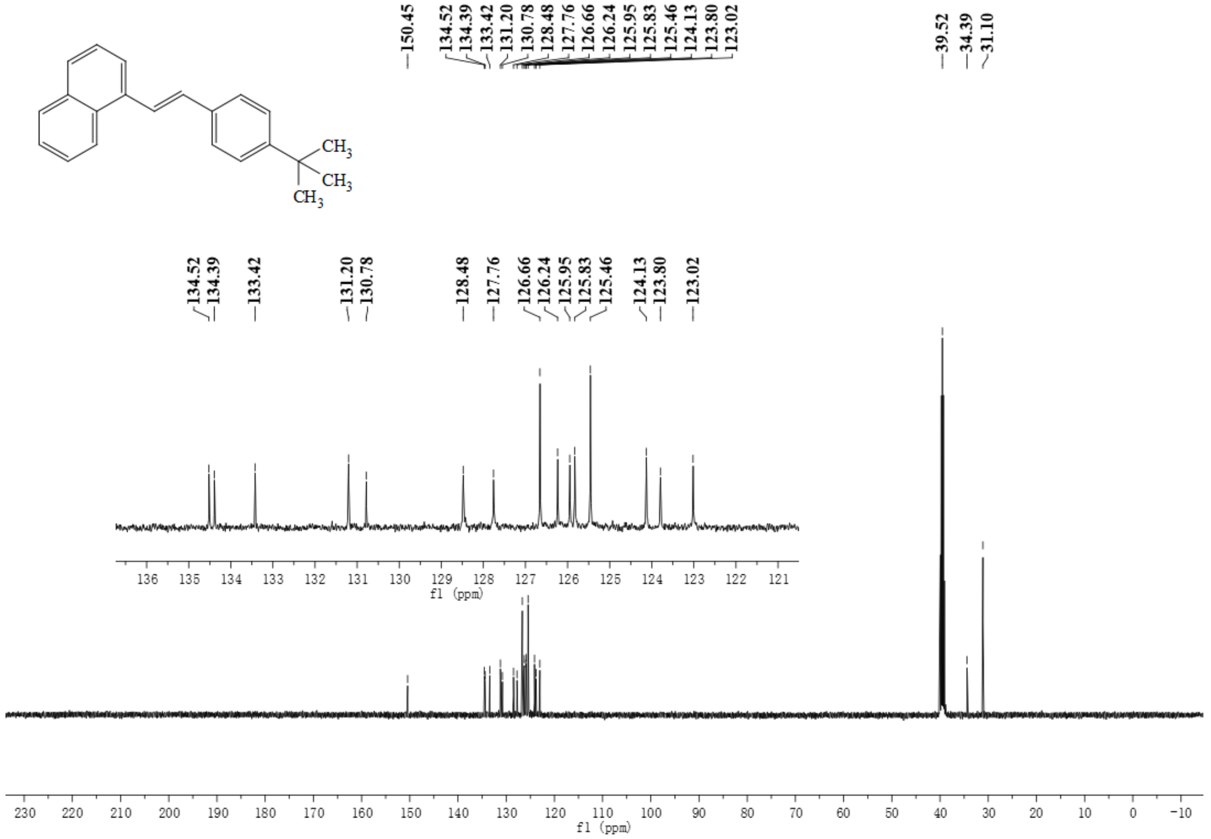

^{1}H NMR spectra of **4d** (DMSO-d_6)

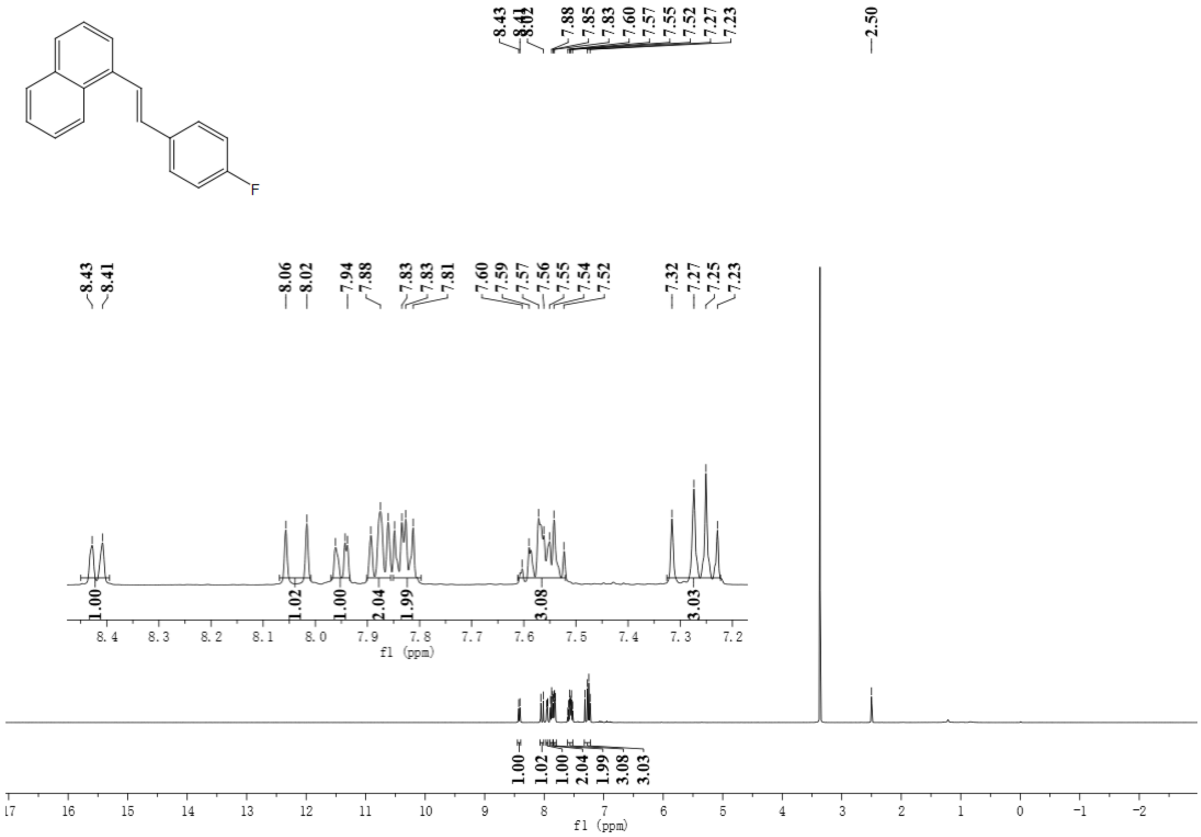

^{13}C NMR spectra of **4d** (DMSO-d_6)

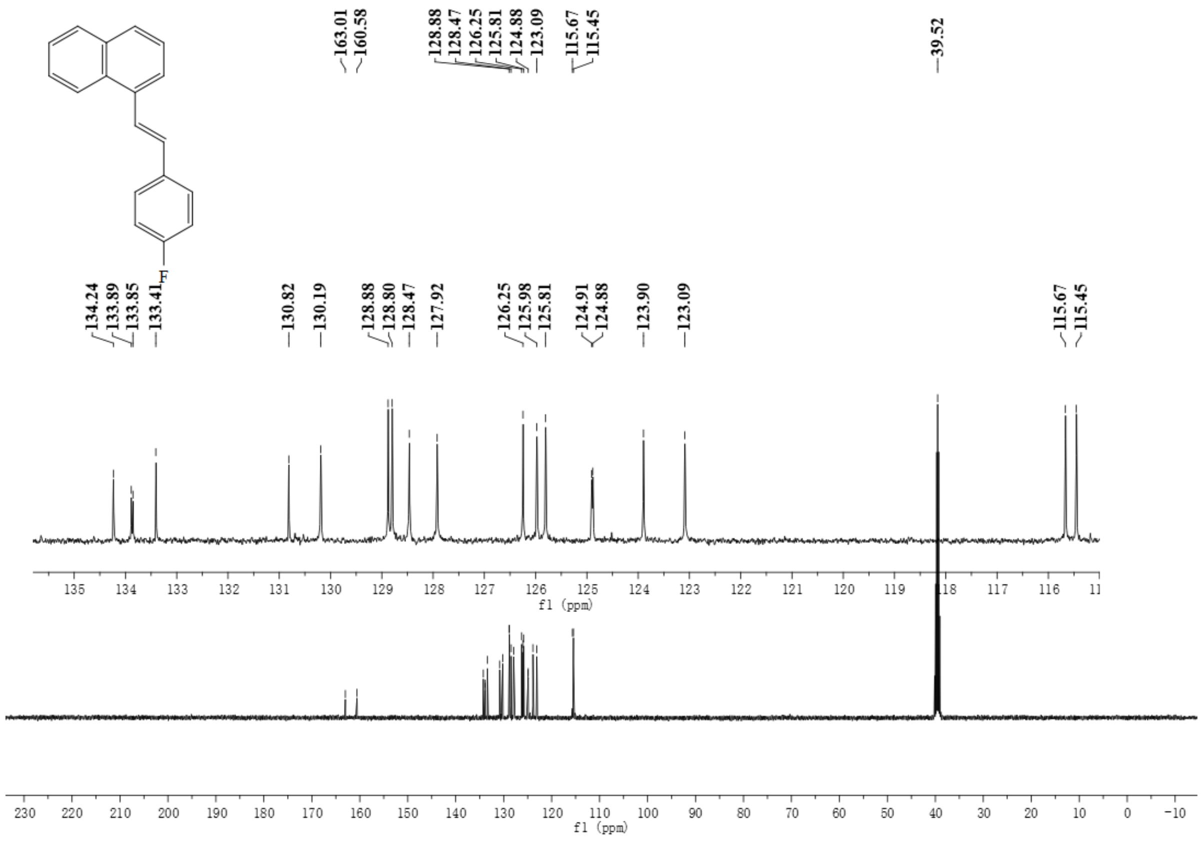

^{1}H NMR spectra of **4e** (DMSO-d_6)

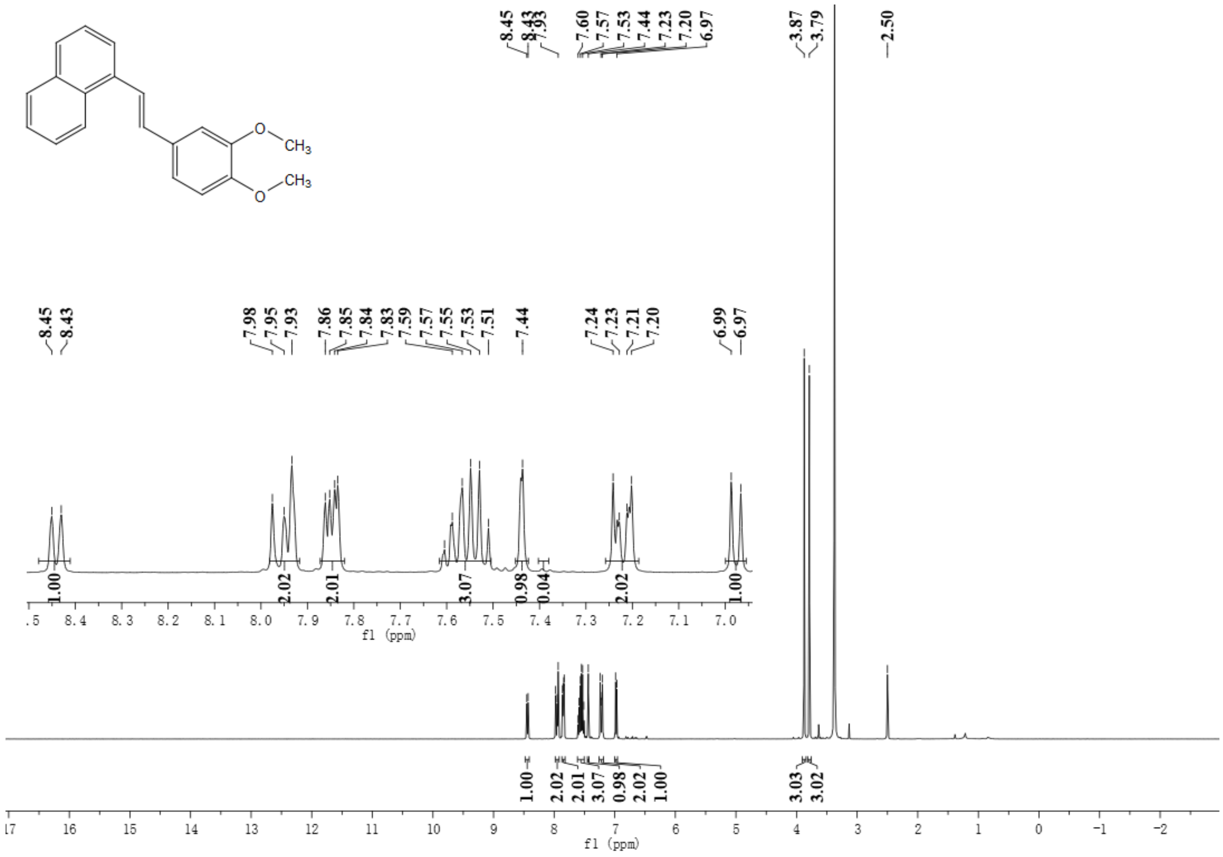

^{13}C NMR spectra of **4e** (DMSO-d_6)

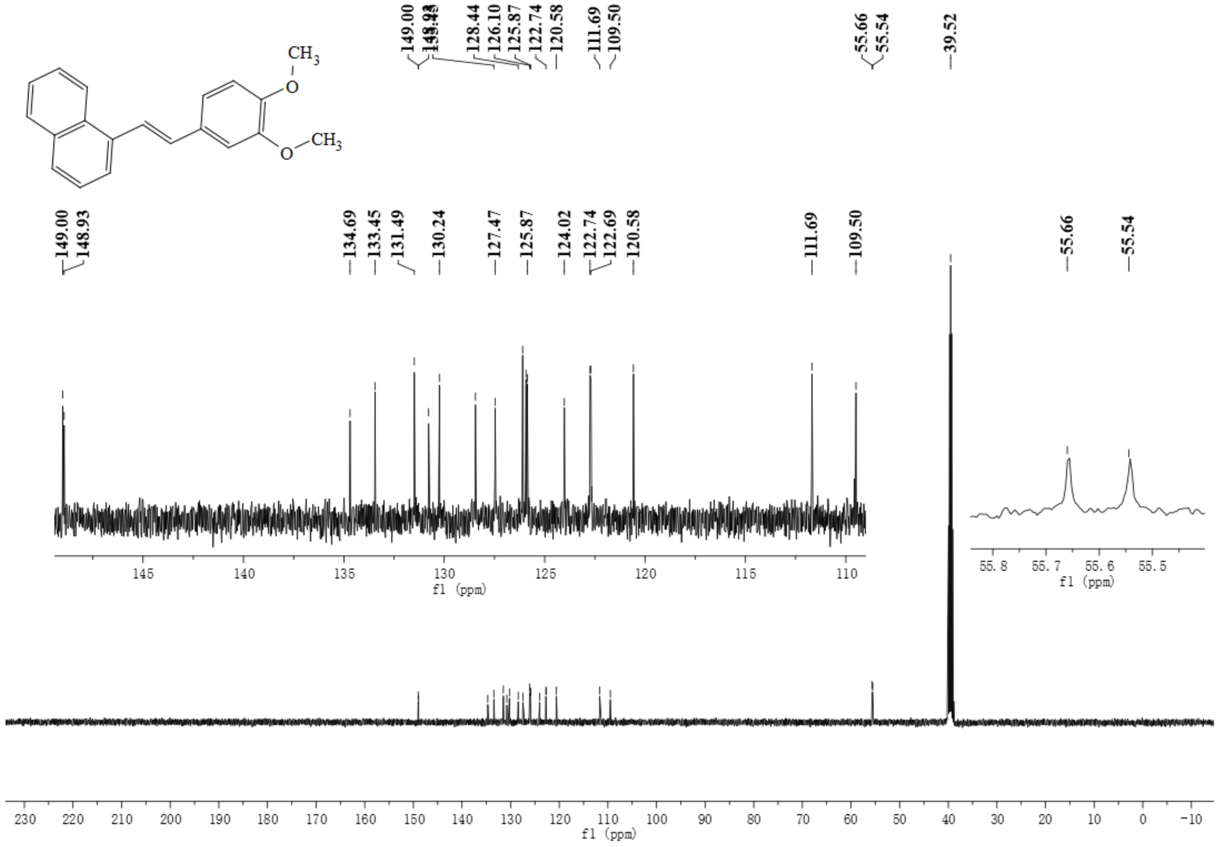

^{1}H NMR spectra of **4f** (DMSO-d_6)

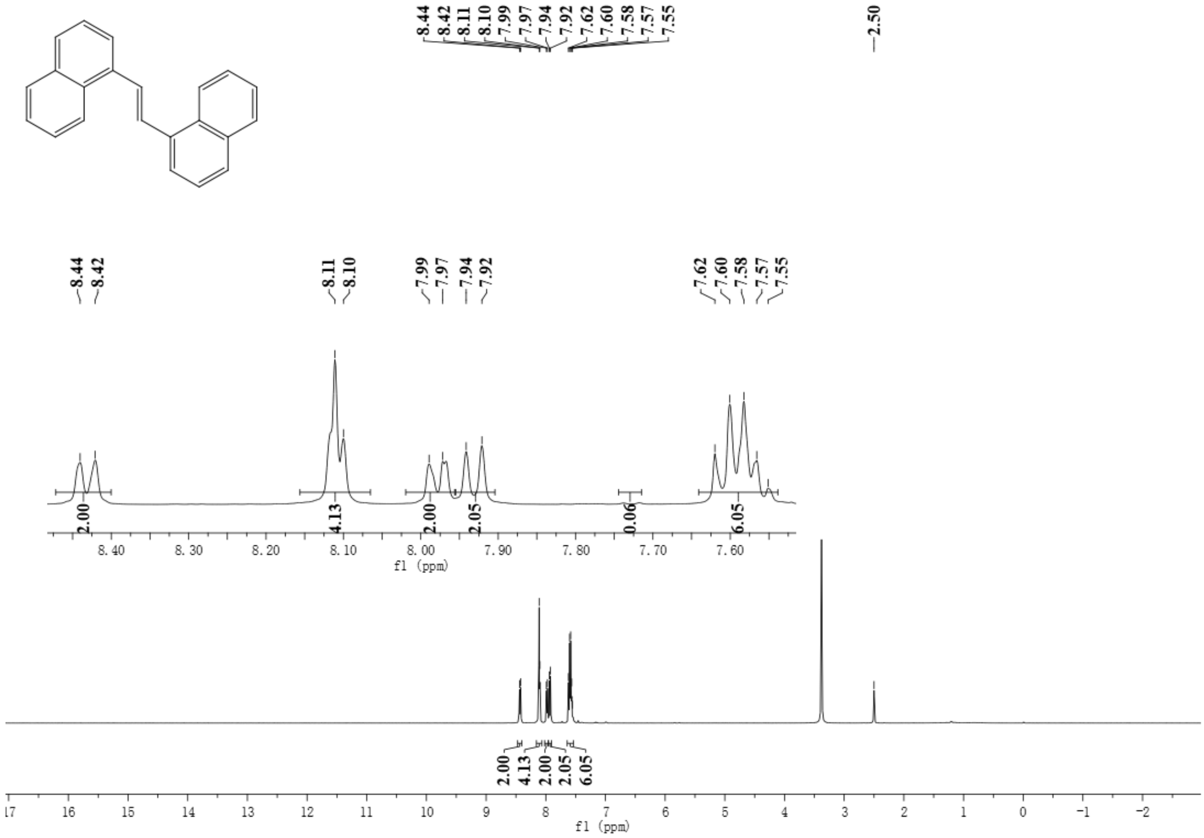

^{13}C NMR spectra of **4f** (DMSO-d_6)

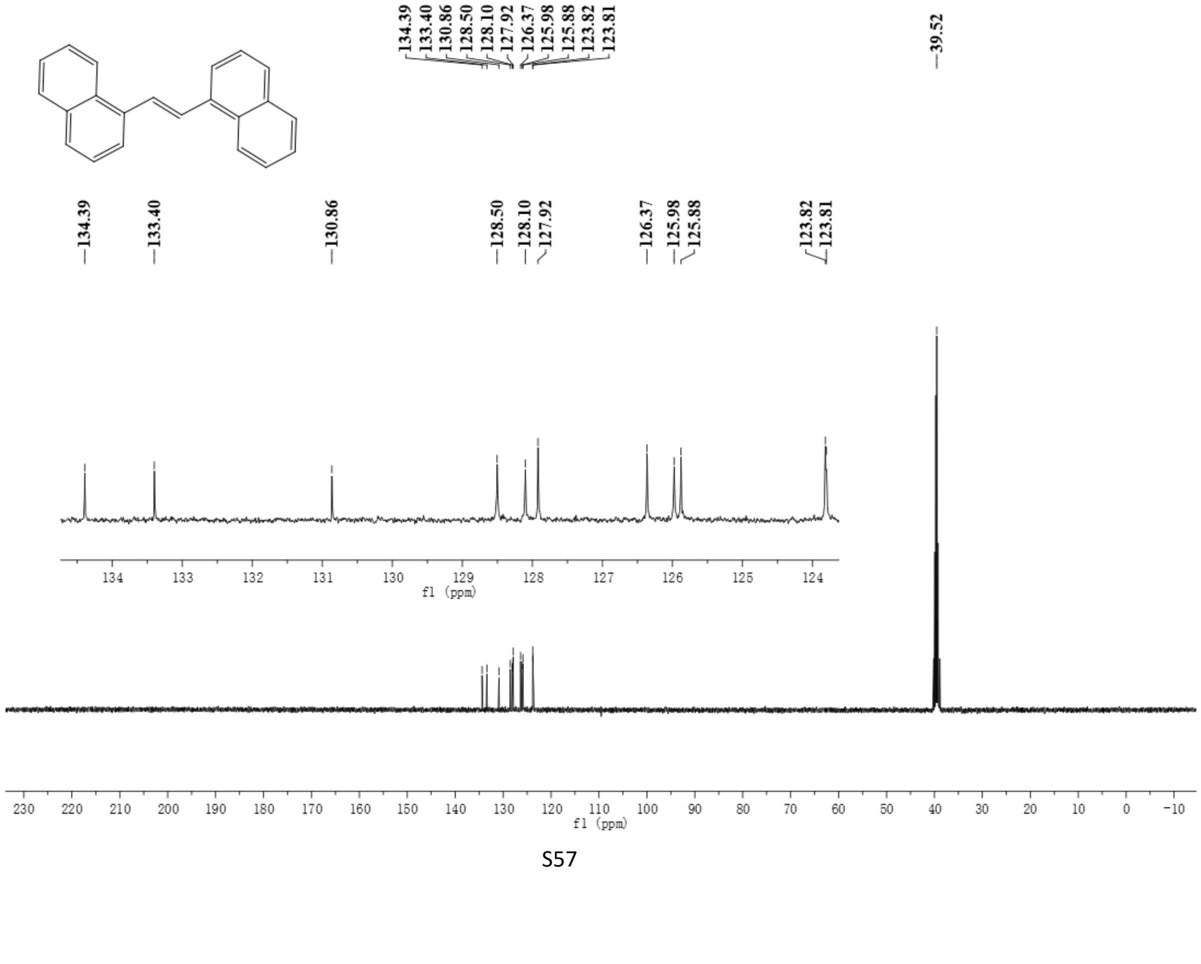

^{1}H NMR spectra of **4g** (DMSO-d_6)

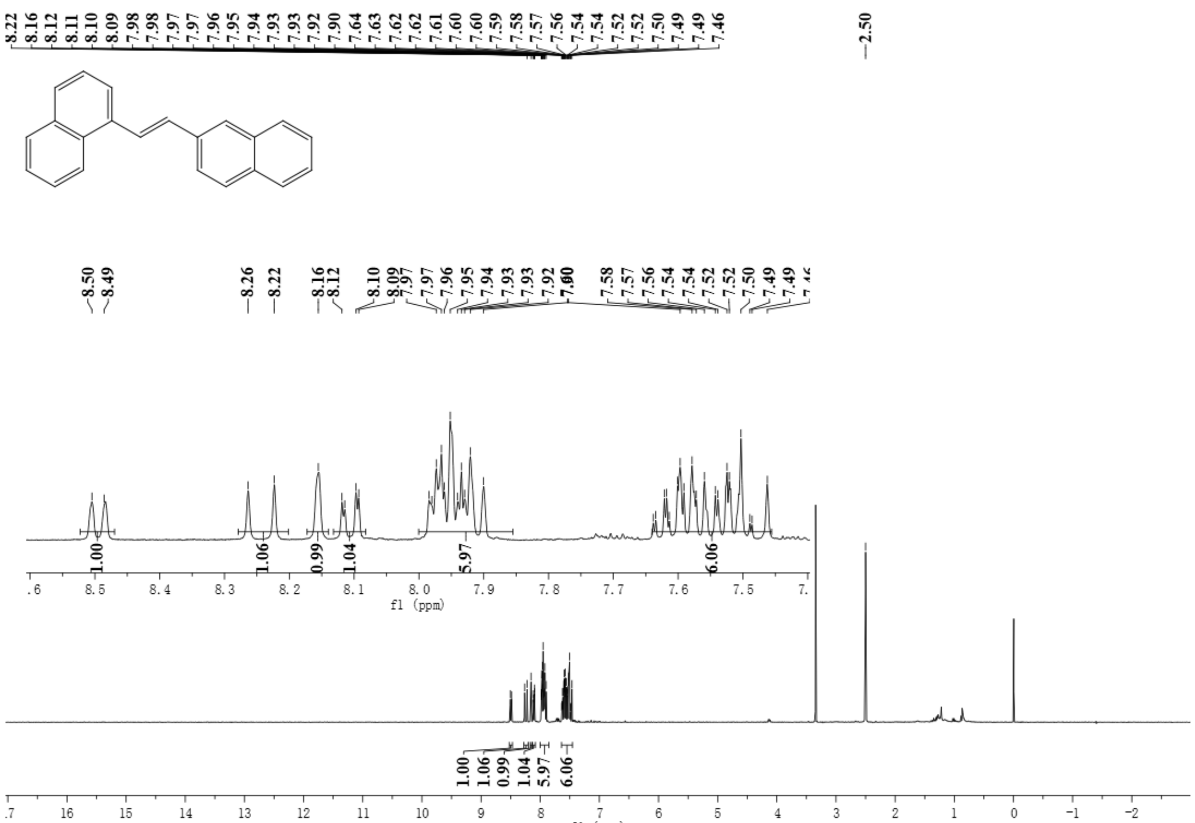

^{13}C NMR spectra of **4g** (DMSO-d_6)

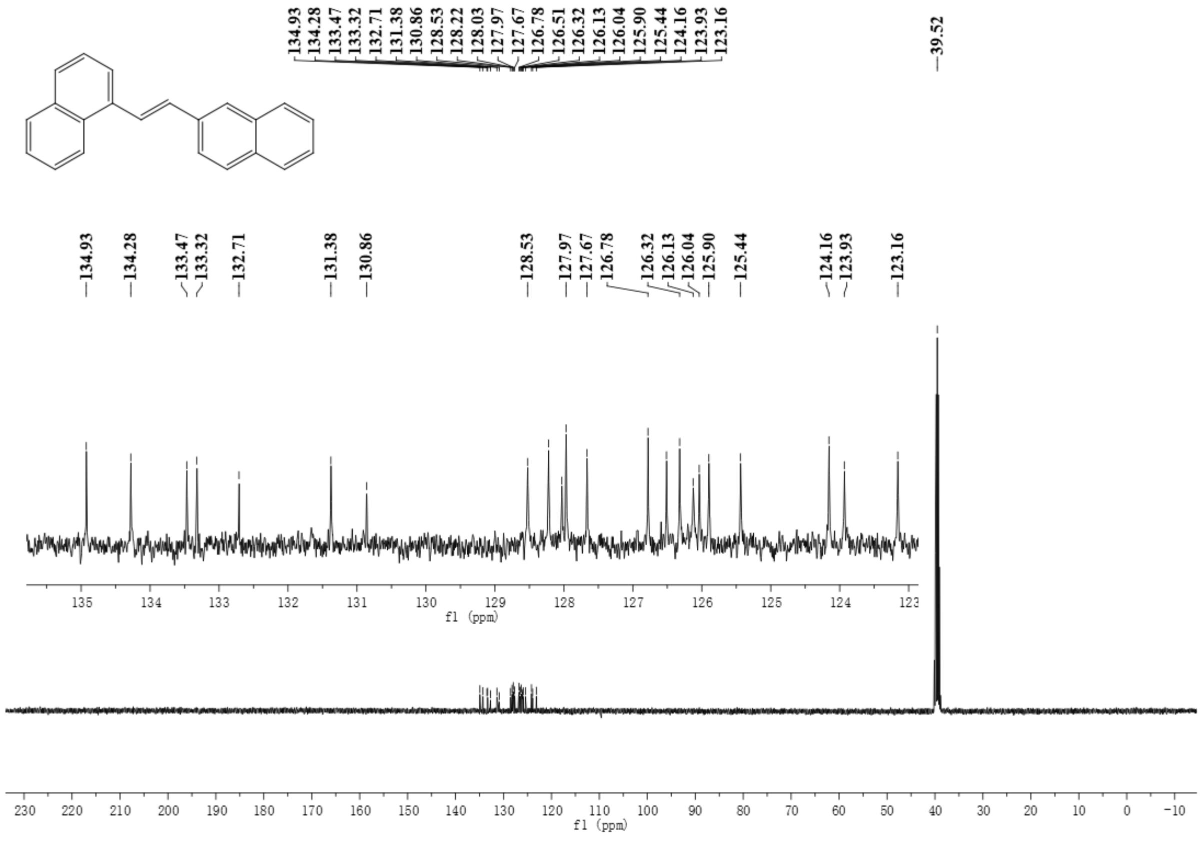

^{1}H NMR spectra of **4h** (DMSO-d_6)

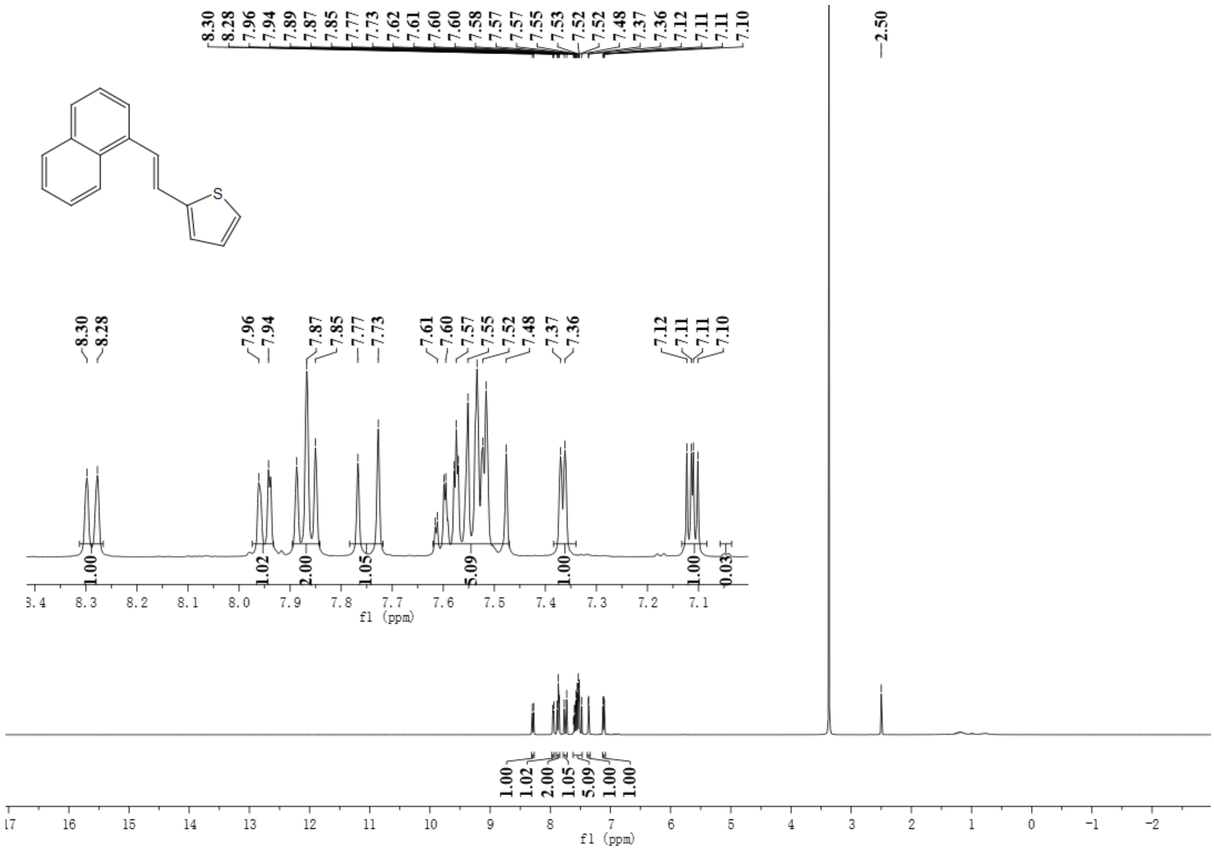

^{13}C NMR spectra of **4h** (DMSO-d_6)

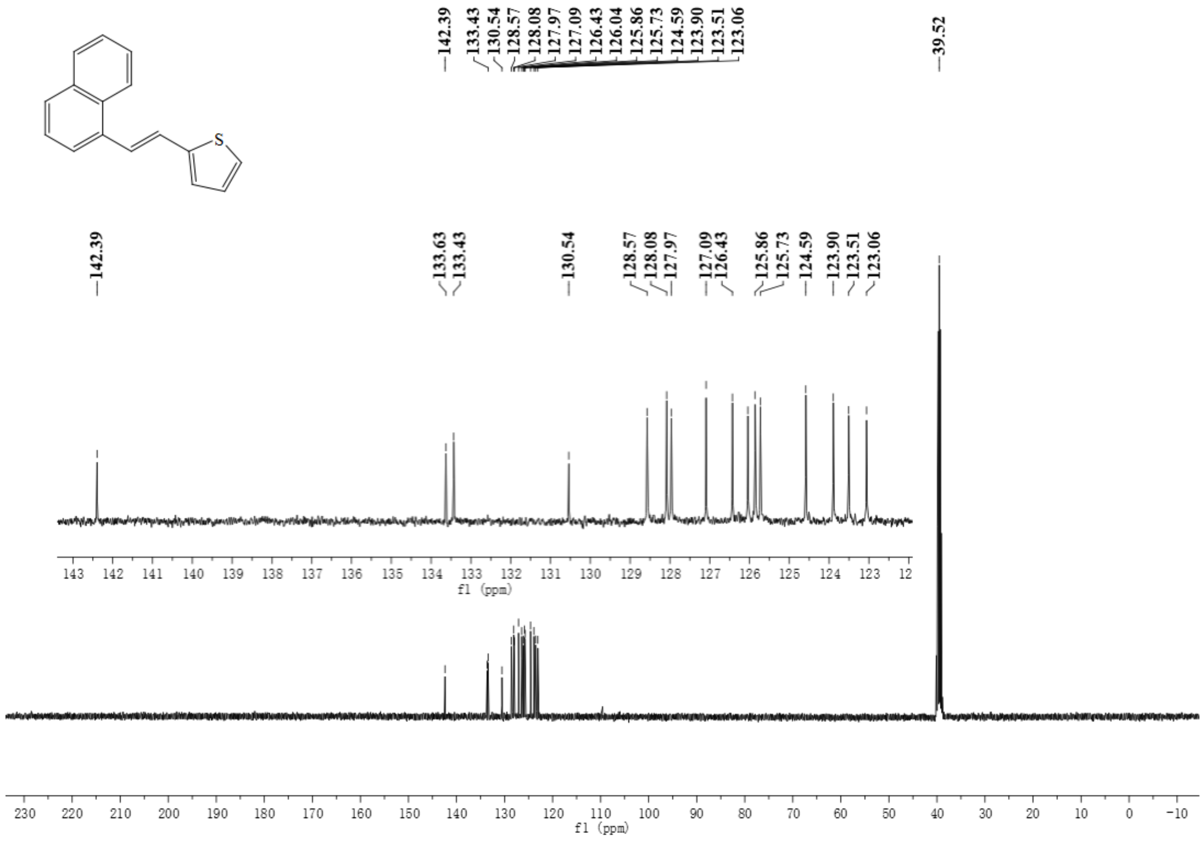

^{1}H NMR spectra of **4i** (DMSO-d_6)

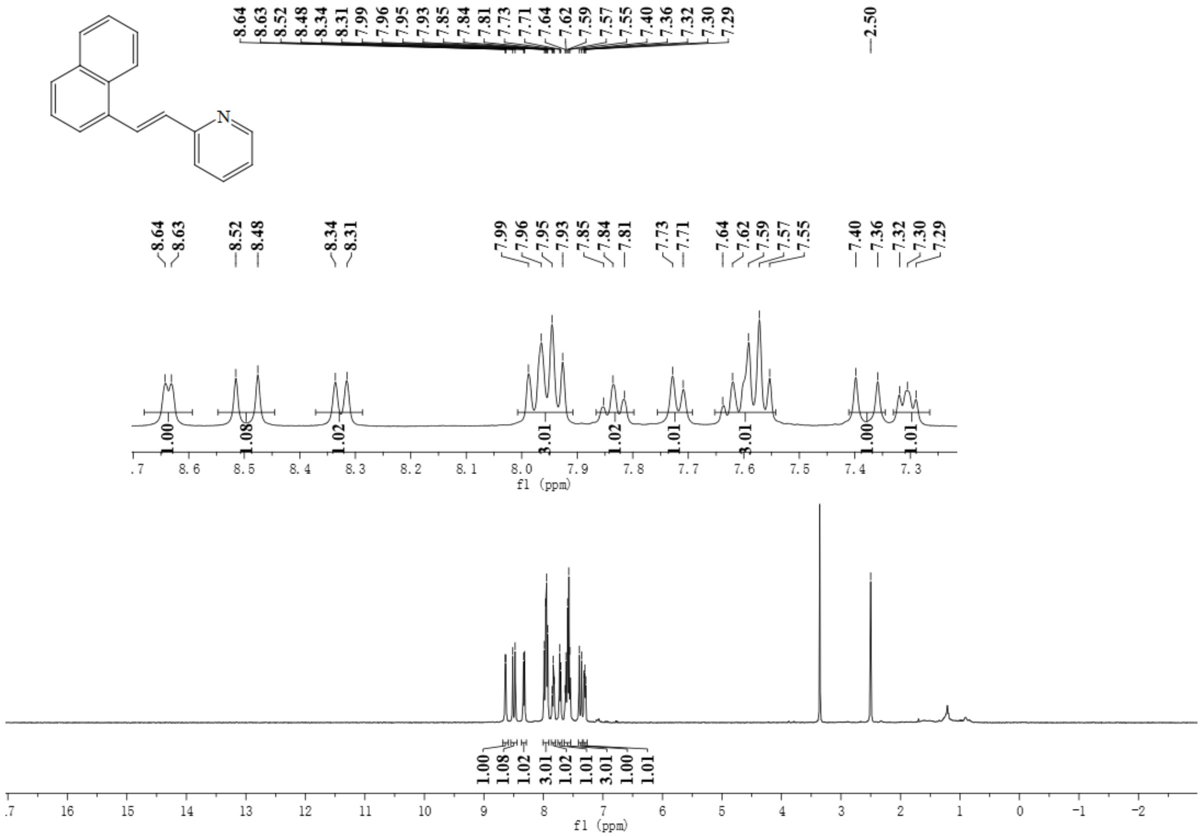

^{13}C NMR spectra of **4i** (DMSO-d_6)

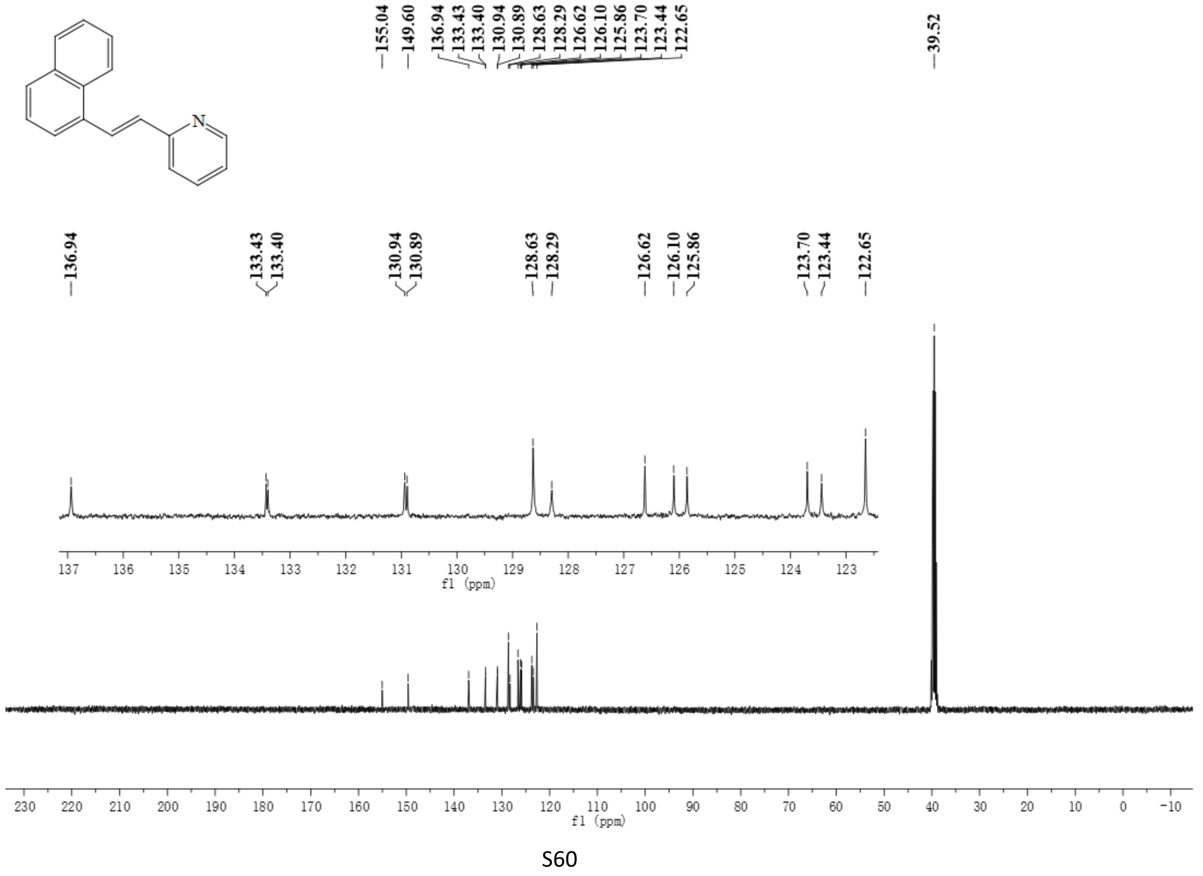

^{1}H NMR spectra of **4j** (DMSO-d_6)

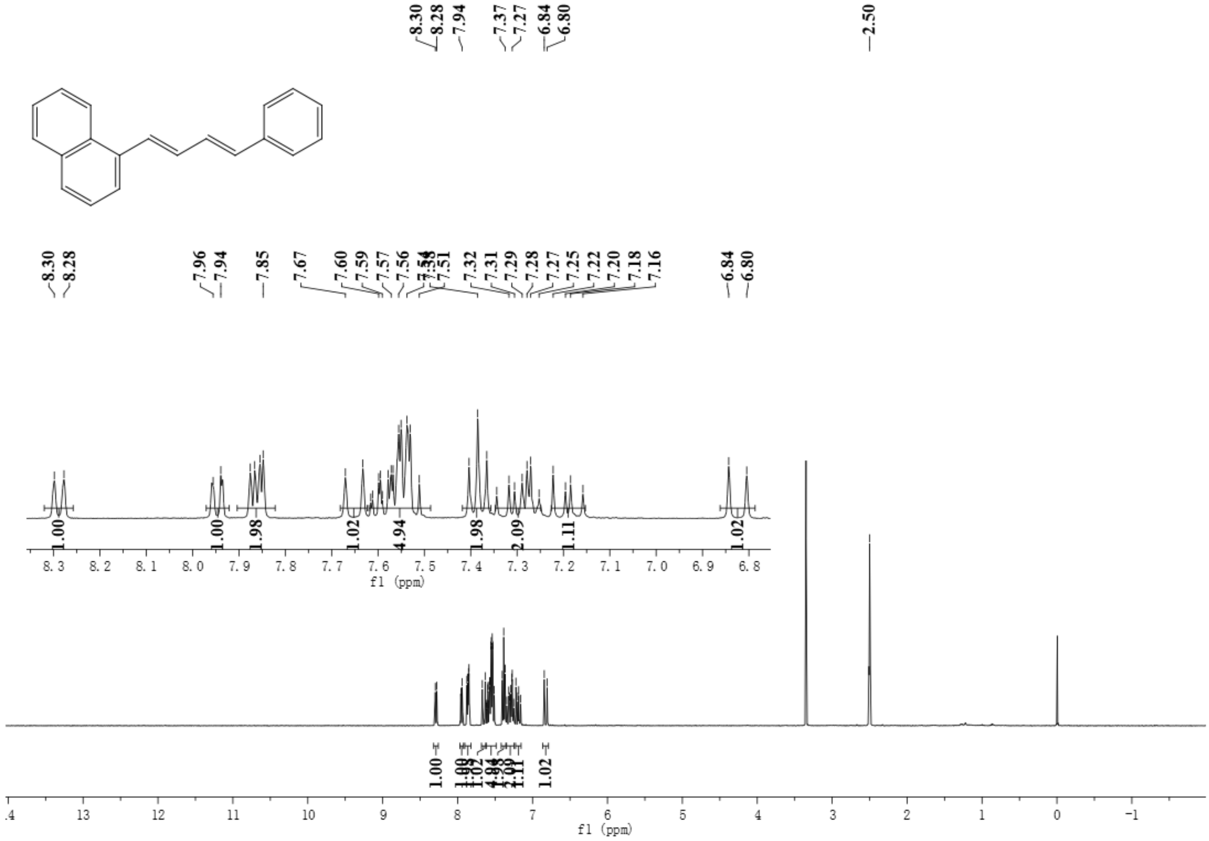

^{13}C NMR spectra of **4j** (DMSO-d_6)

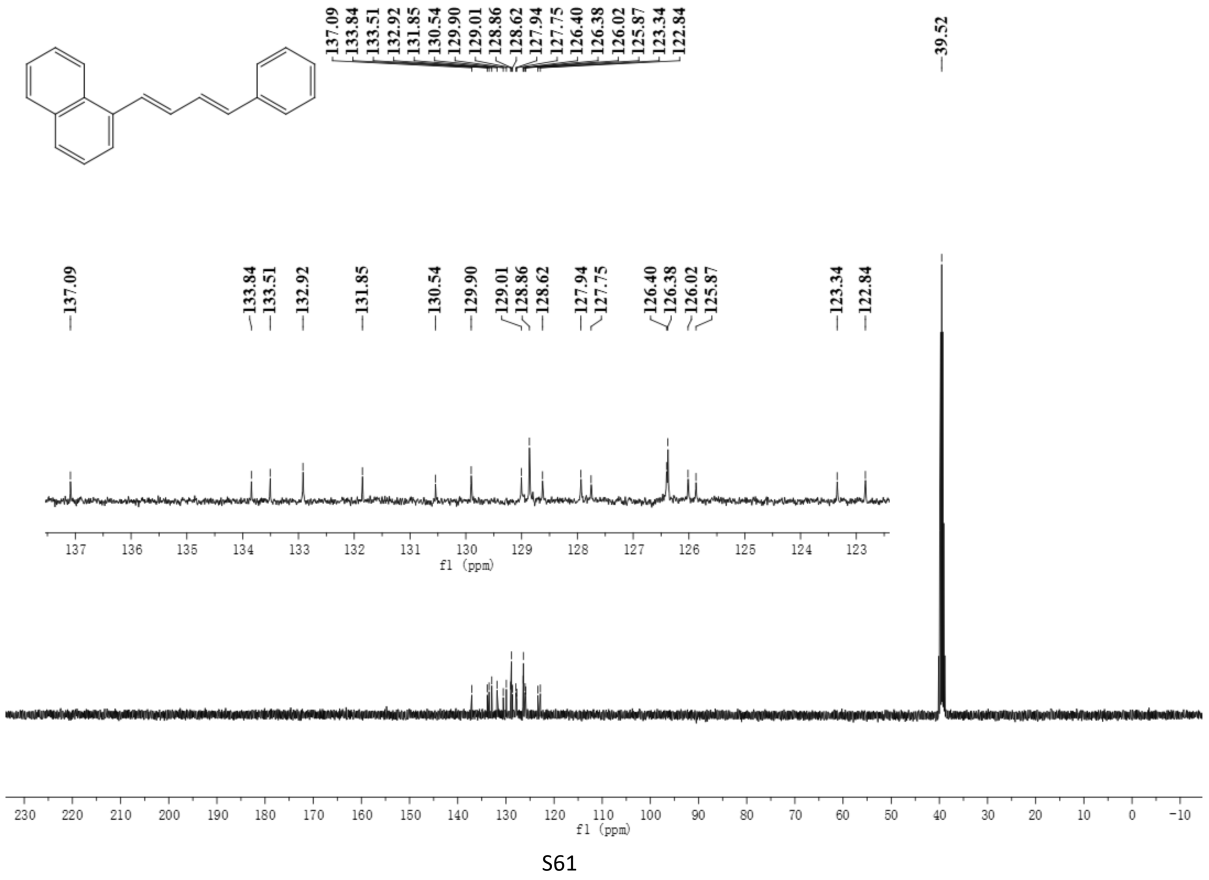

^{1}H NMR spectra of **4k** (DMSO-d_6)

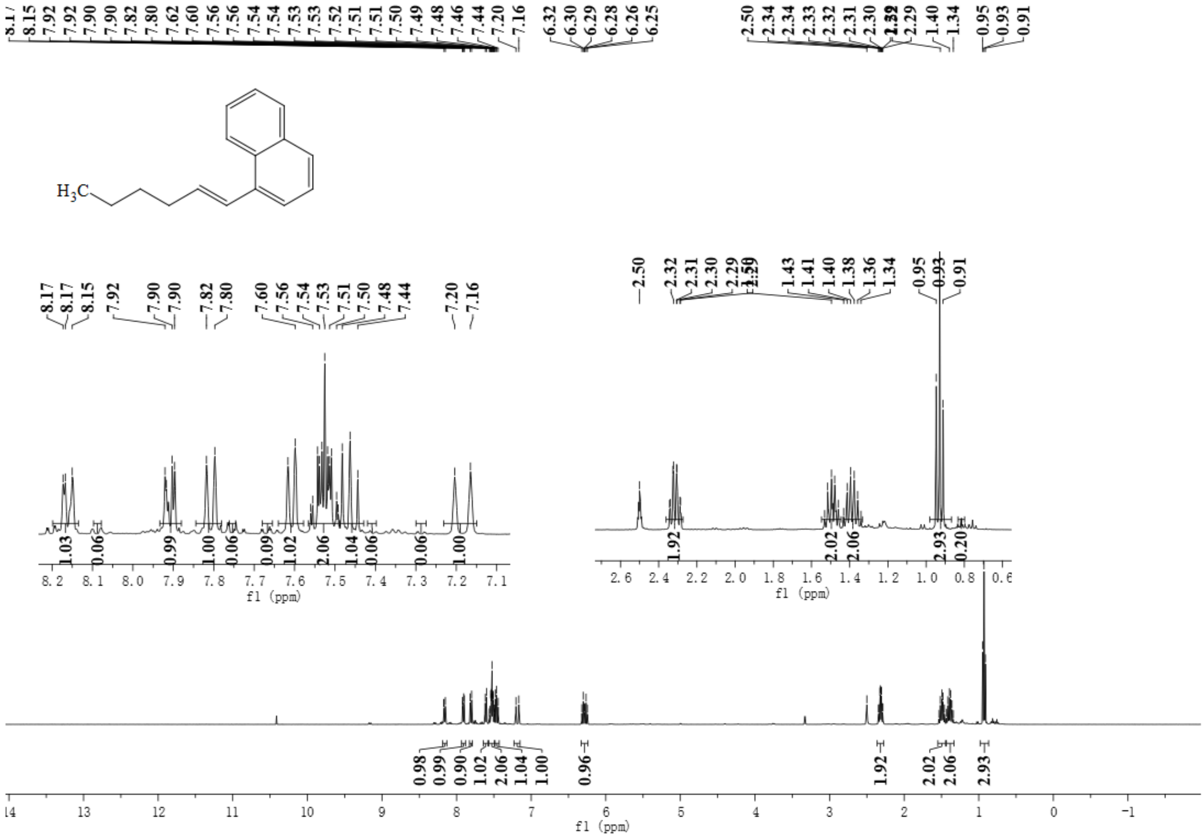

^{13}C NMR spectra of **4k** (DMSO-d_6)

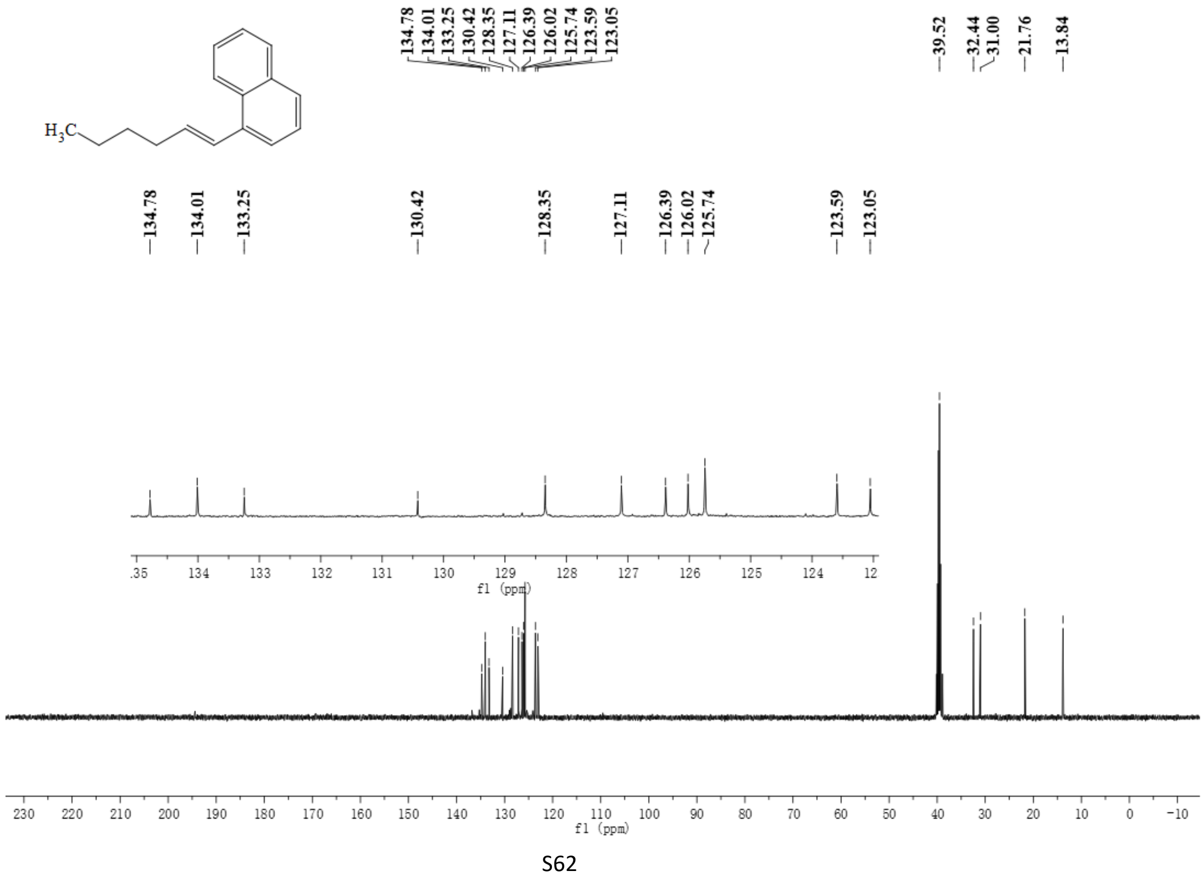

^{1}H NMR spectra of **4l** ($CDCl_3$)

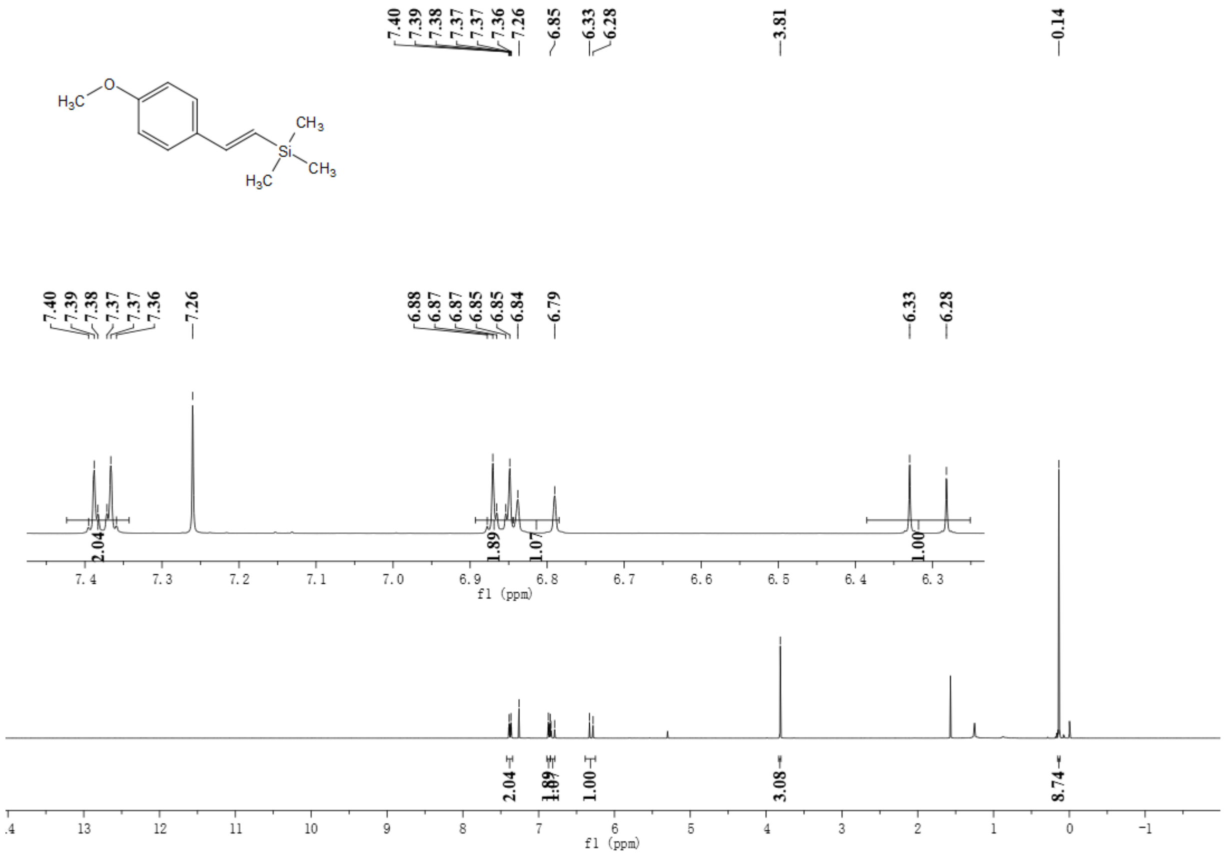

^{1}H NMR spectra of **4l** ($CDCl_3$)

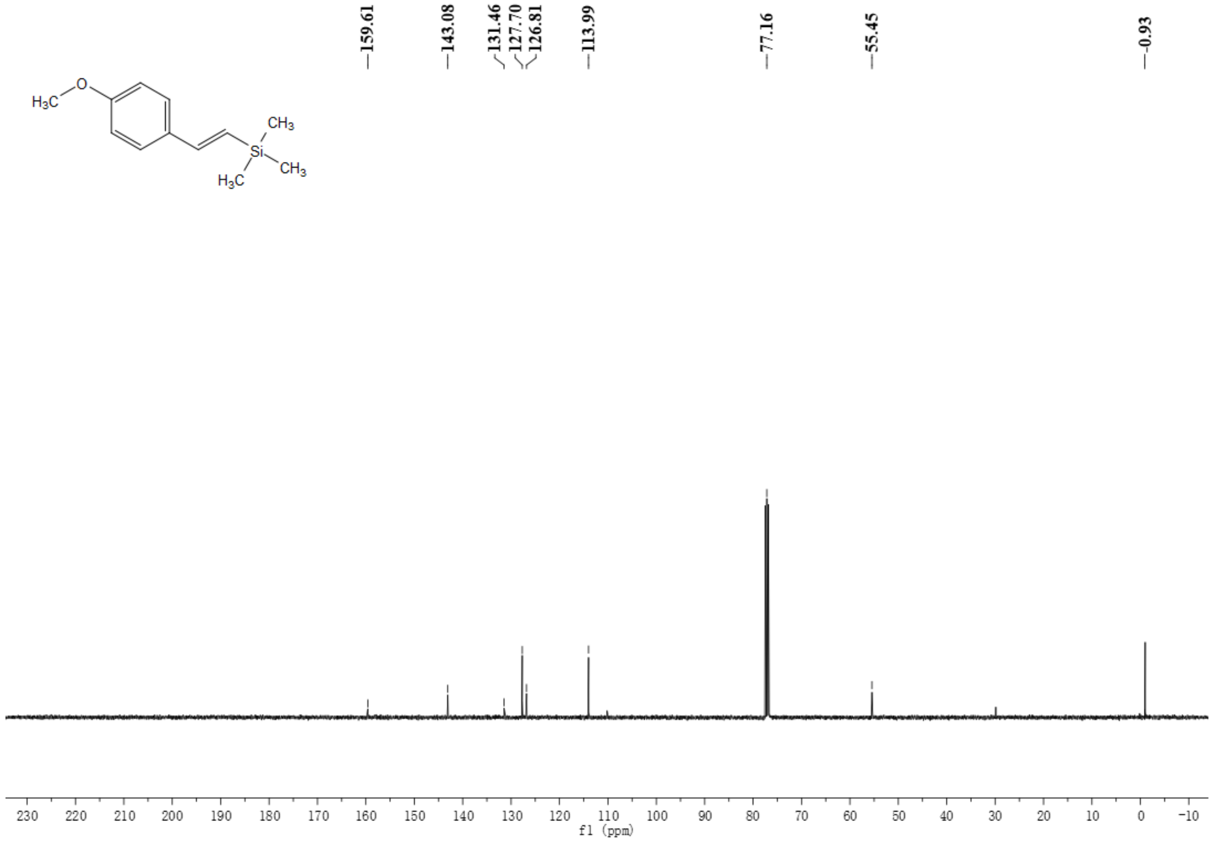

^{1}H NMR spectra of **5a** ($CDCl_3$)

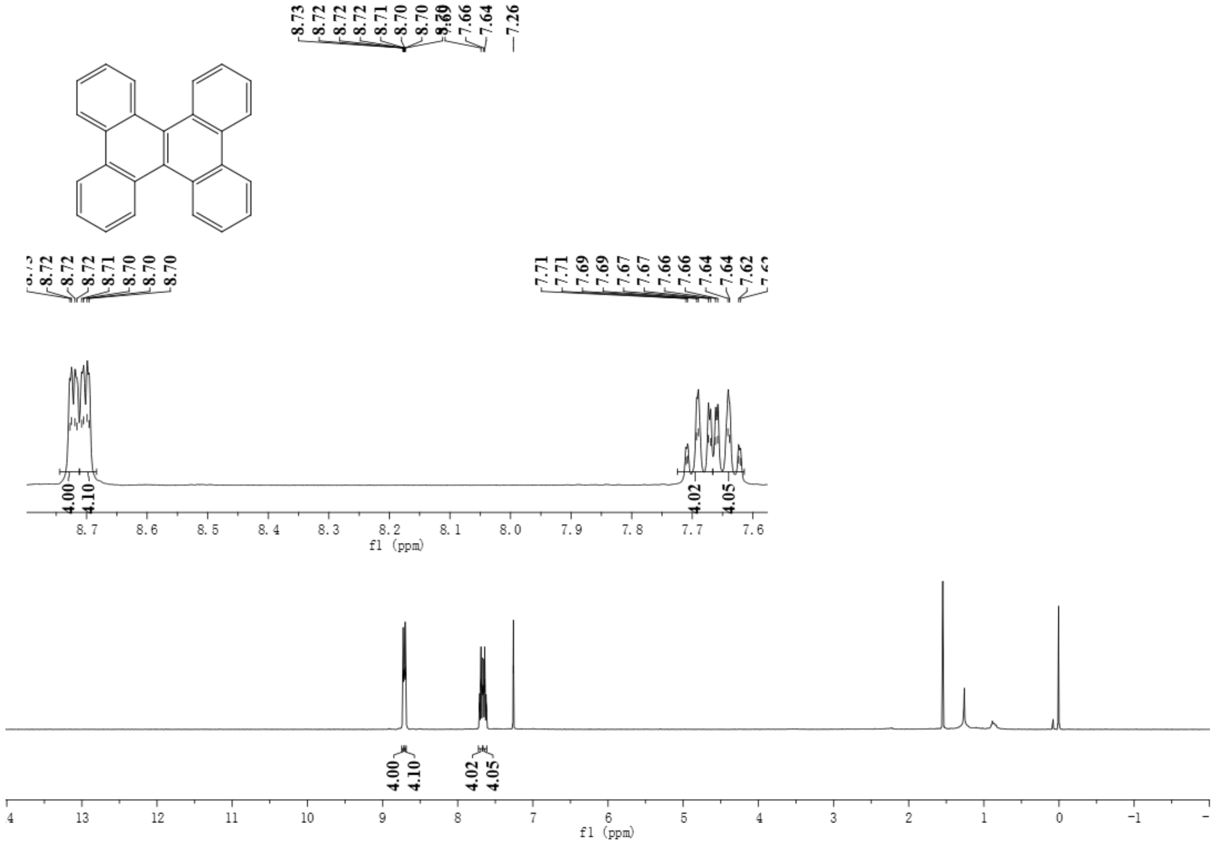

^{13}C NMR spectra of **5a** ($CDCl_3$)

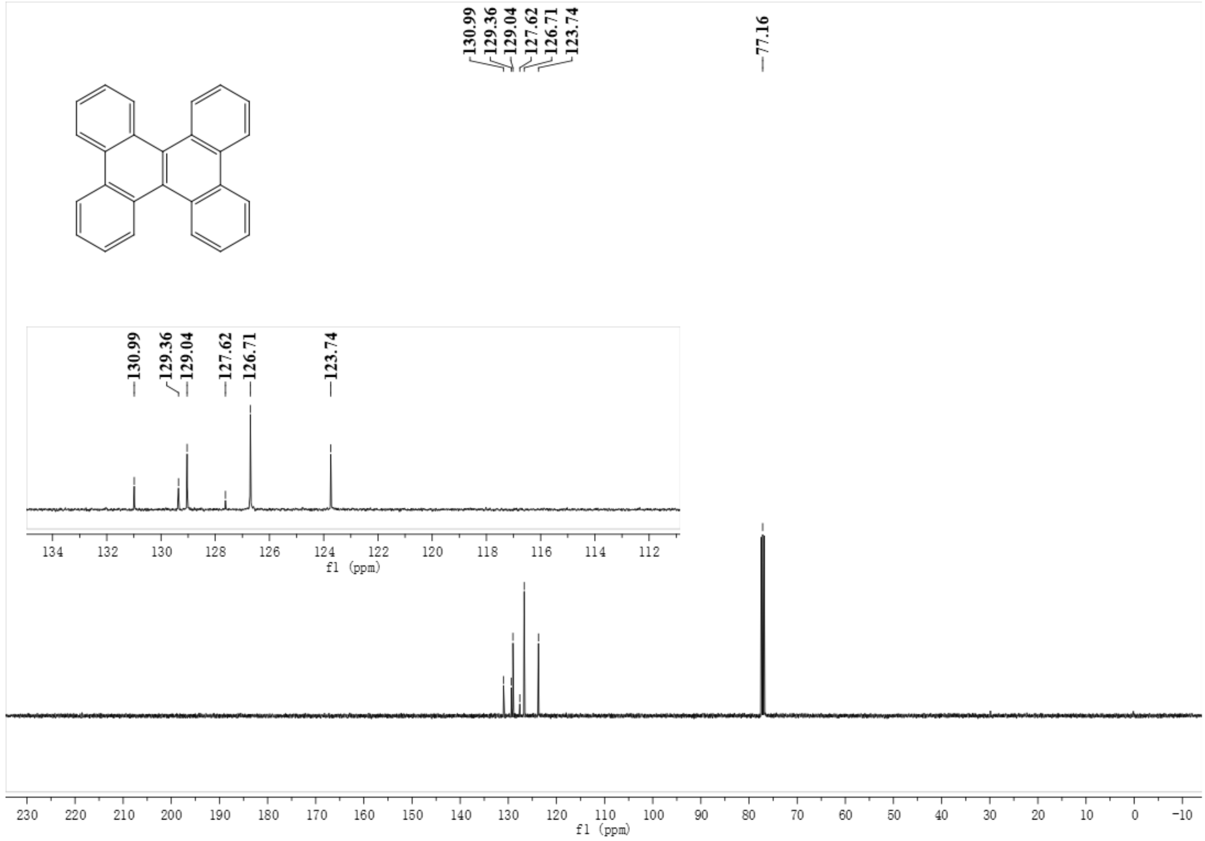

^{1}H NMR spectra of **5b’** ($CDCl_3$)

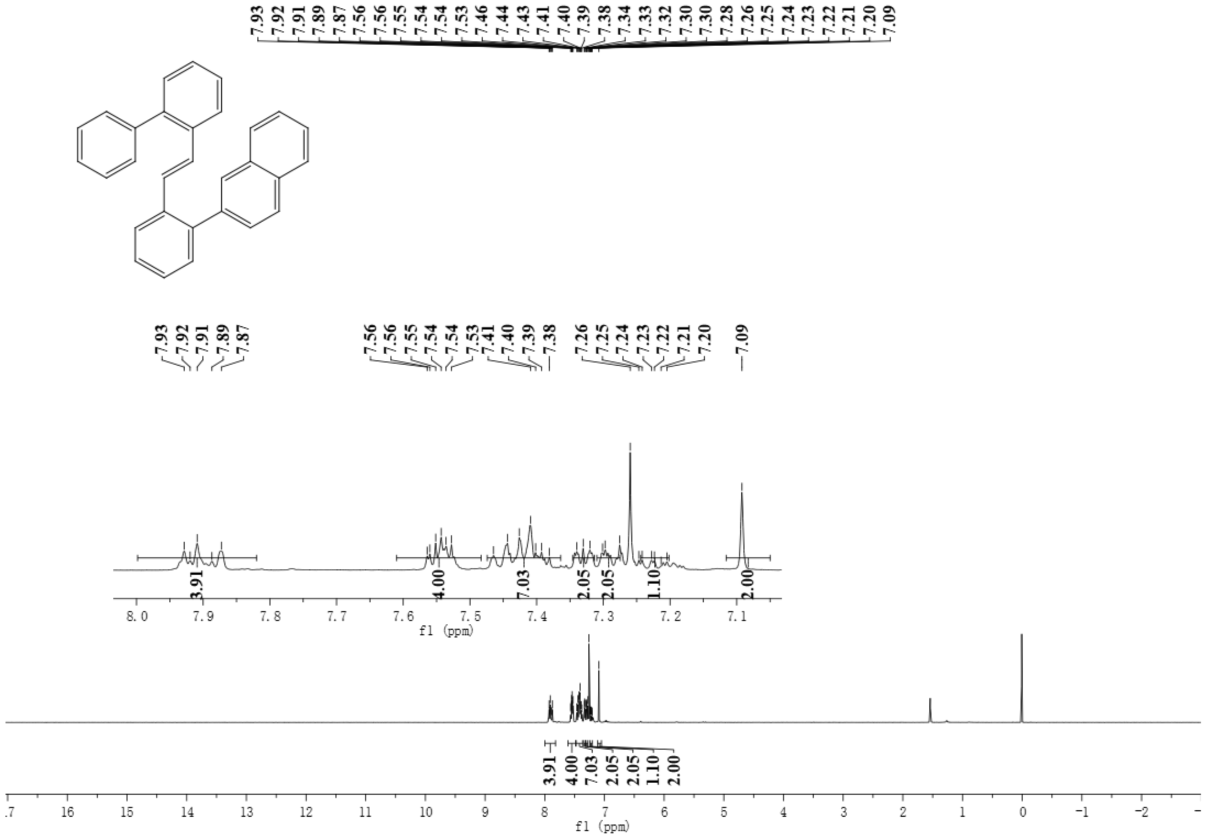

^{13}C NMR spectra of **5b’** ($CDCl_3$)

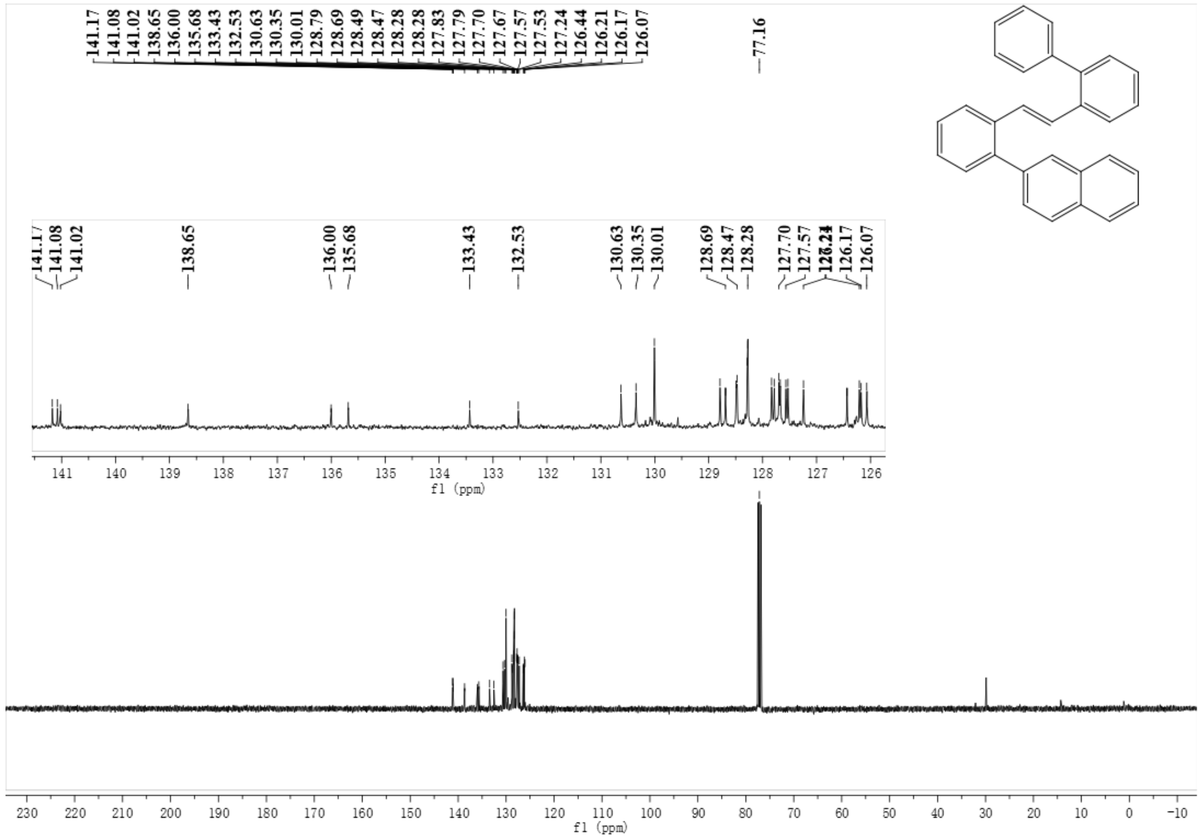

^{1}H NMR spectra of **5b** ($CDCl_3$)

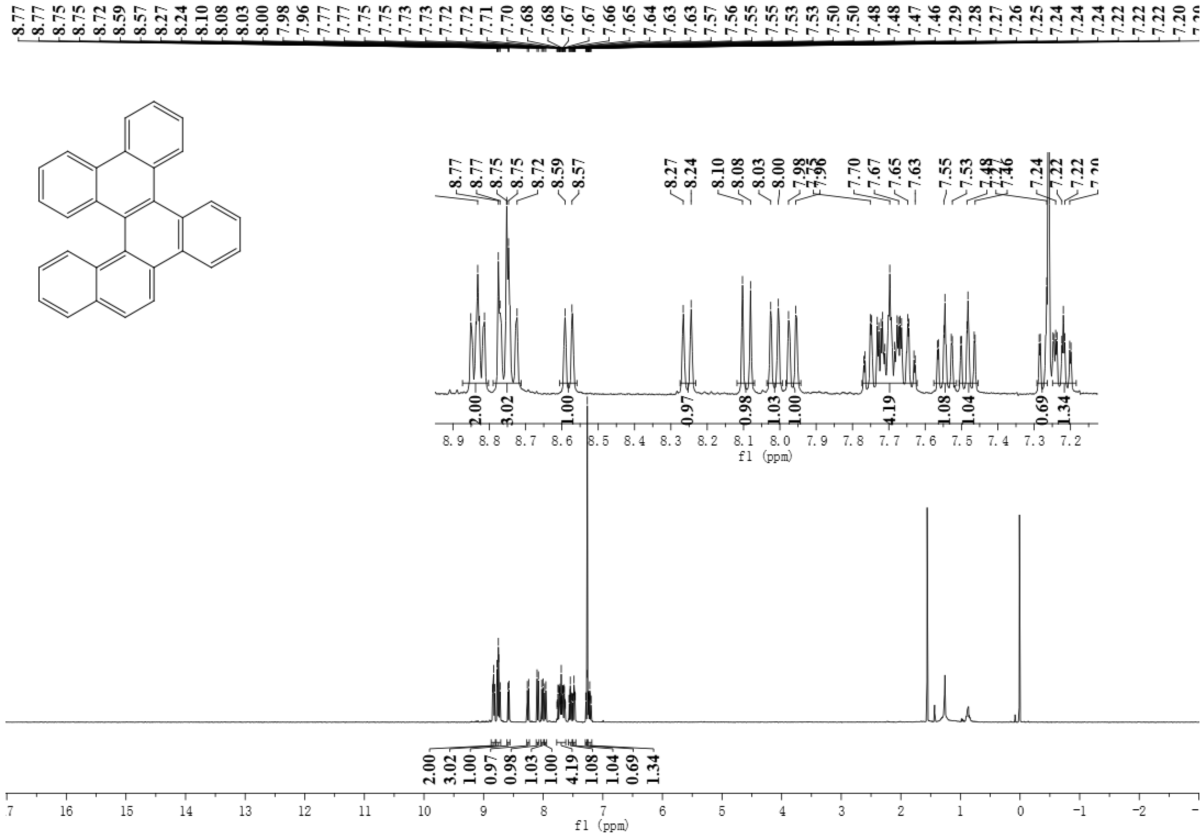

^{13}C NMR spectra of **5b** ($CDCl_3$)

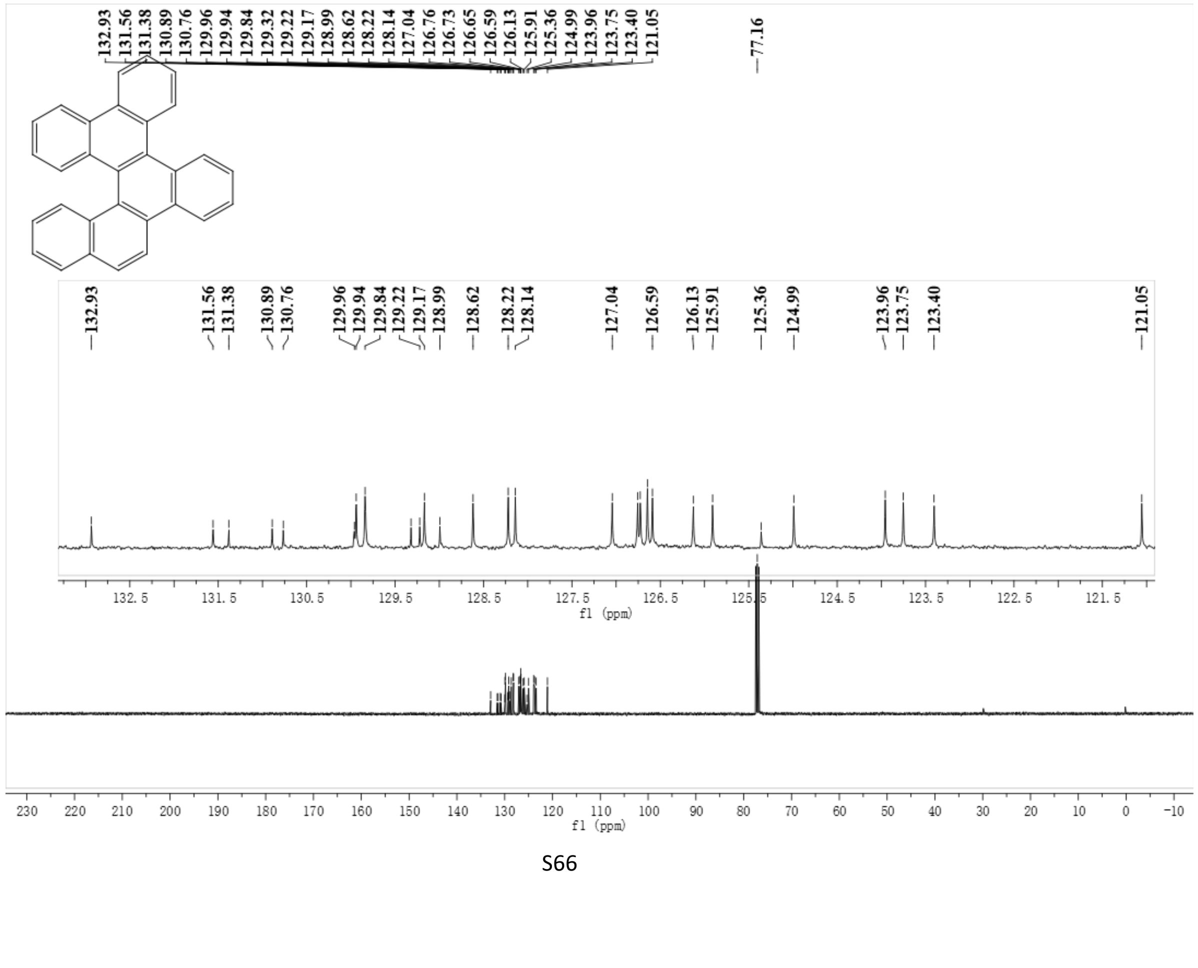

^{1}H NMR spectra of **5c** ($CDCl_3$)

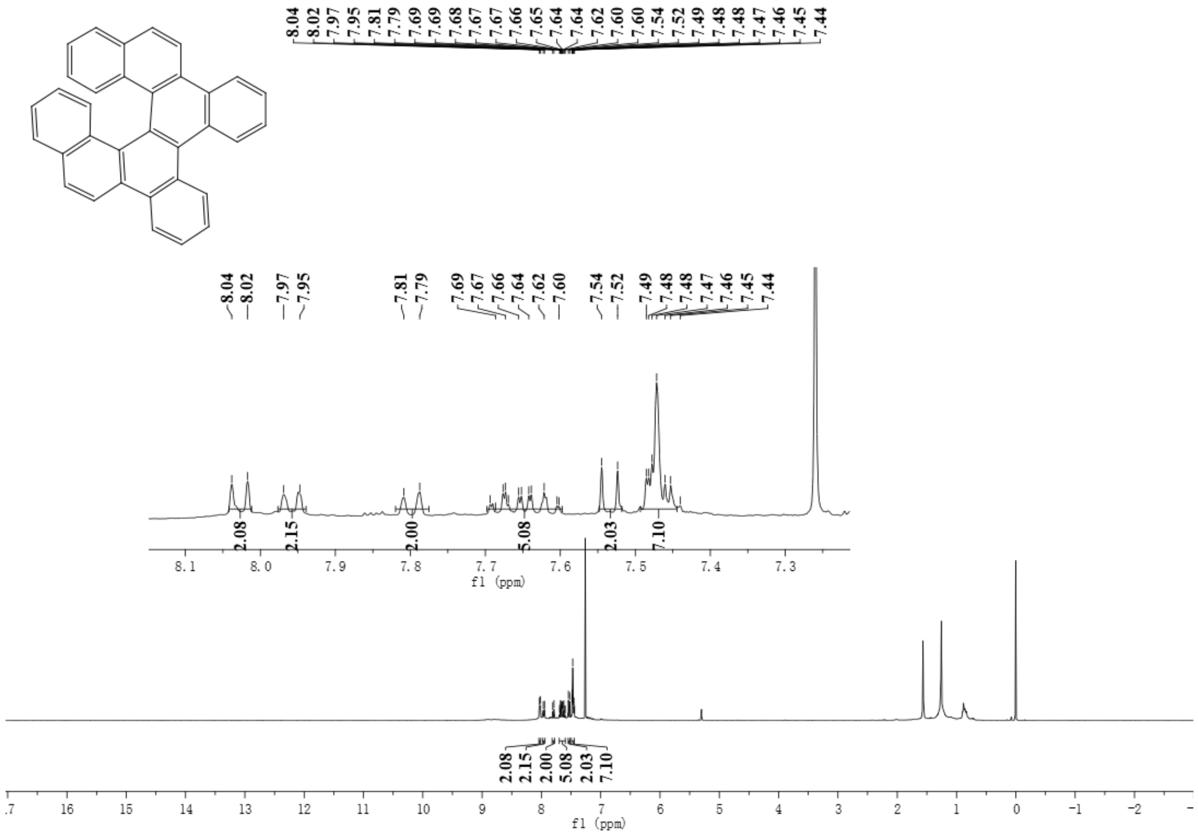

^{13}C NMR spectra of **5c** ($CDCl_3$)

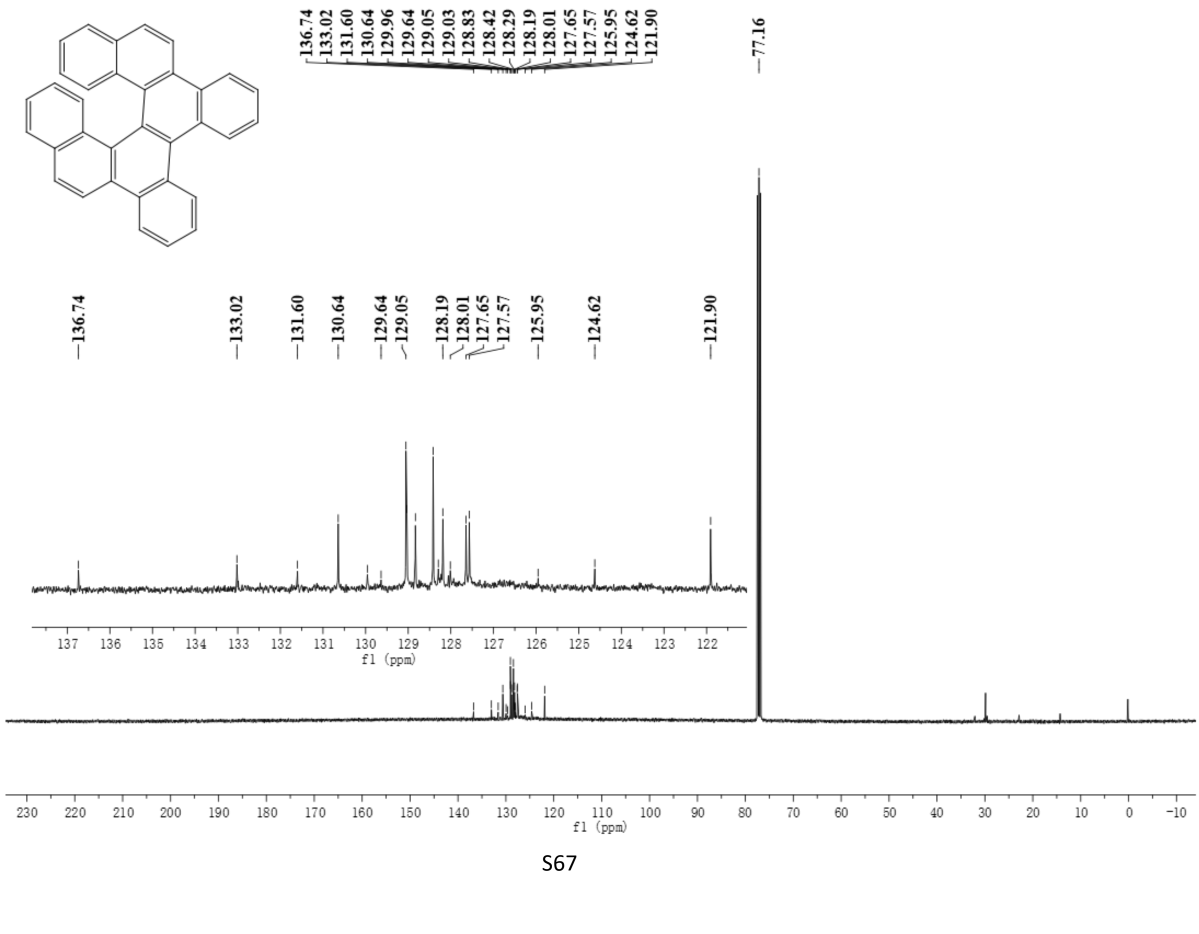

^{1}H NMR spectra of **5d** ($CDCl_3$)

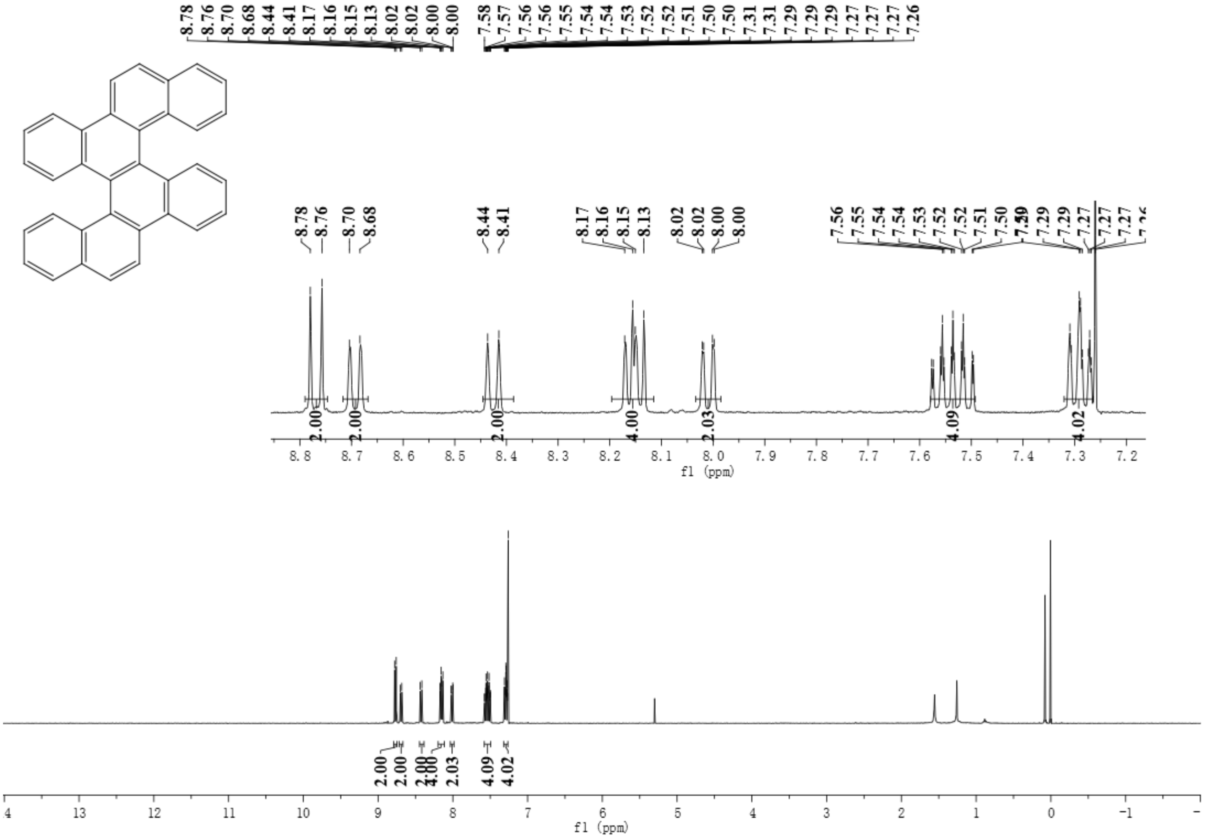

^{13}C NMR spectra of **5d** ($CDCl_3$)

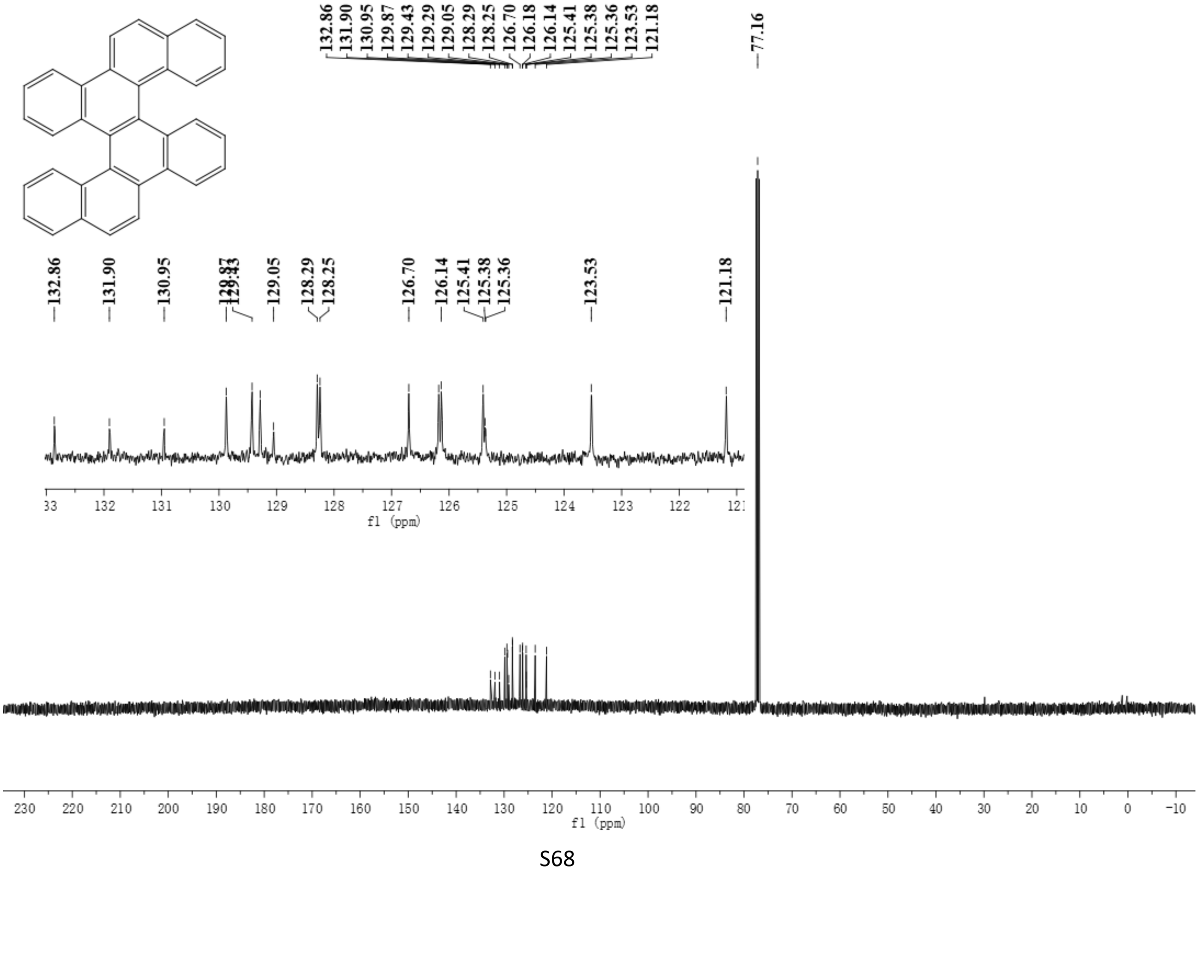

^{1}H NMR spectra of **5e** ($CDCl_3$)

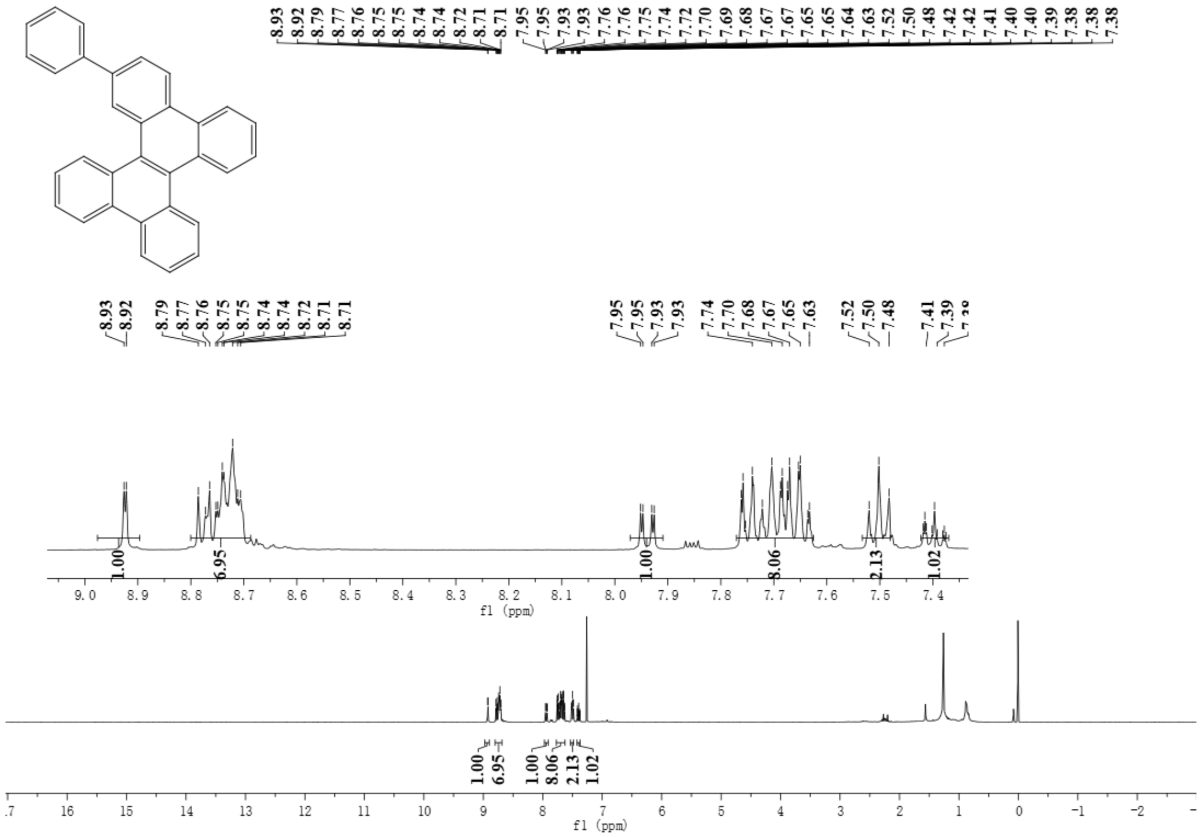

^{13}C NMR spectra of **5e** ($CDCl_3$)

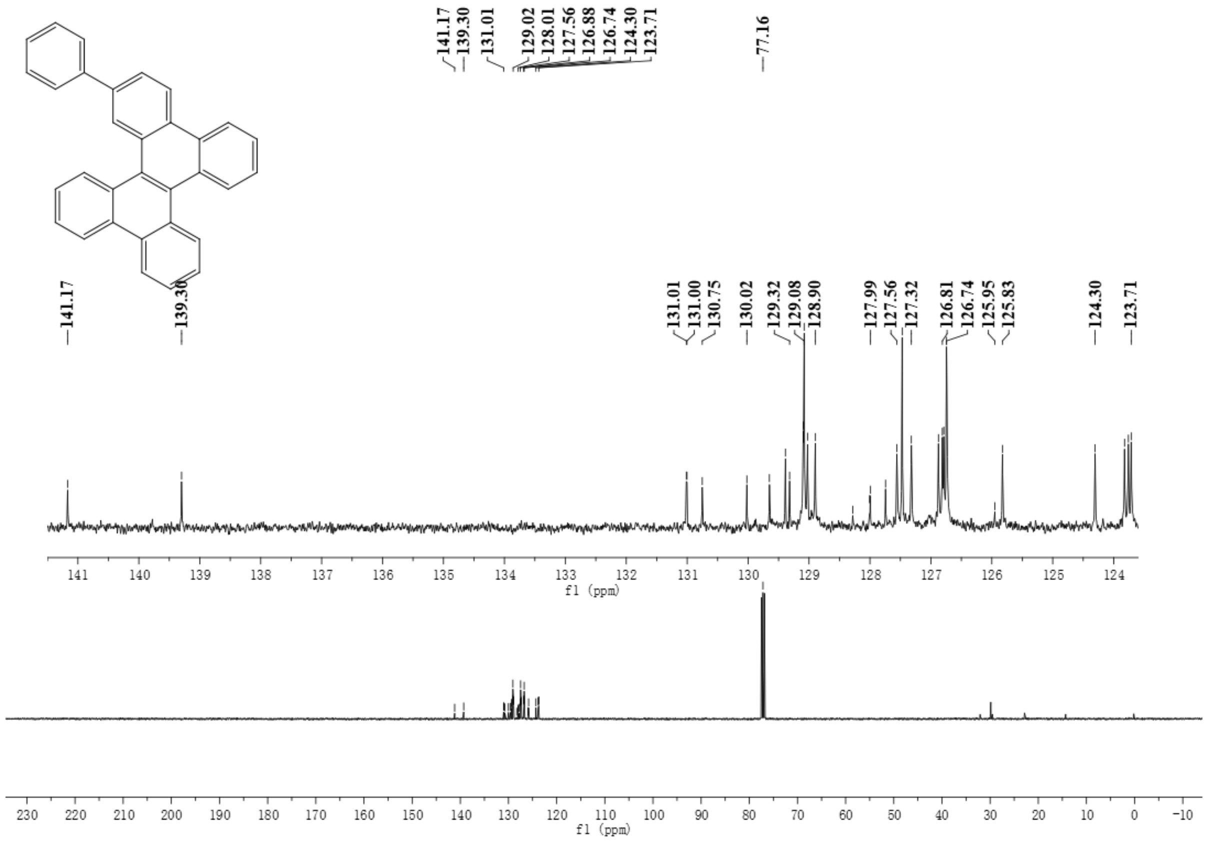

^{1}H NMR spectra of **5f** ($CDCl_3$)

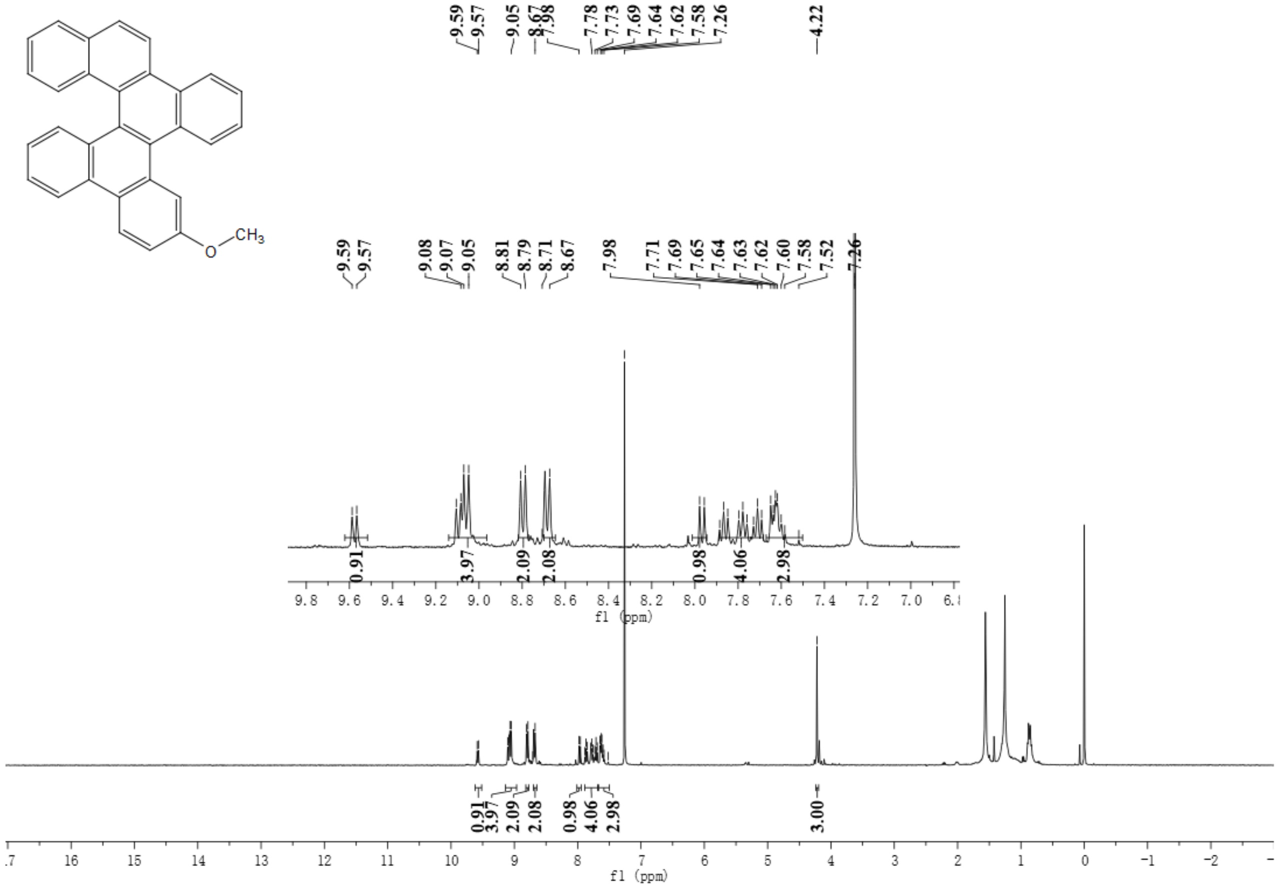

^{13}C NMR spectra of **5f** ($CDCl_3$)

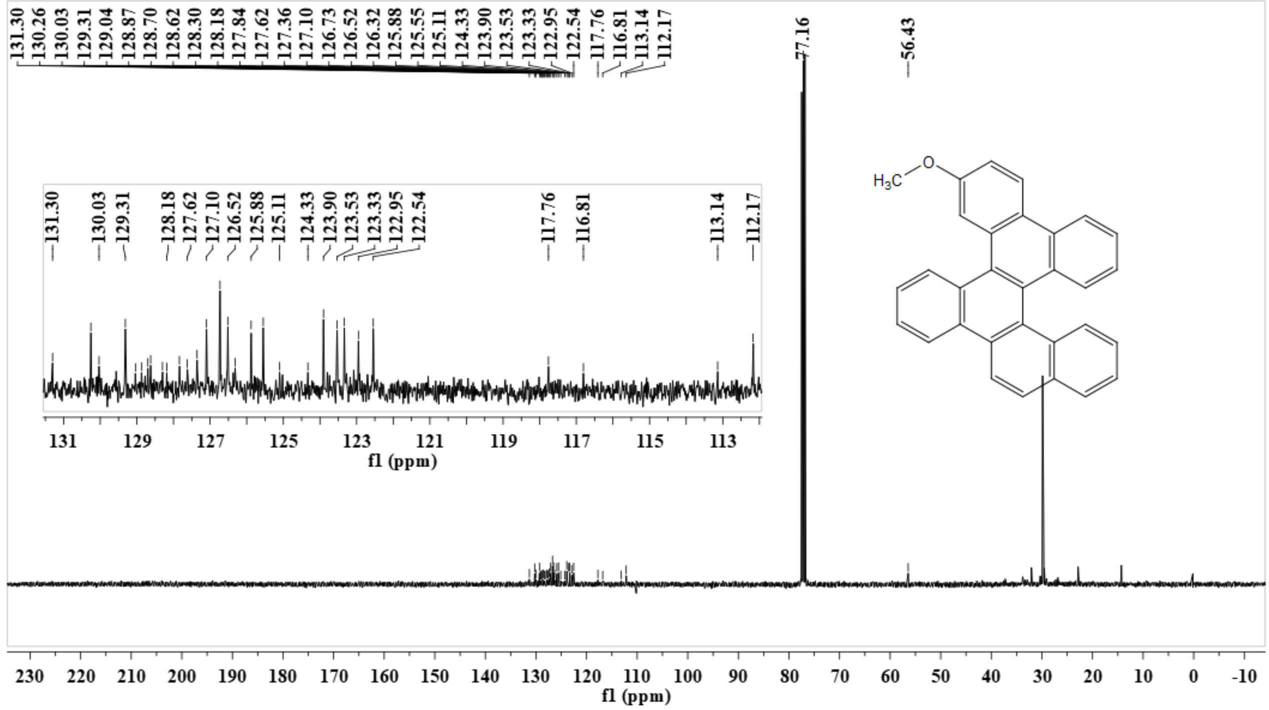

^{1}H NMR spectra of **5g** ($CDCl_3$)

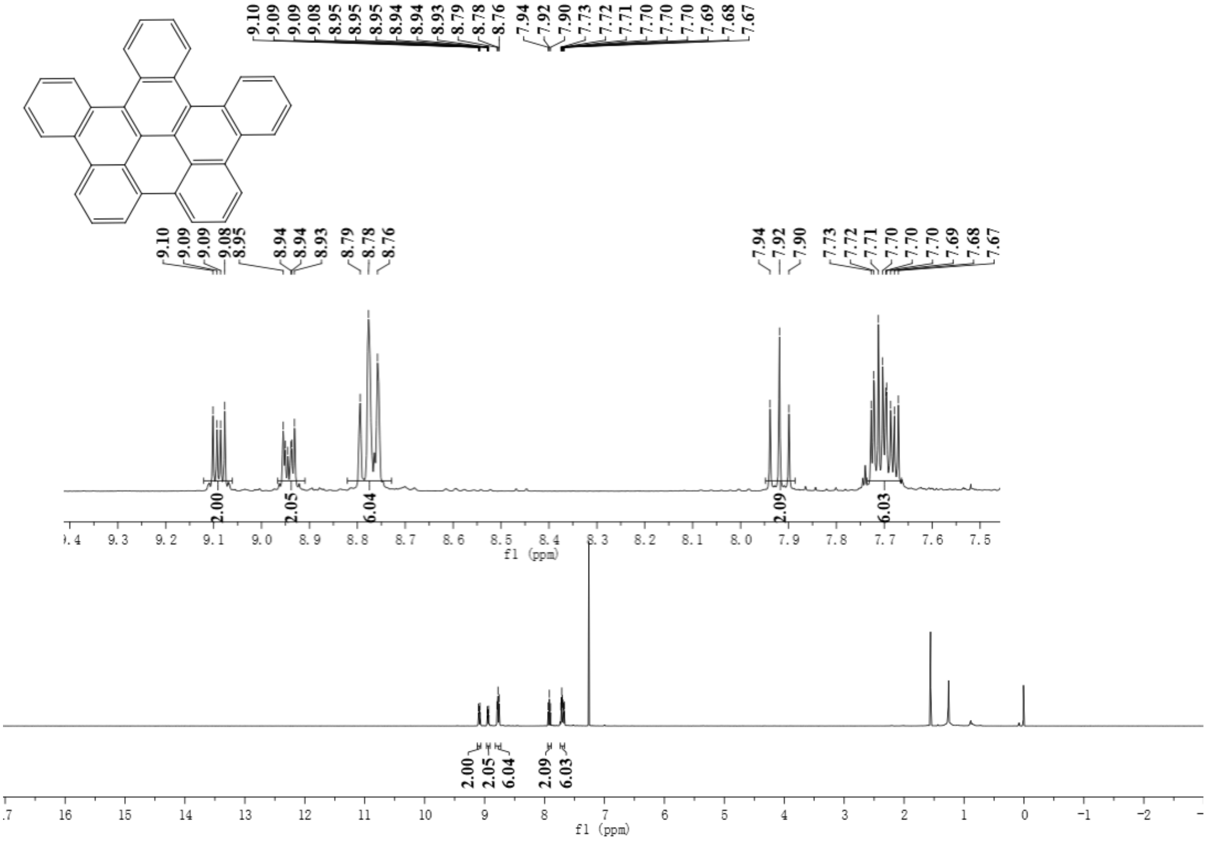

^{1}H NMR spectra of **5g** ($CDCl_3$)

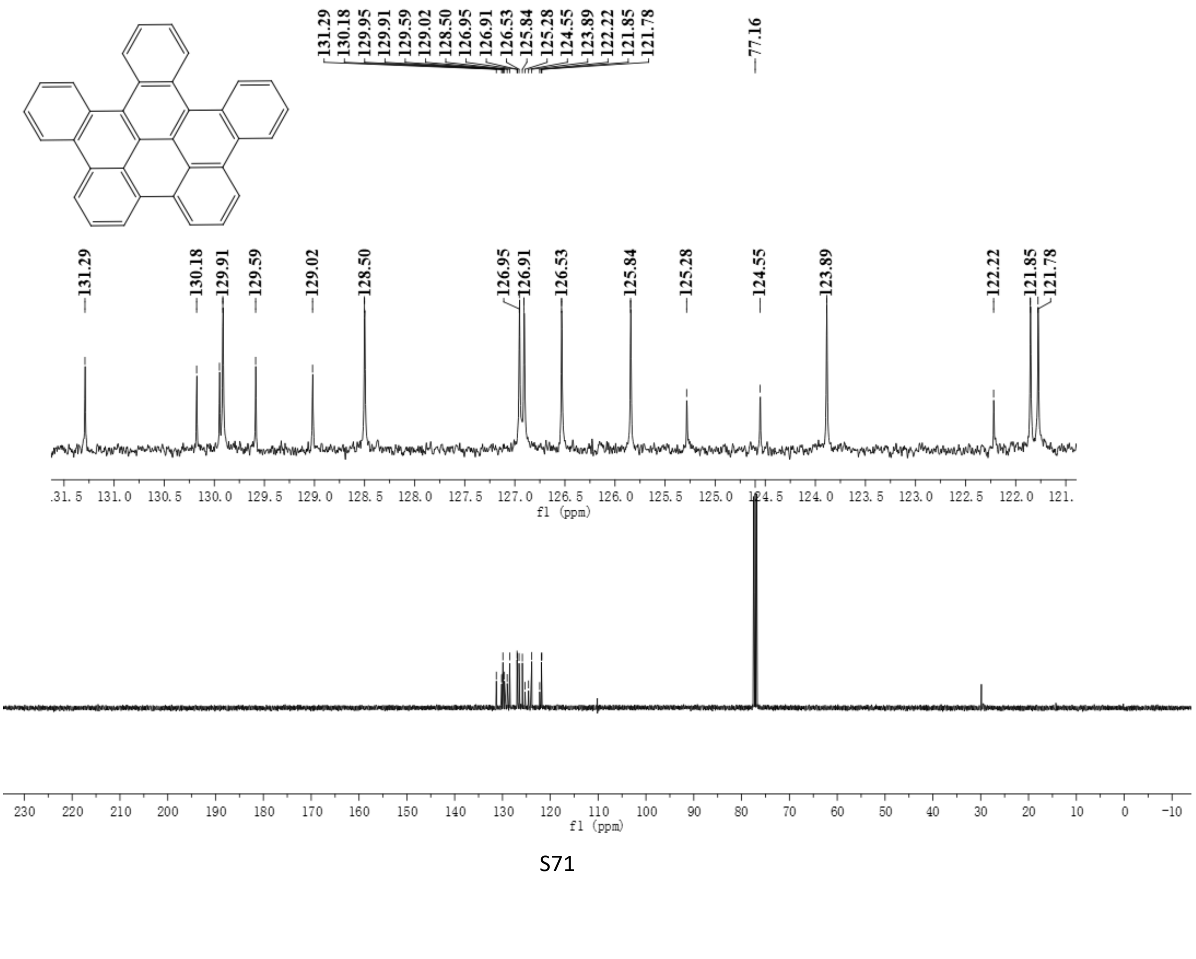

Supplement: SC-011-D0SC02058C-s001 [file SC-011-D0SC02058C-s001.pdf]
